# Novel Thiazolylketenyl Quinazolinones as Potential Anti-MRSA Agents and Allosteric Modulator for PBP2a

**DOI:** 10.3390/molecules28104240

**Published:** 2023-05-22

**Authors:** Jie Dai, Narsaiah Battini, Zhonglin Zang, Yan Luo, Chenghe Zhou

**Affiliations:** 1 Institute of Bioorganic & Medicinal Chemistry, Key Laboratory of Applied Chemistry of Chongqing Municipality, School of Chemistry and Chemical Engineering, Southwest University, Chongqing 400715, China; daij2020@email.swu.edu.cn (J.D.); narsaiah.org.chem@gmail.com (N.B.); 2 College of Pharmacy, National & Local Joint Engineering Research Center of Targeted and Innovative Therapeutics, Chongqing Key Laboratory of Kinase Modulators as Innovative Medicine, Chongqing University of Arts and Sciences, Chongqing 402160, China

**Keywords:** quinazolinone, thiazole, antibacterial, resistance, PBP2a

## Abstract

Bacterial infections caused by methicillin-resistant *Staphylococcus aureus* have seriously threatened public health. There is an urgent need to propose an existing regimen to overcome multidrug resistance of MRSA. A unique class of novel anti-MRSA thiazolylketenyl quinazolinones (TQs) and their analogs were developed. Some synthesized compounds showed good bacteriostatic potency. Especially TQ **4** was found to exhibit excellent inhibition against MRSA with a low MIC of 0.5 μg/mL, which was 8-fold more effective than norfloxacin. The combination of TQ **4** with cefdinir showed stronger antibacterial potency. Further investigation revealed that TQ **4,** with low hemolytic toxicity and low drug resistance, was not only able to inhibit biofilm formation but also could reduce MRSA metabolic activity and showed good drug-likeness. Mechanistic explorations revealed that TQ **4** could cause leakage of proteins by disrupting membrane integrity and block DNA replication by intercalated DNA. Furthermore, the synergistic antibacterial effect with cefdinir might be attributed to TQ **4** with the ability to induce PBP2a allosteric regulation of MRSA and further trigger the opening of the active site to promote the binding of cefdinir to the active site, thus inhibiting the expression of PBP2a, thereby overcoming MRSA resistance and significantly enhancing the anti-MRSA activity of cefdinir. A new strategy provided by these findings was that TQ **4**, possessing both excellent anti-MRSA activity and allosteric effect of PBP2a, merited further development as a novel class of antibacterial agents to overcome increasingly severe MRSA infections.

## 1. Introduction

The discovery of antibiotics has effectively controlled the hazards posed by bacterial infections that have plagued humans for millennia. However, the uninterrupted use of antibiotics inevitably brings the gradual emergence of bacterial resistance [1,2]. Especially the emergence and clonal spread of methicillin-resistant *Staphylococcus aureus* (MRSA), which is associated with high morbidity and mortality, has aroused public concern about health [3,4]. The main reason for the troublesome treatment of MRSA is the resistance to all known *β*-lactam antibiotics, which is related to the presence of penicillin-binding protein 2a (PBP2a) encoded by mecA gene in the chromosome [5,6,7]. PBP2a has been shown to harbor a sheltered active site. Only when the allosteric site is occupied by the active molecule to trigger a conformational change, *β*-lactam antibiotics can gain access to the opened active site to exert their antibacterial effect [8,9]. Currently, oxadiazoles, flavonoids, naphthalimide, and quinazolinones have been proven to serve as allosteric triggers [10,11]. This fact undoubtedly encourages researchers to exploit more allosteric site binding molecules, in combination with old drugs, that restore the activity of deactivated antibiotics.

Quinazolinones, as a group of natural alkaloids, are the main structural units in traditional Chinese medicine (TCM). Quinazolinones have shown great medicinal potential because of their excellent biological activities and environmental friendliness, which have attracted abundant attention [12,13,14]. Specifically, in the field of antibacterial aspects, the combination of quinazolinone with other pharmacophores exhibits excellent antibacterial activity [15,16]. Moreover, quinazolinones have been proven to be able to interact with the allosteric sites of PBP2a to trigger conformational changes. Hence, quinazolinones may not only act as antibacterial agents but also be designed as triggers for the allosteric site of PBP2a in combination with drugs to exert potent antibacterial inhibition, which can be considered a novel design strategy.

Thiazole is a quite important five-membered aromatic heterocycle with molecular formula C_3_H_3_NS, characterized by both an electron donating group (-S-) and an electron accepting group (C=N) [17,18]. The quite electron-rich conjugation system of thiazole heterocycle enables it to combine with multiple functional targets to improve biological activity, resulting in widespread appreciation and cultivation in drug design and development [19,20], and some of them provide enormous contributions to successful development of clinical antibiotics, such as the third and fourth cephalosporins and sulfathiazole. Our previous research revealed that the combination of quinazolinone and thiazoles delivered great antibacterial significance [21,22]. Moreover, thiophene, furan, and the fused derivatives are prevalently regarded as important biological isosteres of thiazole in drug design with great development potentiality in organic and pharmaceutical chemistry [23,24]. Therefore, they were introduced with the aim of discovering novel potential antibacterial molecules with broad spectrum and high biological activity.

Ethenones, as a class of important natural drug scaffolds, showed unique fascination in the pharmaceutical field [25,26]. Ethenone serves as a bridging group that enables the increase of the conjugation system that facilitates inserting into DNA to prevent replication of bacterial DNA [27,28]. Furthermore, previous literature has shown that attachment of a benzonitrile via an olefinic bond at the 2-position of the quinazolinone can interact with the allosteric site of PBP2a, suggesting that large conjugated systems may be allosteric site-binding molecules for PBP2a [29,30]. Thus, the conjugates with large conjugated systems formed by introducing thiazoles and their isosteres into quinazolinone through ethenone may have an interaction with the allosteric site of PBP2a, triggering a conformational change.

**Figure 1:**
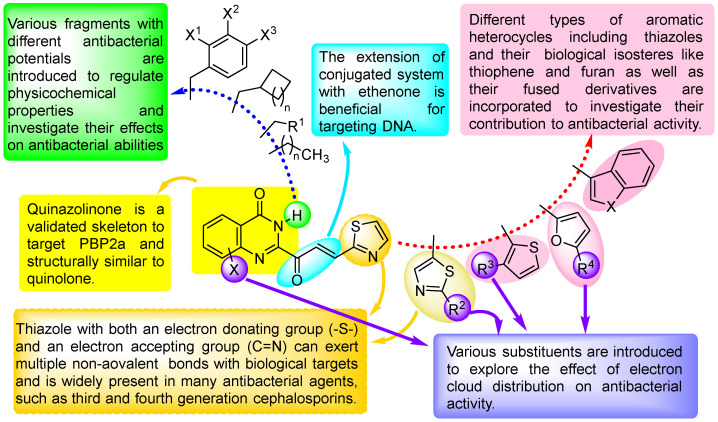
Design of thiazolylketenyl quinazolinones and analogs as potential antibacterial agents and allosteric molecules of PBP2a.

In view of the above observations, ethenone as a linker was employed to couple quinazolinone and thiazoles and their isosteres to generate a unique structural type of novel thiazolylketenyl quinazolinones and analogs, which might have good antibacterial efficacy to overcome the global increasing bacterial drug resistance. Therefore, a series of new thiazolylketenyl quinazolinones and analogs was prepared through multistep procedures from 2-aminobenzamide, various types of substituents were considered in order to investigate the structure–activity relationships (Figure 1).

All the synthesized target thiazolylketenyl quinazolinones and analogs were tested for antibacterial activity according to Clinical and Laboratory Standards Institute, and the selected highly active molecule was evaluated for the synergistic antibacterial effect with clinical cefdinir, hemolytic activity, drug resistance development, as well as ADMET simulation in order to investigate the potential to be developed as drugs [31,32]. Furthermore, the inhibition of biofilm growth ability and detection of metabolic activity were measured to investigate its bacteriostatic ability. Next, a series of preliminary mechanisms of action was explored, including the damage of membrane, leakage of intracellular protein, oxidative stress, and interaction with DNA [33,34]. Moreover, allosteric modulation experiments of PBP2a on highly active molecules were assayed to explore the possibility of the thiazolylketenyl quinazolinones being an allosteric molecule of PBP2a, and the possible binding mode to the allosteric site was calculated by molecular docking.

## 2. Results and Discussion

### 2.1. Chemistry

**Scheme 1:**
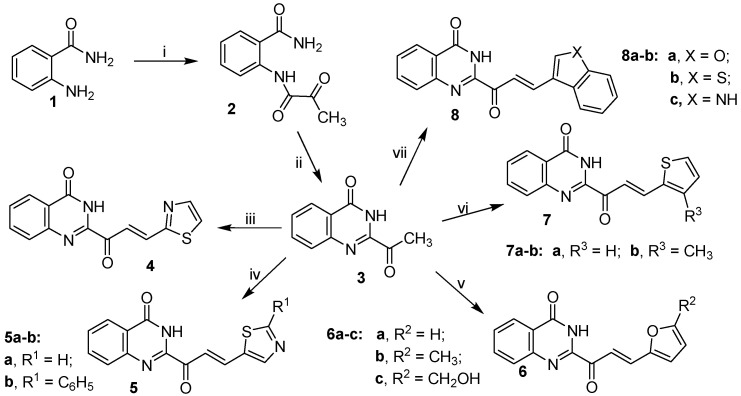
Preparation of thiazolylketenyl quinazolinones and analogs **4**–**8**. Reagents and conditions: (i) Pyruvic acid, 1-(3-dimethylaminopropyl)-3-ethylcarbodiimide hydrochloride, 1-hydroxybenzotriazole hydrate, DMF, r.t.; (ii) 10% NaOH aqueous solution, r.t.; (iii) 2-thiazolecarboxaldehyde, piperidine, CH_3_CH_2_OH, 80 °C; (iv) 5-thiazole aldehydes, piperidine, CH_3_CH_2_OH, 80 °C; (v) furan aldehydes, piperidine, CH_3_CH_2_OH, 80 °C; (vi) thiophene aldehydes, piperidine, CH_3_CH_2_OH, 80 °C; (vii) benzo[b]thiophene-2-carboxaldehyde or benzofuran-2-carboxaldehyde or indole-3-carboxaldehyde, piperidine, CH_3_CH_2_OH, 80 °C.

**Scheme 2:**
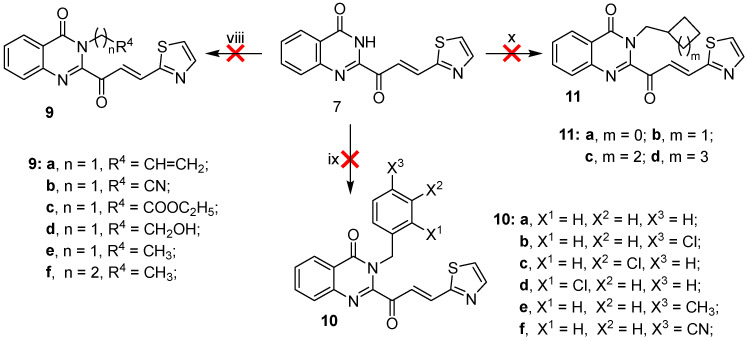
Preparation of thiazolylketenyl quinazolinones **9**–**11** with different substituents at *N*-3 position of quinazolinone. Reagents and conditions: (viii) Unsaturated halides or alkyl bromide, K_2_CO_3_, CH_3_CN, 80 °C; (ix) substituted benzyl chloride, K_2_CO_3_, CH_3_CN, 80 °C; (x) cycloalkyl bromide, K_2_CO_3_, CH_3_CN, 80 °C.

**Scheme 3:**
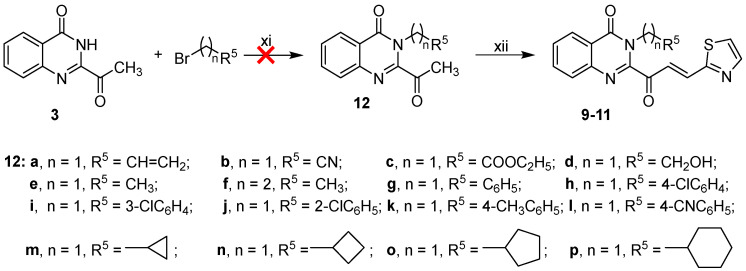
Preparation of thiazolylketenyl quinazolinones **9**–**11** with different substituents at *N*-3 position of quinazolinone. Reagents and conditions: (xi) Various halides, K_2_CO_3_, CH_3_CN, 80 °C; (xii) 2-thiazolecarboxaldehyde, piperidine, CH_3_CH_2_OH, 80 °C.

**Scheme 4:**
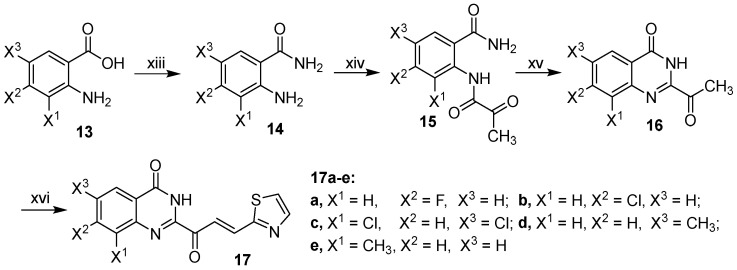
Preparation of thiazolylketenyl quinazolinones **17a**–**e** with different substituents at quinazolinone. Reagents and conditions: (xiii) *N,N′*-carbonyldiimidazole, 25% ammonia water solution, DMF, 70 °C; (xiv) pyruvic acid, 1-(3-dimethylaminopropyl)-3-ethylcarbodiimide hydrochloride, 1-hydroxybenzotriazole hydrate, DMF, r.t.; (xv) 10% NaOH aqueous solution, r.t.; (xvi) 2-thiazolecarboxaldehyde, piperidine, CH_3_CH_2_OH, 80 °C.

The preparative pipeline of target thiazolylketenyl quinazolinones and analogs was depicted in Scheme 1, Scheme 2, Scheme 3 and Scheme 4. Commercially available 2-aminobenzamide **1** and pyruvate were reacted with 1-(3-dimethylaminopropyl)-3-ethylcarbodiimide hydro-chloride and 1-hydroxybenzotriazole hydrate at room temperature to afford intermediate **2**, which was further cyclized in 10% sodium hydroxide aqueous solution to produce acetyl quinazolinone **3** with good yield [35]. Thiazolylketenyl quinazolinone **4** was developed by the aldol condensation of compound **3** with 2-thiazolecarboxaldehyde at 80 °C using piperidine as catalyst to give the product in 50.9% yield. Thiazole isomer derivatives **5a**–**b** featuring 5-position thiazoles, analogs **6a**–**c** with furans and **7a**–**b** characterized by thiophenes as well as the fused derivatives **8a**–**c** were prepared in the same process with yields of 31.0–98.4% (Scheme 1). Antibacterial activity analysis of the above derivatives found that thiazolylketenyl quinazolinone (TQ) **4** characterized by 2-position thiazole exhibited the best inhibitory activity. Therefore, it was desirable to replace the hydrogen atom at *N*-3 position of quinazolinone core with different substituents on TQ **4** to explore their effect on activity.

However, it was found that the substituted derivatives **9**–**11** on the basis of TQ **4** could not be constructed by the direct reaction of TQ **4** with different halides in acetonitrile at 80 °C, which might be associated with the base and high temperature intolerant properties of ethenone (Scheme 2). Therefore, an alternative synthetic route was attempted, this route gave the products **9**–**11** using acetyl quinazolinone **3** as the starting material by a substitution reaction with various halides followed by a condensation reaction with 2-thiazolecarboxaldehyde. Unexpectedly, none of the desired intermediates **12a**–**p** could be obtained despite having tried various combinations of conditions, such as potassium carbonate/acetonitrile, cesium carbonate/acetonitrile, cesium carbonate/DMF, NaH/DMF (Scheme 3 and Table 1). After analysis, it was suspected that the substitution reaction might be hindered by the large steric effect of the acetyl group at *C*-2 position of the quinazolinone.

To explore the effect of different polar groups on the quinazolinone ring on activity, fluoro, chloro, and methyl groups were introduced. The desired intermediates 2-aminobenzamides **14a**–**e** were prepared by the reaction of aminobenzoic acids **13a**–**e**, 25% ammonia water solution and *N*,*N′*-carbonyldiimidazole (CDI) at 70 °C in acetonitrile [36]. A series of various substituted TQs **17a**–**e** was synthesized according to the above procedure to prepare compound **4** (Scheme 4).

### 2.2. Antibacterial Activity

The prepared thiazolylketenyl quinazolinones and analogs were screened for in vitro antibacterial evaluation by the two-fold microdilution method in 96-well micro test plates using norfloxacin as a positive control, and the results are listed as the minimum inhibitory concentration values (MICs, μg/mL). As listed in Table 2, most of the prepared derivatives showed good to excellent antibacterial activity against the eleven tested bacteria. The activity results indicated that the activity of 2-position thiazole TQ **4** was superior to the isomer 5-position thiazole TQs **5a**–**b**. The MIC values of TQ **4** against MRSA and *Escherichia coli* 25922 were both 0.5 μg/mL, which was 8 times and 4 times that of norfloxacin, respectively. Moreover, TQ **4** also exhibited good antibacterial activity against MRSA ATCC 43300, *Staphylococcus aureus* ATCC 12600 and *Staphylococcus aureus* ATCC 6583 (Table S1). The poor antibacterial activity of phenylthiazole TQ **5b** was related to its extremely terrible solubility.

In order to investigate the effects of different types of aromatic heterocycles on antibacterial activity, the biological isosteres of thiazole, including thiazoles and their biological isosteres, such as thiophene and furan, as well as their fused derivatives are incorporated into the target compounds. It was found that the replacement of thiazole by furan produced furanylketenyl quinazolinone **6a** with good activity against *Klebsiella pneumoniae* with MIC value of 2 μg/mL, which was twice that of norfloxacin. The introduction of the methyl group into furanyl ring was not positive because its derivative **6b** showed slightly reduced antibacterial activity compared to **6a**. Moreover, the activity of **6c** after replacing the methyl group with the hydroxymethyl group exhibited a further decline, which suggested that the methyl and hydroxymethyl groups were detrimental to the activity of quinazolinone furans.

Upon replacement of thiazole with thiophene, thiophenylketenyl quinazolinone **7a** showed comparable activity to compound **6a**. However, contrary to the harmful effect of the methyl group on the activity of compound **6b**, the substitution of the methyl group on thiophene ring, which produced compound **7b**, significantly improved the activity. In particular, methylthiophene derivative **7b** exhibited excellent inhibition with MIC value of 0.5 μg/mL against *Pseudomonas aeruginosa* 27853, and also showed comparable activity to norfloxacin against *Staphylococcus aureus* 25923.

Further effort was moved into the substitution of thiazole ring by fused heterocycles. The incorporation of benzofuran in derivative **8a** was not beneficial in improving the antibacterial activity in comparison to furan derivative **6a** and TQ **4**. However, the fused benzothiophene in compound **8b** gave better activity than thiophene in compound **7a**. Compounds **8a**–**b** exhibited significant anti-*K. pneumoniae* inhibition, with MICs of 2 μg/mL, and **8b** also showed twice anti-*Acinetobacter baumannii* activity as norfloxacin. 

When the different substituents were introduced into the quinazolinone core of TQ **4**, the resulting TQs **17a**–**e** displayed decreased antibacterial activity in contrast to TQ **4**. However, TQs **17a**–**e** displayed excellent inhibition against *Pseudomonas aeruginosa* with MICs ranging from 2 to 4 μg/mL. Moreover, TQ **17c** substituted with two chlorine atoms exhibited twice the inhibitory activity than norfloxacin against *Enterococcus faecalis*. 

Considering the better activity of TQ **4** than the reference drug norfloxacin against MRSA, the minimum bactericidal concentration was tested through broth microdilution and agar plate colony counting assays in Table S2. The result showed that TQ **4** with MBC value of 8 μg/mL exerted bacteriostatic effect against MRSA. Altogether, TQ **4** was selected to explore further antibacterial studies, given its significant anti-MRSA activity.

### 2.3. Hemolytic Assay

**Figure 2:**
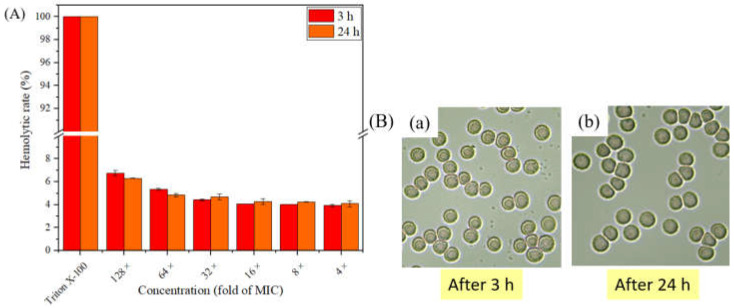
Assessment of hemolysis of compound **4**. (**A**) Hemolytic rate of compound **4** to RBCs, (**B**) microscope images of RBCs treated by compound **4** for 3 h (**a**) and 24 h (**b**).

Drug safety evaluation is an important sector in drug design, and the hemolysis test as an initial screening test is one of the important methods for the in vitro cytotoxicity test [37,38,39]. The toxicity of TQ **4** towards human red blood cells (RBCs) at different concentrations and at different times was determined by in vitro hemolysis assay. The results in Figure 2A show that the hemolytic rate of TQ **4** for RBCs is only 6.76% after human RBCs were treated for 3 h at a high concentration of 128-fold MIC value with TQ **4**. At the concentration of 32-fold MIC, the hemolytic activity of TQ **4** (4.43%) was also below the highest international standard hemolytic rate of 5%. Furthermore, after 24 h of treatment, the hemolysis rates of RBCs at different concentrations of TQ **4** were barely changed, as can also be observed in Figure 2B and Figure S1. The above in vitro hemolysis assay illustrated that TQ **4** showed no obvious blood toxicity and could be considered a safe drug.

### 2.4. Resistance Study

**Figure 3:**
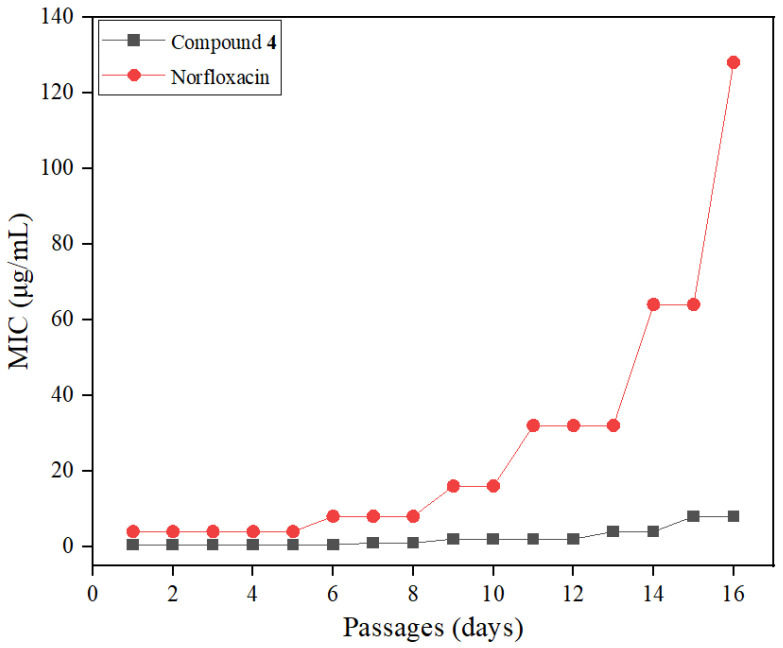
Resistance development of MRSA after treatment with compound **4** and norfloxacin.

The development of bacterial resistance leads to a reduction in the activity of clinical drugs, and the solution is often the dosage of intensification, which will not only cause adverse drug reactions in humans but also further aggravate the development of drug resistance [40,41,42]. Therefore, it is urgent to develop new drug candidates with low drug resistance tendencies. As shown in Figure 3, the susceptibility of MRSA toward TQ **4** was nearly unchanged after 16 successive cultures, while the MIC values of norfloxacin increased dramatically after the 9th passage. Especially after 16 generations of cultivation with norfloxacin, the MIC value of MRSA increased 32 times compared with the first generation. These conclusions suggested that TQ **4** with low drug resistance tendency deserves to be further developed and explored for its antibacterial mechanism.

### 2.5. ADMET Study

ADMET screening rules are an indispensable part of modern drug development [43,44,45]. They are not only an important experimental specification for estimating the safety and effectiveness of drugs in healthy populations but also an important avenue for assessing the development potential of drugs in the future. The results in Table 3 showed that TQ **4** adhered to Lipinski rules and possessed the same bioavailability score as norfloxacin, besides TQ **4** exerted high intestinal mucosal (GI) absorption while being unable to penetrate the blood-brain barrier (BBB). All these results declared that TQ **4** possessed favorable drug-likeness and pharmacokinetic properties in silico.

### 2.6. Antibiofilm Activity

**Figure 4:**
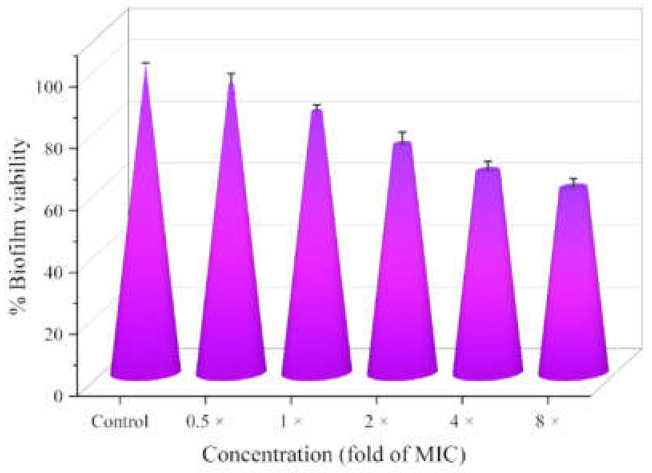
Antibiofilm viability of MRSA treated with compound **4**.

Compared to the planktonic state, bacteria with bacterial biofilms covering the surface are more drug resistant and also more resistant to attack by the host immune system [46,47,48]. TQ **4** with low inducible drug resistance was tested for its ability to inhibit bacterial biofilm formation using crystal violet staining. It can be observed in Figure 4 that biofilm formation inhibition of MRSA by TQ **4** showed a concentration-dependent increase, and the biofilm viability of MRSA decreased to 60% at 8 × MIC, indicating that TQ **4** inhibited the formation of MRSA biofilms to some extent, which might be one of the reasons for its high activity and low resistance.

### 2.7. Metabolic Activity

**Figure 5:**
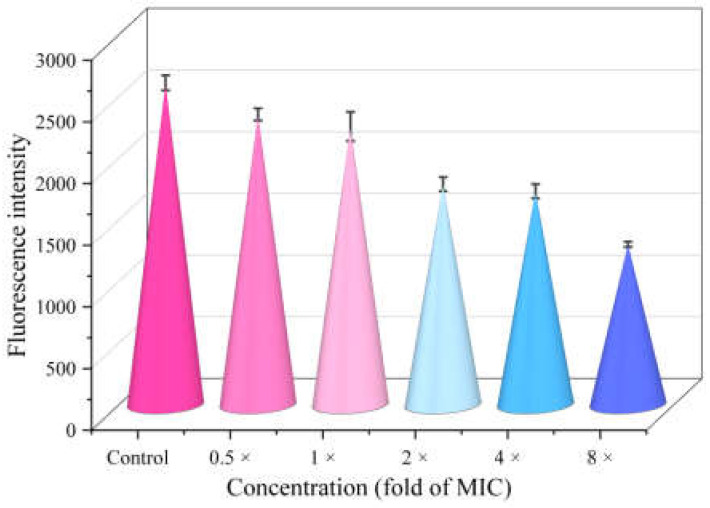
Fluorescent intensity revealing metabolic activity in MRSA after incubation with compound **4**.

During metabolism, bacteria produce energy and form metabolites that favor bacterial proliferation, such as polysaccharides, proteins, fats, nucleic acids, the cell wall, and various coenzymes, so the metabolic activity represents the healthy viability of bacteria [49,50]. The metabolic function of MRSA was measured by employing the redox indicator Alamar Blue, which commonly served as a redox indicator to reflect bacterial metabolic activities. According to the results shown in Figure 5, TQ **4** effectively affected the metabolic activity of MRSA and further produced inhibition with increasing concentrations, which might also be the reason for excellent anti-MRSA capability of TQ **4**.

### 2.8. Membrane Damage Assay

Bacterial cell membrane, one of the non-deletable important components of bacteria, played an irreplaceable role in the morphological maintenance and energy metabolism of bacteria. Furthermore, cell membranes not only protect the bacteria from mechanical damage and chemical attack but also participate in many physiological functions as channels for the entry and exit of substances, such as the absorption, distribution, excretion, and delivery of substances [51,52,53]. Therefore, disrupting the integrity of cell membrane enables the morphological breakage or physiological function disturbance of bacteria to cause the death of bacteria.

#### 2.8.1. Membrane Depolarization Assay

**Figure 6:**
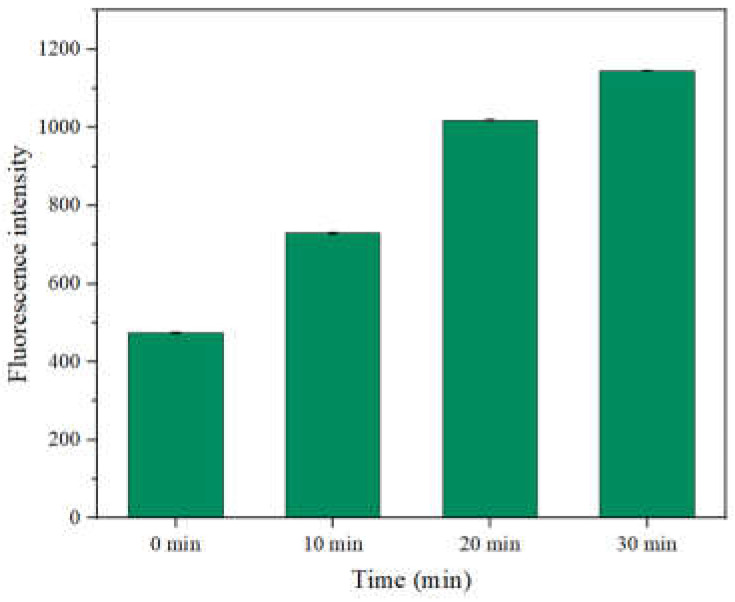
Detection of membrane depolarization of MRSA treated by compound **4**.

Membrane potential as a momentous free energy source for bacteria is provided with noteworthy physiological implications for stress regulation and signal transmission [54,55]. Dye 3,3′-dipropylthiadicarbocyanine iodide (diSC35) as a potentiometric probe was used to assess the depolarizing ability of TQ **4** on the cell membrane of MRSA [56,57]. The results in Figure 6 showed that the fluorescence intensity gradually enhanced with the increase in treatment time, which indicated that TQ **4** caused a rapid depolarization of cell membrane potential.

#### 2.8.2. Study of Inner Membrane Permeabilization

**Figure 7:**
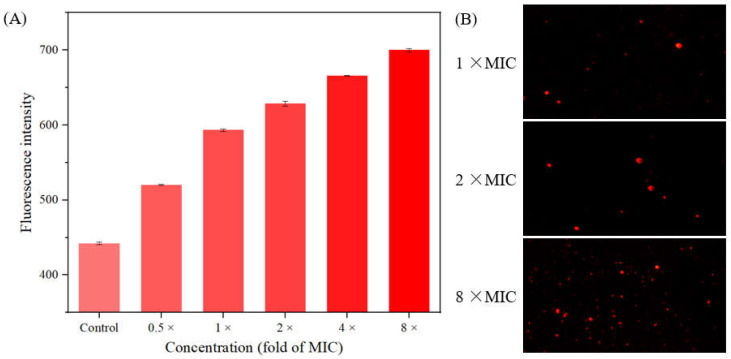
Damage to the inner membrane of MRSA cells by compound **4** under fluorescence (**A**) and microscopy (**B**).

Propidium iodide (PI) could cross the cell membrane of dead bacteria to reach the nucleus, where it intercalated with the cell’s DNA to produce red fluorescence [58,59]. To further explore the effect of TQ **4** on bacterial membrane permeability, the PI accumulation of MRSA after treatment with TQ **4** at different concentrations was determined. As shown in Figure 7A,B, the intracellular PI content after treatment increased significantly in a concentration-dependent manner, indicating that TQ **4** promoted the inner permeability of MRSA.

Both membrane depolarization and inner membrane permeability experiments demonstrated that TQ **4** was able to disrupt the integrity of the membrane to affect the normal physiological function of cells or cause membrane damage.

### 2.9. The Leakage of Intracellular Protein

**Figure 8:**
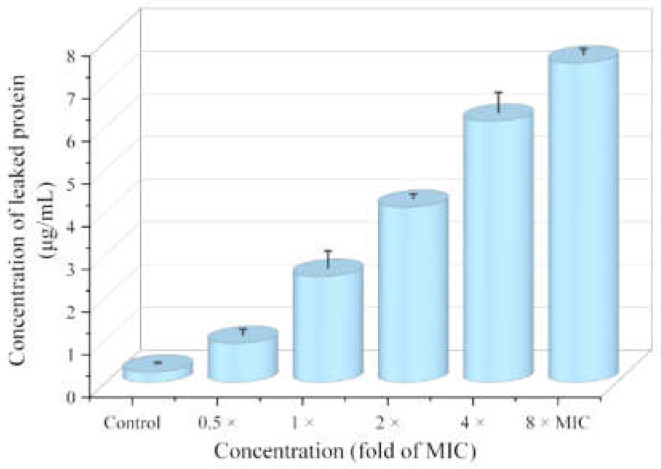
Leakage of intracellular protein in the presence of different concentrations of compound **4**.

The destruction of cell membrane integrity will lead to cell lysis and the release of cell contents [60,61,62]. The extent of MRSA protein leakage determined by the Bradford assay was used as additional evidence of membrane damage, and the results are displayed in Figure 8. The degree of protein leakage was positively correlated with the concentration of TQ **4**, and the MRSA protein leakage concentration at 8 × MIC was 8 times that at 0.5 × MIC. The above results illustrate that TQ **4** caused leakage of intracellular proteins, possibly by disrupting the membrane integrity.

### 2.10. Intracellular ROS Accumulation

**Figure 9:**
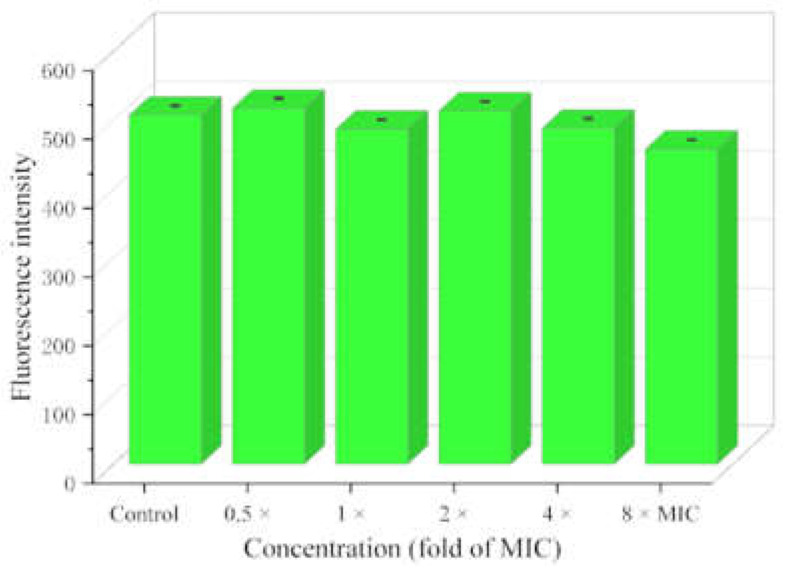
Reduction of ROS after exposure to compound **4**.

Reactive oxygen species (ROS), as natural byproducts of normal metabolism, play an important role in cell signaling and homeostasis [63,64]. The level of ROS increases dramatically when bacteria receive external stress, resulting in severe damage to the cellular structure, which is known as oxidative stress [65,66,67]. Given that TQ **4** was able to reduce MRSA metabolic activity and disrupt membrane integrity, the reactive oxygen species accumulation of MRSA after treatment with TQ **4** was determined using DCFH-DA as an indicator. However, the fluorescence intensity showed negligible changes at different concentrations of TQ **4** in Figure 9, manifesting that TQ **4** could not affect the bacterial structure and metabolic activity by provoking the production of intracellular ROS of MRSA.

### 2.11. Interaction between Compound ***4*** and DNA

Deoxyribonucleic acid (DNA), with the function of directing the synthesis of proteins and controlling metabolic processes, has been recognized as an effective drug target in the design and development of novel efficient antibacterial drugs [68,69,70]. Considering that TQ **4** possessed a large conjugated system that possibility of intercalating into DNA and the ability to easily form hydrogen bonds and π-π interactions with DNA, thereby impeding DNA replication, the interaction between TQ **4** and calf thymus DNA was studied by UV–vis and fluorescence spectroscopy [71,72].

#### 2.11.1. DNA Binding Study

**Figure 10:**
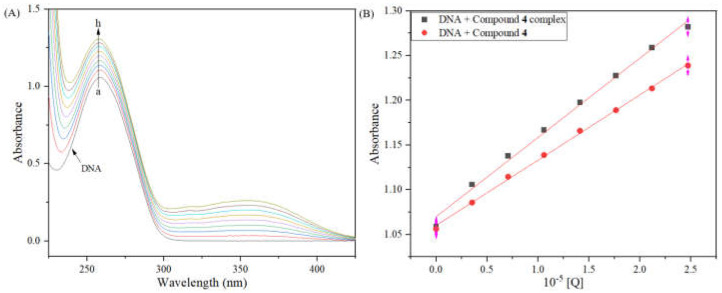
(**A**) UV absorption spectra of calf thymus DNA with compound **4** (pH = 7.4, T = 295 K). c (DNA) = 1.56 × 10^−4^ mol/L and c (compound **4**) = 0–2.67× 10^−5^ mol/L; (**B**) absorption comparison at 260 nm between the compound **4**–DNA complex and the sum of compound **4** and DNA.

As shown in Figure 10, the maximum absorption peak of DNA at 260 nm raised with the amount of TQ **4** increased, and the increment of absorption peak was proportional to the increment of concentration. Moreover, the absorption of **4**–DNA complex was higher than the sum of TQ **4** and DNA, which illustrated that TQ **4** might have caused denaturation of DNA and partial disintegration of double helix. This phenomenon suggested a strong interaction occurred between TQ **4** and DNA.

#### 2.11.2. Competitive Binding Study

**Figure 11:**
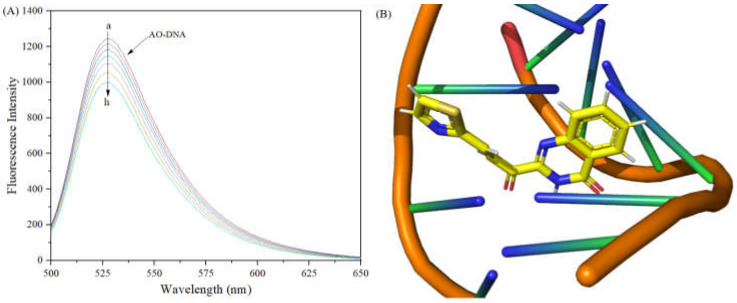
(**A**) Competitive reaction between AO–DNA system and compound **4**. c (DNA) = 1.56 × 10^−4^ mol/L and c (**4**) = 0–2.67 × 10^−5^ mol/L. (**B**) docking diagram of molecule **4** (yellow) and DNA (orange) (PDB: 454D).

Aiming to illuminate the action pattern between the TQ **4** and DNA, further research was conducted using a fluorescence probe of acridine orange (AO), which was a nucleic acid binding dye that could be embedded in DNA to emit fluorescence [73]. The maximum absorption of AO–DNA complex gradually decreased as the concentration of TQ **4** increased, indicating that TQ **4** can effectively insert into DNA by replacing AO in the AO–DNA complex, preventing bacterial DNA replication, and thereby inhibiting the growth of MRSA. Furthermore, it can be clearly observed in Figure 11B that the TQ **4** intercalated into DNA base pairs, which further provided proof for insertion of TQ **4** into DNA.

### 2.12. Allosteric Modulation of Compound ***4*** with PBP2a

#### 2.12.1. Contents of PBP2a and Drug Combination

Previous studies on PBP2a have confirmed that quinazolinone derivatives can open the active site as an allosteric apparatus of PBP2a, prompting the active site to interact with the *β*-Lactam antimicrobials, eventually resulting in impaired cell wall biosynthesis and a bactericidal effect [34]. In view of excellent anti-MRSA activity and low drug resistance of TQ **4**, the potential of TQ **4** as an allosteric to PBP2a was explored [74].

The effect of TQ **4** alone and combined with *β*-lactam drugs on PBP2a contents was investigated. The results in Table 4 showed that the expression of PBP2a treated with a combination of TQ **4** and cefdinir was lower than that of either treatment alone, preliminary indicating that TQ **4** could exert synergistic effects with cefdinir to inhibit the expression of PBP2a. Furthermore, the drug combination results revealed that the combination of TQ **4** with cefdinir significantly enhanced the antibacterial potency of cefdinir against MRSA and the fractional inhibitory concentration index (FICI) = 0.375, indicating a synergistic effect.

#### 2.12.2. Allosteric Site Binding Affinity of PBP2a

**Figure 12:**
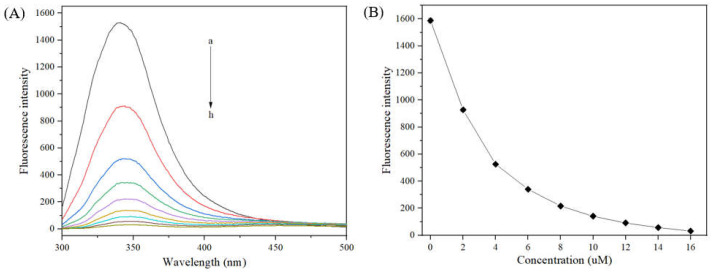
Binding of derivative **4** to PBP2a allosteric site (λ_ex_ = 280 nm). (**A**) Emission spectra of acylated PBP2a intrinsic fluorescence with **4** at different concentrations. (**B**) The change in the maximum fluorescence intensity.

To further validate the interaction of TQ **4** with the active site of PBP2a, the binding affinity of TQ **4** and the PBP2a allosteric site was determined by intrinsic fluorescence assays. Using the fluorophotometer, PBP2a (500 nM) was incubated in buffer at room temperature for 10 min. Upon excitation at 280 nm, the emission spectra of protein alone were taken. TQ **4** was titrated into the reaction, and the emission spectra were measured. The fluorescence intensity of PBP2a allosteric sites gradually decreased as the content of TQ **4** increased in Figure 12, which provided categorical evidence for the binding of **4** to PBP2a allosteric sites.

The above results indicated that TQ **4** significantly enhanced the anti-MRSA activity of cefdinir by inducing PBP2a allosteric regulation of MRSA and triggering the opening of the active site, enabling cefdinir to bind to the active site to inhibit the expression of PBP2a. 

#### 2.12.3. Molecular Docking

**Figure 13:**
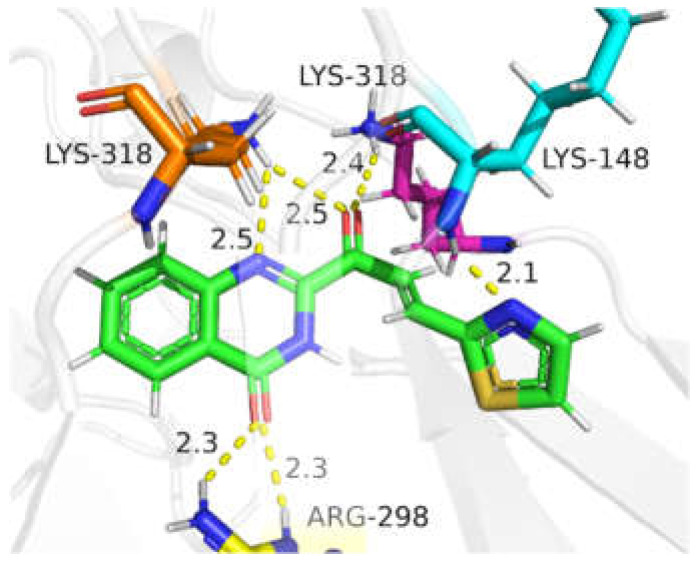
In silico docking of compound **4** with PBP2a (PDB code 4CJN). Color-codes are as follows: green, compound **4**; blue, residue LYS-148; orange, residue LYS-318; purple, residue, LYS-318; yellow, residue ARG-298; yellow dashed lines: hydrogen bonds).

Docking is an effective and reliable method to simulate the possible binding mode of molecules and proteins [75,76]. To probe the binding mode of TQ **4** to the active site of PBP2a, the binding in the active site and allosteric site of TQ **4** with PBP2a were analyzed by computational simulation. As displayed in Figure 13, the carbonyl group at *C*-4 position and nitrogen atom at *N*-1 position of quinazolinone could form hydrogen bonds with residues ARG-298 and LYS-318 with a space distance of 2.5 Å, respectively. Moreover, the carbonyl group of ethenone was able to interact with two different residues LYS-318, via hydrogen bonds. Meanwhile, the nitrogen atom of thiazole also participated in hydrogen bond with residue LYS-148 with a space distance of 2.1 Å. These results indicated that TQ **4** with excellent binding ability to PBP2a was able to form multiple hydrogen bonding interactions with the active site of PBP2a.

### 2.13. Summary of Anti-MRSA Behavior of Thiazolylketenyl Quinazolinones

**Figure 14:**
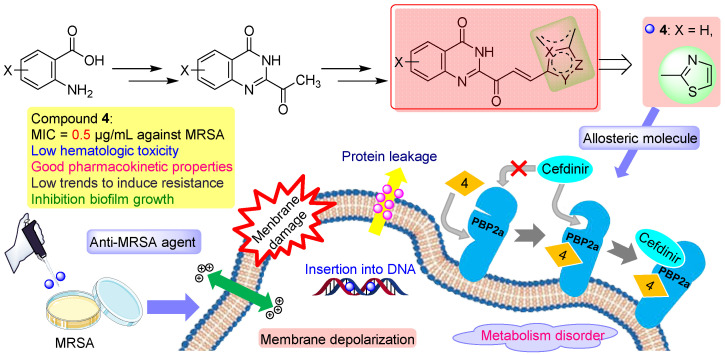
The summary of anti-MRSA behavior thiazolylketenyl quinazolinone **4** as anti-MRSA agents and allosteric modulator for PBP2a.

Preliminary mechanistic exploration indicated that TQ **4** was unable to generate oxidative stress, but it was able to hamper the replication of DNA by intercalating DNA, leading to the death of bacteria. Furthermore, TQ **4** disrupted membrane integrity, thus leading the leakage of protein. Surprisingly, TQ **4** was found to be able to enhance the anti-MRSA activity of cefdinir upon binding to the allosteric site of PBP2a and exciting conformational switch, to promote the combination of active site and cefdinir, thus reducing expression of PBP2a. The summary of anti-MRSA behavior of thiazolylketenyl quinazolinone **4** is shown in Figure 14.

## 3. Materials and Methods

### 3.1. Instruments and Chemicals

All the reagents and solvents were purchased commercially and could be used without purification. The reagents and solvents used in the experiment, such as potassium carbonate, cesium carbonate, sodium hydride, piperidine, acetonitrile, *N,N*-dimethylformamide, petroleum ether, ethyl acetate, anhydrous ethanol and anhydrous sodium sulfate were prepared by Chengdu Kelong Chemical Co., Ltd. (Chengdu, China) and Chongqing Wansheng Chuandong Chemical Co., Ltd. (Chongqing, China). Chemicals such as 2-aminobenzamide, pyruvic acid, 1-(3-dimethylaminopropyl)-3-ethylcarbodiimide hydrochloride, and 1-hydroxybenzotriazole hydrate were purchased from Shanghai Titan Technology Co., Ltd. (Shanghai, China) or Shanghai Bide Pharmaceutical Technology Co., Ltd. (Shanghai, China). The melting points were gauged on the X-6 melting point instrument. The NMR spectra, including ^1^H NMR and ^13^C NMR of target compounds, were recorded on the Bruker AVANCE III 600 MHz spectrometer. The high-resolution mass spectra (HRMS) of molecules were measured by Bruker Impact II (Waters Micromass, Milford, MA, USA) using an ESI resource. Purity measurements of thiazolylketenyl quinazolinones and analogs were surveyed by PM 1000 (HITACHI primaide) high-performance liquid chromatography (HPLC) with two mobile phases (A: acetonitrile, B: water) at 1 mL/min flow. All fluorescent spectra were recorded on F-7000 spectrofluorimeter (Hitachi, Tokyo, Japan), and UV spectra were obtained through TU-2450 spectrophotometer (Puxi Analytic Instrument Ltd. of Beijing, China). Microscopic observations were made with a positive fluorescence microscope (LW300LFT, Cewei, China). The OD values were measured by a full-wavelength microplate reader (Thermo Fisher Scientific, Waltham, MA, USA).

### 3.2. Synthesis of Intermediates and Thiazolylketenyl Quinazolinones and Analogs

#### 3.2.1. Synthesis of Intermediates **2**–**3** and **15**–**16**

Intermediates **2**–**3** and **15**–**16** were obtained through the reported methods described in reference [35].

#### 3.2.2. Synthesis of Intermediate **14**

Intermediate **14** was obtained through the reported methods described in reference [36].

#### 3.2.3. Synthesis of (E)-2-(3-(thiazol-2-yl)acryloyl)quinazolin-4(3H)-one (**4**)

A mixture of compound **3** (50 mg, 0.27 mmol), thiazole-2-carbaldehyde (30 mg, 0.27 mmol), and piperidine (0.2 mL) in ethanol (5 mL) was stirred at 80 °C for 1 h. After the reaction was completed, the solvent was removed. The crude product was purified by silica gel column chromatography (300–400 mesh) (eluent, dichloromethane/methanol = 30~10/1, *v*/*v*) and recrystallized in ethanol to afford compound **4** as yellow solid (38 mg). Yield: 50.9%, mp > 300 °C. ^1^H NMR (600 MHz, DMSO-*d*_6_) δ 12.46 (s, 1H, quinazolinone-N*H*), 8.30 (d, *J* = 15.9 Hz, 1H, CH=C*H*-thiazole), 8.22 (d, *J* = 7.8 Hz, 1H, quinazolinone-5-*H*), 8.13 (d, 1H, *J* = 2.9 Hz, thiazole-4-*H*), 8.07 (d, 1H, *J* = 3.0 Hz, thiazole-5-*H*), 8.01 (d, *J* = 15.9 Hz, 1H, C*H*=CH-thiazole), 8.00–7.92 (m, 2H, quinazolinone-7-*H*, quinazolinone-8-*H*), 7.69 (t, 1H, *J* = 7.1 Hz, quinazolinone-6-*H*). ^13^C NMR (151 MHz, DMSO-*d*_6_) δ 183.34, 163.14, 161.32, 148.52, 147.61, 145.89, 136.01, 135.32, 129.60, 129.22, 126.68, 125.34, 123.78, 123.52. HRMS (ESI) calcd. for C_14_H_9_N_2_O_2_S [M + H]^+^, 248.0488; found, 248.0488.

#### 3.2.4. Synthesis of (E)-2-(3-(thiazol-5-yl)acryloyl)quinazolin-4(3H)-one (**5a**)

Compound **5a** was prepared according to the procedure described for compound **4**, starting from compound **3** (50 mg, 0.27 mmol) and thiazole-5-carbaldehyde (30 mg, 0.27 mmol). The target compound **5a** was obtained as yellow solid (44 mg). Yield: 58.7%, mp 187–189 °C. ^1^H NMR (600 MHz, DMSO-*d*_6_) δ 12.41 (s, 1H, quinazolinone-N*H*), 9.33 (s, 1H, thiazole-2-*H*), 8.49 (s, 1H, thiazole-4-*H*), 8.27–8.15 (m, 2H, quinazolinone-5-*H*, CH=C*H*-thiazole), 7.94 (m, 2H, quinazolinone-7-*H*, quinazolinone-8-*H*), 7.82 (d, *J* = 15.8 Hz, 1H, C*H*=CH-thiazole), 7.69 (t, *J* = 7.1 Hz, 1H, quinazolinone-6-*H*). ^13^C NMR (151 MHz, DMSO-*d*_6_) δ 183.05, 161.36, 158.27, 149.54, 148.56, 147.61, 135.66, 135.29, 129.50, 129.18, 126.65, 126.02, 123.68, 122.17. HRMS (ESI) calcd. for C_14_H_9_N_2_O_2_S [M + H]^+^, 248.0488; found, 248.0494.

#### 3.2.5. Synthesis of (E)-2-(3-(2-phenylthiazol-5-yl)acryloyl)quinazolin-4(3H)-one (**5b**)

Compound **5b** was prepared according to the procedure described for compound **4**, starting from compound **3** (50 mg, 0.27 mmol) and 2-phenylthiazole-5-carbaldehyde (51 mg, 0.27 mmol). The target compound **5b** was obtained as yellow solid (94 mg). Yield: 98.4%, mp 216–218 °C. ^1^H NMR (600 MHz, DMSO-*d*_6_) δ 12.41 (s, 1H, quinazolinone-N*H*), 8.48 (s, 1H, thiazole-4-*H*), 8.22 (d, *J* = 7.8 Hz, 1H, quinazolinone-5-*H*), 8.18 (d, *J* = 15.8 Hz, 1H, CH=C*H*-thiazole), 8.07–8.02 (m, 2H, Ph-2-*H*, Ph-6-*H*), 7.95–7.94 (m, 2H, quinazolinone-8-*H*, quinazolinone-7-*H*), 7.82 (d, *J* = 15.8 Hz, 1H, C*H*=CH-thiazole), 7.69 (m, 1H, quinazolinone-6-*H*), 7.59–7.55 (m, 3H, Ph-3-*H*, Ph-4-*H*, Ph-5-*H*). HRMS (ESI) calcd. for C_20_H_13_N_3_O_2_S [M+Na]^+^, 360.0801; found, 360.0801.

#### 3.2.6. Synthesis of (E)-2-(3-(furan-2-yl)acryloyl)quinazolin-4(3H)-one (**6a**)

Compound **6a** was prepared according to the procedure described for compound **4**, starting from compound **3** (50 mg, 0.27 mmol) and furan-2-carbaldehyde (26 mg, 0.27 mmol). The target compound **6a** was obtained as yellow solid (54 mg). Yield: 76.3%, mp 216–218 °C. ^1^H NMR (600 MHz, DMSO-*d*_6_) δ 12.31 (s, 1H, quinazolinone-N*H*), 8.22 (d, *J* = 7.9 Hz, 1H, quinazolinone-5-*H*), 8.01 (s, 1H, furan-5-*H*), 7.96–7.91 (m, 2H, quinazolinone-7-*H*, quinazolinone-8-*H*), 7.86 (d, *J* = 15.9 Hz, 1H, CH=C*H*-furan), 7.74 (d, *J* = 15.9 Hz, 1H, C*H*=CH-furan), 7.68 (t, *J* = 7.8 Hz, 1H, quinazolinone-6-*H*), 7.20 (d, *J* = 3.4 Hz, 1H, furan-3-*H*), 6.75 (dd, *J* = 3.5, 1.8 Hz, 1H, furan-4-*H*). ^13^C NMR (151 MHz, DMSO-*d*_6_) δ 183.11, 161.29, 151.47, 148.66, 147.70, 135.25, 132.04, 129.38, 129.13, 126.62, 123.65, 119.59, 116.85, 113.97. HRMS (ESI) calcd. for C_15_H_10_N_2_O_3_ [M + H]^+^, 267.0764; found, 267.0765.

#### 3.2.7. Synthesis of (E)-2-(3-(5-methylfuran-2-yl)acryloyl)quinazolin-4(3H)-one (**6b**)

Compound **6b** was prepared according to the procedure described for compound **4**, starting from compound **3** (50 mg, 0.27 mmol) and 5-methylfuran-2-carbaldehyde (29 mg, 0.27 mmol). The target compound **6b** was obtained as yellow solid (57 mg). Yield: 76.5%, mp 195–197 °C. ^1^H NMR (600 MHz, DMSO-*d*_6_) δ 12.27 (s, 1H, quinazolinone-N*H*), 8.21 (d, *J* = 7.8 Hz, 1H, quinazolinone-5-*H*), 7.95–7.91 (m, 2H, quinazolinone-8-*H*, quinazolinone-7-*H*), 7.74 (d, *J* = 15.7 Hz, 1H, CH=C*H*-furan), 7.71–7.63 (m, 2H, C*H*=CH-furan, quinazolinone-6-*H*), 7.12 (d, *J* = 2.9 Hz, 1H, furan-3-*H*), 6.41 (d, *J* = 2.5 Hz, 1H, furan-4-*H*), 2.44 (s, 3H, furan-C*H*_3_). ^13^C NMR (101 MHz, DMSO-*d*_6_) δ 182.89, 161.35, 157.97, 150.27, 148.74, 147.72, 135.29, 132.06, 129.35, 129.07, 126.62, 121.95, 114.92, 110.95, 14.27. HRMS (ESI) calcd. for C_16_H_12_N_2_O_3_ [M + H]^+^, 281.0921; found, 281.0918.

#### 3.2.8. Synthesis of (E)-2-(3-(5-(hydroxymethyl)furan-2-yl)acryloyl)quinazolin-4(3H)-one (**6c**)

Compound **6c** was prepared according to the procedure described for compound **4**, starting from compound **3** (50 mg, 0.27 mmol) and 5-(hydroxymethyl)furan-2-carbaldehyde (34 mg, 0.27 mmol). The target compound **6c** was obtained as yellow solid (61 mg). Yield: 77.5%, mp 220–222 °C. ^1^H NMR (600 MHz, DMSO-*d*_6_) δ 12.30 (s, 1H, quinazolinone-N*H*), 8.21 (d, *J* = 7.8 Hz, 1H, quinazolinone-5-*H*), 7.94 (m, 2H, quinazolinone-8-*H*, quinazolinone-7-*H*), 7.80 (d, *J* = 15.8 Hz, 1H, CH=C*H*-furan), 7.74–7.65 (m, 2H, C*H*=CH-furan, quinazolinone-6-*H*), 7.15 (d, *J* = 2.9 Hz, 1H, furan-3-*H*), 6.57 (d, *J* = 2.9 Hz, 1H, furan-4-*H*), 5.54 (s, 1H, CH_2_-O*H*), 4.54 (s, 2H, C*H*_2_-OH). ^13^C NMR (101 MHz, DMSO-*d*_6_) δ 182.97, 161.32, 160.72, 150.79, 148.70, 147.73, 135.27, 132.12, 129.37, 129.09, 126.62, 123.60, 121.08, 115.90, 111.07, 56.37. HRMS (ESI) calcd. for C_16_H_12_N_2_O_4_ [M + H]^+^, 297.0870; found, 297.0868.

#### 3.2.9. Synthesis of (E)-2-(3-(thiophen-2-yl)acryloyl)quinazolin-4(3H)-one (**7a**)

Compound **7a** was prepared according to the procedure described for compound **4**, starting from compound **3** (50 mg, 0.27 mmol) and thiophene-2-carbaldehyde (30 mg, 0.64 mmol). The target compound **7a** was obtained as yellow solid (59 mg). Yield: 78.7%, mp 183–185 °C. ^1^H NMR (600 MHz, DMSO-*d*_6_) δ 12.34 (s, 1H, quinazolinone-N*H*), 8.22 (d, *J* = 7.8 Hz, 1H, quinazolinone-5-*H*), 8.12 (d, *J* = 15.8 Hz, 1H, CH=C*H*-thiophene), 7.94 (m, 2H, quinazolinone-7-*H*, quinazolinone-8-*H*), 7.89 (d, *J* = 4.9 Hz, 1H, thiophene-5-*H*), 7.80 (d, *J* = 15.8 Hz, 1H, C*H*=CH-thiophene), 7.75 (d, *J* = 3.3 Hz, 1H, thiophene-3-*H*), 7.68 (t, *J* = 6.2 Hz, 1H, quinazolinone-6-*H*), 7.25 (t, *J* = 4.2 Hz, 1H, thiophene-4-*H*). ^13^C NMR (151 MHz, DMSO-*d*_6_) δ 183.06, 161.30, 148.70, 147.69, 140.06, 138.92, 135.25, 135.01, 132.10, 129.59, 129.38, 129.14, 126.64, 123.66, 118.42. HRMS (ESI) calcd. for C_15_H_10_N_2_O_2_S [M + H]^+^, 283.0536; found, 283.0535.

#### 3.2.10. Synthesis of (E)-2-(3-(3-methylthiophen-2-yl)acryloyl)quinazolin-4(3H)-one (**7b**)

Compound **7b** was prepared according to the procedure described for compound **4**, starting from compound **3** (50 mg, 0.27 mmol) and 3-methylthiophene-2-carbaldehyde (34 mg, 0.27 mmol). The target compound **7b** was obtained as yellow solid (70 mg). Yield: 88.9%, mp 209–211°C. ^1^H NMR (600 MHz, DMSO-*d*_6_) δ 12.33 (s, 1H, quinazolinone-N*H*), 8.21 (d, *J* = 7.8 Hz, 1H, quinazolinone-5-*H*), 8.09 (d, *J* = 15.6 Hz, 1H, CH=C*H*-thiophene), 7.93 (m, 2H, quinazolinone-8-*H*, quinazolinone-7-*H*), 7.79 (d, *J* = 4.9 Hz, 1H, thiophene-5-*H*), 7.72 (d, *J* = 15.6 Hz, 1H, C*H*=CH-thiophene), 7.68 (m, 1H, quinazolinone-6-*H*), 7.10 (d, *J* = 5.0 Hz, 1H, thiophene-4-*H*), 2.42 (s, 3H, thiophene-C*H*_3_). ^13^C NMR (101 MHz, DMSO-*d*_6_) δ 182.75, 161.37, 148.69, 147.67, 145.19, 136.88, 135.25, 134.17, 132.56, 130.73, 129.37, 129.14, 126.62, 123.61, 117.34, 14.48. HRMS (ESI) calcd. for C_16_H_12_N_2_O_2_S [M + H]^+^, 297.0692; found, 297.0691.

#### 3.2.11. Synthesis of (E)-2-(3-(benzofuran-3-yl)acryloyl)quinazolin-4(3H)-one (**8a**)

Compound **8a** was prepared according to the procedure described for compound **4**, starting from compound **3** (50 mg, 0.27 mmol) and benzofuran-3-carbaldehyde (37 mg, 0.27 mmol). The target compound **8a** was obtained as yellow solid (32 mg). Yield: 38.1%, mp 205–207 °C. ^1^H NMR (600 MHz, DMSO-*d*_6_) δ 12.42 (s, 1H, quinazolinone-N*H*), 8.78 (s, 1H, benzofuran-2-*H*), 8.26–8.18 (m, 2H, CH=C*H*-benzofuran, quinazolinone-5-*H*), 8.13–8.07 (m, 2H, C*H*=CH-benzofuran, benzofuran-4-*H*), 8.00 (d, *J* = 8.0 Hz, 1H, benzofuran-7-*H*), 7.96 (t, *J* = 7.4 Hz, 1H, quinazolinone-7-*H*), 7.76 (d, *J* = 7.7 Hz, 1H, quinazolinone-8-*H*), 7.70 (t, *J* = 7.4 Hz, 1H, quinazolinone-6-*H*), 7.54–7.49 (m, 2H, benzothiophene-6-*H*, benzothiophene-5-*H*). ^13^C NMR (151 MHz, DMSO-*d*_6_) δ 183.60, 161.37, 156.11, 152.43, 148.78, 147.74, 136.34, 135.25, 129.43, 129.29, 126.64, 126.33, 124.92, 124.62, 123.68, 121.49, 120.03, 118.49, 112.67. HRMS (ESI) calcd. for C_19_H_12_N_2_O_3_ [M+Na]^+^, 339.0740; found, 339.0744.

#### 3.2.12. Synthesis of (E)-2-(3-(benzo[b]thiophen-3-yl)acryloyl)quinazolin-4(3H)-one (**8b**)

Compound **8b** was prepared according to the procedure described for compound **4**, starting from compound **3** (50 mg, 0.27 mmol) and benzo[b]thiophene-3-carbaldehyde (43 mg, 0.27 mmol). The target compound **8b** was obtained as yellow solid (54 mg). Yield: 61.2%, mp 196–198 °C. ^1^H NMR (600 MHz, DMSO-*d*_6_) δ 12.42 (s, 1H, quinazolinone-N*H*), 8.71 (s, 1H, benzothiophene-2-*H*), 8.23 (m, 4H, CH=C*H*-benzothiophene, quinazolinone-5-*H*, C*H*=CH-benzothiophene, benzothiophene-4-*H*), 8.13 (d, *J* = 8.0 Hz, 1H, benzothiophene-7-*H*), 7.95 (m, 2H, quinazolinone-7-*H*, quinazolinone-8-*H*), 7.69 (m, 1H, quinazolinone-6-*H*), 7.59 (t, *J* = 7.5 Hz, 1H, benzothiophene-6-*H*), 7.52 (t, *J* = 7.5 Hz, 1H, benzothiophene-5-*H*). ^13^C NMR (151 MHz, DMSO-*d*_6_) δ 183.77, 161.43, 148.88, 147.71, 140.55, 137.63, 137.30, 135.28, 133.53, 131.88, 129.40, 129.10, 126.66, 125.96, 125.83, 123.97, 123.65, 122.55, 120.43. HRMS (ESI) calcd. for C_19_H_12_N_2_O_2_S [M + H]^+^, 333.0692; found, 333.0693.

#### 3.2.13. Synthesis of (E)-2-(3-(1H-indol-3-yl)acryloyl)quinazolin-4(3H)-one (**8c**)

Compound **8c** was prepared according to the procedure described for compound **4**, starting from compound **3** (50 mg, 0.27 mmol) and 1*H*-indole-3-carbaldehyde (39 mg, 0.27 mmol). The target compound **8c** was obtained as yellow solid (26 mg). Yield: 31.0%, mp 234–236 °C. ^1^H NMR (600 MHz, DMSO-*d*_6_) δ 12.16 (s, 2H, indole-N*H*, quinazolinone-N*H*), 8.28–8.21 (m, 3H, quinazolinone-5-*H*, CH=C*H*-indole, indole-2-*H*), 8.05–8.00 (m, 2H, quinazolinone-7-*H*, C*H*=CH-indole), 7.98 (d, *J* = 8.0 Hz, 1H, indole-4-*H*), 7.94 (m, 1H, quinazolinone-8-*H*), 7.67 (t, *J* = 7.4 Hz, 1H, quinazolinone-6-*H*), 7.55 (d, *J* = 7.8 Hz, 1H, indole-7-*H*), 7.32 (m, 2H, indole-5-*H*, indole-6-*H*). ^13^C NMR (151 MHz, DMSO-*d*_6_) δ 182.87, 161.34, 149.42, 147.99, 141.67, 138.29, 135.65, 135.16, 129.13, 129.02, 126.61, 125.58, 123.61, 123.52, 122.23, 120.59, 113.71, 113.42, 113.31. HRMS (ESI) calcd. for C_19_H_13_N_3_O_2_ [M + H]^+^, 316.1081; found, 316.1074.

#### 3.2.14. Synthesis of (E)-7-fluoro-2-(3-(thiazol-2-yl)acryloyl)quinazolin-4(3H)-one (**17a**)

Compound **17a** was prepared according to the procedure described for compound **4**, starting from compound **16a** (50 mg, 0.24 mmol) and thiazole-2-carbaldehyde (27 mg, 0.24 mmol). The target compound **17a** was obtained as yellow solid (34 mg). Yield: 46.7%, mp 213–215 °C. ^1^H NMR (600 MHz, DMSO-*d*_6_) δ 12.58 (s, 1H, quinazolinone-N*H*), 8.27 (d, *J* = 10.1 Hz,1H, thiazole-4-*H*), 8.25 (d, *J* = 11.1 Hz, 1H, quinazolinone-5-*H*), 8.13 (d, *J* = 3.0 Hz, 1H, CH=C*H*-thiazole), 8.08 (d, *J* = 3.0 Hz, 1H, C*H*=CH- thiazole), 8.00 (d, *J* = 15.9 Hz, 1H, quinazolinone-8-H), 7.79 (d, *J* = 7.6 Hz, 1H, thiazole-5-*H*), 7.54 (t, *J* = 7.5 Hz, 1H, thiazole-6-*H*). ^13^C NMR (151 MHz, DMSO-*d*_6_) δ 183.27, 163.07, 160.68, 149.85, 149.64, 145.90, 136.28, 129.74, 129.66, 125.40, 123.48, 120.80, 117.95, 114.44. HRMS (ESI) calcd. for C_14_H_8_FN_3_O_2_S [M + H]^+^, 302.0394; found, 302.0393.

#### 3.2.15. Synthesis of (E)-7-chloro-2-(3-(thiazol-2-yl)acryloyl)quinazolin-4(3H)-one (**17b**)

Compound **17b** was prepared according to the procedure described for compound **4**, starting from compound **16b** (50 mg, 0.22 mmol) and thiazole-2-carbaldehyde (26 mg, 0.22 mmol). The target compound **17b** was obtained as yellow solid (26 mg). Yield: 36.4%, mp 205–207 °C. ^1^H NMR (600 MHz, DMSO-*d*_6_) δ 12.64 (s, 1H, quinazolinone-N*H*), 8.24 (d, *J* = 15.9 Hz, 1H, thiazole-4-*H*), 8.20 (d, *J* = 8.5 Hz, 1H, quinazolinone-5-*H*), 8.13 (s, 1H, CH=C*H*- thiazole), 8.08 (s, 1H, C*H*=CH- thiazole), 8.06 (s, 1H, quinazolinone-8-H), 8.00 (d, *J* = 15.9 Hz, 1H, thiazole-5-*H*), 7.71 (d, *J* = 8.5 Hz, 1H, thiazole-6-*H*). ^13^C NMR (151 MHz, DMSO-*d*_6_) δ 183.26, 163.10, 161.33, 148.86, 147.38, 145.91, 139.90, 136.35, 134.99, 129.78, 128.66, 127.50, 125.41, 123.52. HRMS (ESI) calcd. for C_14_H_8_ClN_3_O_2_S [M + H]^+^, 318.0099; found, 318.0097.

#### 3.2.16. Synthesis of (E)-6,8-dichloro-2-(3-(thiazol-2-yl)acryloyl)quinazolin-4(3H)-one (**17c**)

Compound **17c** was prepared according to the procedure described for compound **4**, starting from compound **16c** (50 mg, 0.20 mmol) and thiazole-2-carbaldehyde (22 mg, 0.20 mmol). The target compound **17c** was obtained as yellow solid (33 mg). Yield: 47.4%, mp 273–275 °C. ^1^H NMR (600 MHz, DMSO-*d*_6_) δ 8.22 (d, *J* = 15.9 Hz, 1H, thiazole-4-*H*), 8.05 (s, 1H, quinazolinone-7-*H*), 7.96 (s, 1H, quinazolinone-5-*H*), 7.92 (s, 1H, CH=C*H*- thiazole), 7.81 (s, 1H, C*H*=CH-thiazole), 7.76 (d, *J* = 16.1 Hz, 1H, thiazole-5-*H*). ^13^C NMR (151 MHz, DMSO-*d*_6_) δ 183.41, 161.59, 158.39, 150.19, 137.40, 134.66, 132.98, 126.72, 125.76, 124.63, 122.81, 120.69, 117.85. HRMS (ESI) calcd. for C_14_H_7_Cl_2_N_3_O_2_S [M + H]^+^, 351.9709; found, 351.9692.

#### 3.2.17. Synthesis of (E)-6-methyl-2-(3-(thiazol-2-yl)acryloyl)quinazolin-4(3H)-one (**17d**)

Compound **17d** was prepared according to the procedure described for compound **4**, starting from compound **16d** (50 mg, 0.25 mmol) and thiazole-2-carbaldehyde (28 mg, 0.25 mmol). The target compound **17d** was obtained as yellow solid (58 mg). Yield: 39.4%, mp 280–282 °C. ^1^H NMR (600 MHz, DMSO-*d*_6_) δ 12.12 (s, 1H, quinazolinone-N*H*), 8.35 (d, *J* = 15.9 Hz, 1H, CH=C*H*-thiazole), 8.21 (s, 1H, quinazolinone-5-*H*), 8.13 (d, *J* = 3.1 Hz, 1H, thiazole-4-*H*), 8.06 (d, *J* = 3.0 Hz, 1H, thiazole-5-*H*), 8.01 (d, *J* = 15.9 Hz, 1H, C*H*=CH-thiazole), 7.88 (d, *J* = 7.8 Hz, 1H, quinazolinone-7-*H*), 7.80 (d, *J* = 7.3 Hz, 1H, quinazolinone-8-*H*), 2.44 (s, 3H, quinazolinone-C*H*_3_). ^13^C NMR (151 MHz, DMSO-*d*_6_) δ 182.26, 163.45, 161.65, 145.15, 144.90, 144.33, 135.05, 133.54, 125.90, 124.42, 122.72, 120.13, 117.10, 114.37, 25.46. HRMS (ESI) calcd. for C_15_H_11_N_3_O_2_S [M + H]^+^, 298.0645; found, 298.0644.

#### 3.2.18. Synthesis of (E)-8-methyl-2-(3-(thiazol-2-yl)acryloyl)quinazolin-4(3H)-one (**17e**)

Compound **17e** was prepared according to the procedure described for compound **4**, starting from compound **16e** (50 mg, 0.25 mmol) and thiazole-2-carbaldehyde (28 mg, 0.25 mmol). The target compound **17e** was obtained as yellow solid (50 mg). Yield: 34.0%, mp 207–209 °C. ^1^H NMR (600 MHz, DMSO-*d*_6_) δ 12.36 (s, 1H, quinazolinone-N*H*), 8.29 (d, *J* = 15.9 Hz, 1H, CH=C*H*-thiazole), 8.12 (d, *J* = 2.9 Hz, 1H, thiazole-4-*H*), 8.06 (d, *J* = 2.8 Hz, 1H, thiazole-5-*H*), 7.98 (d, *J* = 15.9 Hz, 1H, C*H*=CH-thiazole), 7.85 (d, *J* = 8.2 Hz, 1H, quinazolinone-5-*H*), 7.75 (d, *J* = 8.3 Hz, 1H, quinazolinone-7-*H*), 7.73–7.67 (m, 1H, quinazolinone-6-*H*), 2.45 (s, 3H, quinazolinone-C*H*_3_). ^13^C NMR (151 MHz, DMSO-*d*_6_) δ 183.21, 163.17, 161.22, 148.34, 147.82, 145.87, 136.54, 135.82, 129.11, 127.64, 126.18, 125.30, 123.46, 121.79, 21.51. HRMS (ESI) calcd. for C_15_H_11_N_3_O_2_S [M + H]^+^, 298.0645; found, 298.0646.

### 3.3. Biological Assay

#### 3.3.1. Antibacterial Activity

The prepared thiazolylketenyl quinazolinones (TQs) **4**–**8** and **17a**–**e** were evaluated for their antibacterial activities against five Gram-positive bacteria and six Gram-negative bacteria. The bacterial suspension was adjusted to 1 × 10^8^ CFU/mL in saline solution and then diluted in wells to achieve the final concentration of about 1 × 10^5^ CFU/mL. These compounds and norfloxacin were separately dissolved in dimethyl sulfoxide to prepare the stock solutions. The stock solutions were serially diluted in Mueller–Hinton broth (Guangdong Huaikai Microbial Sci. and Tech Co., Ltd., Guangzhou, China) to the desired concentrations in 96-well plates. The plates were followed by the addition of ~10^5^ CFU/mL bacterial suspension (100 μL) in each well and incubated at 37 °C for 24 h to determine minimum inhibitory concentrations (MICs). The assay of minimum bactericidal concentration (MBC) was further conducted by broth microdilution and agar plate colony counting. The MBCs of compound **4** against MRSA were determined by plating 10 μL of culture volume from the MIC assay onto an agar plate, and colony formation was examined after 24 h at 37 °C.

#### 3.3.2. Hemolytic Assay

The human red blood cells (RBCs) were collected and washed three times with saline. Subsequently, the cells were reallocated in saline to provide 5% *v*/*v* red blood cell suspension. Each well of 96-well plates was injected with 100 μL red cell suspension. TQ **4** (256 μg/mL, 100 μL) was added to the first column of plates and was double-serially diluted. The plates were incubated for different durations at 37 °C and then were centrifuged to extract the liquid supernatant of each hole. The absorbance of the supernatant was measured at 540 nm using a microplate reader. Distilled water and Triton X-100 were used as positive and negative controls, respectively. The hemolytic activity was calculated by the following equation: Hemolysis (%) = [(OD_sample_ − OD_saline_)/(OD _Triton X-100_ − OD_saline_)] × 100%.

#### 3.3.3. Resistance Study

The propensity of drug resistance induced by TQ **4** for MRSA was evaluated by sequential passaging method, and the MIC values were tested as described in biological assay procedures. After the MIC value was determined in the first generation, half-MIC suspension of resistant MRSA cells was taken for subculture, and every MIC value was obtained after 12 h incubation with TQ **4**. The process lasted for 16 days.

#### 3.3.4. ADMET Study

The related pharmacokinetic properties of TQ **4** were predicated using ADME (absorption, distribution, metabolism, and excretion) descriptors by a SwissADME online server (http://www.swissadme.ch/) (accessed on 2 April 2023). The bioavailability radar charts as well as pharmacokinetics and toxicity risk analysis are depicted in Figure S2, respectively.

#### 3.3.5. Inhibition of Biofilm

The bacterial cells of MRSA bacteria cultured in MHB medium overnight were used to prepare the bacterial suspension (approximately 1 × 10^6^ CFU/mL). Aliquots of 100 μL MRSA bacteria suspensions and TQ **4** at the different concentrations of 0, 0.5, 1. 2, 4, and 8 × MIC were incubated in a 96-well plate for 24 h at 37 °C. Next, methanol (150 μL) was added to fix the biofilm for 15 min. After removing methanol, the suspension was removed from the wells, and the biofilms were washed twice with phosphate buffer saline carefully to remove planktonic cells. The 0.1% crystal violet was added to each well for 30 min to stain biofilm. After crystal violet was discarded, the biofilm samples were washed with phosphate buffer saline and dried. Ethanol (150 µL, 95%) was added into each well to dissolve the crystal violet dye solution. The absorbance at 600 nm was measured using a microplate reader (Tecan Infinite M200 Pro, Thermo Fisher Scientific, Waltham, MA, USA). The % viability of the treated biofilms concerning the control was calculated and plotted. Dimethyl sulfoxide was used as a negative control. All samples were run in triplicates.

#### 3.3.6. Metabolic Activity

The MRSA suspensions (~10^5^ CFU/mL) were treated with increasing concentrations of TQ **4** for 6 h at 37 °C. Both control and treated cells were incubated with Alamar blue (50 μg/mL, 25 μL) for 20 min at 37 °C, then the fluorescence intensities were measured at excitation wavelength of 560 nm and emission wavelength of 590 nm. Dimethyl sulfoxide was used as a negative control. All samples were run in triplicates.

#### 3.3.7. Membrane Depolarization Assay

The MRSA strain was harvested (6000 rpm, 5 min), washed, and resuspended with 5 mM HEPES buffer, 5 mM glucose and 100 mM KCl solution at 1:1:1 ratio. The diSC35 dye (10 μM, 50 μL) and EDTA (200 μM, 50 μL) were added to the wells containing bacterial suspensions (~10^5^ CFU/mL, 100 μL) and preincubated for about 30 min. After cultivation, bacterial suspensions were removed, and wells were washed with phosphate-buffered saline. Fluorescence was measured at an excitation wavelength of 622 nm and an emission wavelength of 670 nm (negative control), then TQ **4** (1× MIC) was added, and the fluorescence intensity at different time intervals of TQ **4** was measured under the same condition. Dimethyl sulfoxide was used as a negative control. All samples were run in triplicates.

#### 3.3.8. Inner Membrane Permeability

The MRSA cells were cultured, washed, and resuspended in 5 mM glucose and 5 mM HEPES buffer (pH = 7.2) at a 1:1 ratio. The bacterial suspension (1 mL) was added with propidium iodide (PI) dye (10 μM, 50 μL) and various concentrations of TQ **4**, rendering that the final concentrations of TQ **4** in suspension should be 0, 0.5, 1, 2, 4, and 8 μg/mL. After coincubation for about 30 min, the fluorescence intensities were measured at excitation wavelength of 535 nm and emission wavelength of 617 nm. Dimethyl sulfoxide was used as a negative control. All samples were run in triplicates.

#### 3.3.9. Leakage of Cellular Protein

A range of concentrations of TQ **4** (0.5, 1, 2, 4, and 8 × MIC) were incubated with MRSA (~10^5^ CFU/mL). The bacterial suspension was added to an equal volume of 0.01 M phosphate buffer saline as a control. After the cultivation at 37 °C for 18 h, the mixtures were centrifuged (6000 rpm, 5 min), then the supernatant was collected. The concentration of leaked proteins in the supernatant was measured using a standard Bradford assay. Dimethyl sulfoxide was used as a negative control. All samples were conducted in triplicate.

#### 3.3.10. Reactive Oxygen Species (ROS) Production

The intracellular ROS levels were quantified with oxidation-sensitive DCFH-DA dye. The MRSA cells (~10^5^ CFU/mL) were treated with TQ **4** (0.5, 1, 2, 4, and 8 × MIC) at 37 °C for 18 h, then DCFH-DA dye (10 μM, 30 μL) was added into the bacterial suspension and incubated at 37 °C for 30 min under dark condition. After cultivation, the bacterial suspensions were removed, and wells were washed with phosphate-buffered saline. Fluorescence was measured at an excitation wavelength of 488 nm and an emission wavelength of 522 nm. Dimethyl sulfoxide was used as a negative control, and all samples were conducted in triplicate.

#### 3.3.11. Interactions of Compound **4** with Calf Thymus DNA

UV spectra were recorded at room temperature on a TU-2450 spectrophotometer (Puxi Analytic Instrument Ltd. of Beijing, China) equipped with 1.0 cm quartz cells. The stock solution of TQ **4** was prepared in DMF. Tris-HCl buffer solution (pH = 7.4) was prepared by mixing and diluting Tris (tris(hydroxymethyl)aminomethane) solution with HCl solution. Tris and HCl were analytical purity. DNA intercalation assay was carried out using calf thymus DNA as a substrate. TQ **4** was gradually added to the calf thymus DNA solution (c(DNA) = 1.56 × 10^−4^ mol/L) to make the final concentration of TQ **4** ranging from 0 to 2.67 × 10^−5^ mol/L.

Acridine Orange (AO) was added to the calf thymus DNA solution (c(DNA) = 1.56 × 10^−4^ mol/L). Then TQ 4 kept adding to the above solution to make the final concentration of TQ 4 ranging from 0 to 2.67 × 10^−5^ mol/L. The fluorescence spectral was recorded under each gradient.

#### 3.3.12. Measurement of PBP2a Contents

The 10^6^ CFU/mL of MRSA were treated with corresponding compounds for 6 h at 37 °C and 200 rpm. Following treatment, the cells were centrifuged at 5000 rpm for 5 min, and the supernatant was discarded. The PBP2a contents in control and treated cells were measured using PBP2a Elisa Assay Kit (Nanjing Herb Source Bio-Technology Co., Ltd, Nanjing, China).

#### 3.3.13. Drug Combination

The FIC index was determined by setting up the standard checkerboard assay in a 96-well plate as previously described for the evaluation of antimicrobial activities. Cefdinir was serially diluted along the abscissa in MHB, while TQ **4** was diluted along the ordinate to create a 7 × 7 matrix. FIC_a_ = MIC**_4_**_(combination)_/MIC**_4_**_(alone)_; FIC_b_ = MIC_cefdinir (combination)_/MIC_cefdinir (alone)_. FICI = FIC_a_ + FIC_b_. The FICI was interpreted as follows: A synergistic effect when FICI ≤ 0.5, an additive or indifferent effect when FICI > 0.5–2, and an antagonistic effect when FICI > 2–4.

#### 3.3.14. Determination of Allosteric Site Binding Affinity by Fluorescent Quenching

To investigate the binding of TQ **4** to the allosteric site of PBP2a, the transpeptidase active site of purified PBP2a was irreversibly acylated by incubation with an excess amount of oxacillin for 45 min at room temperature. Unbound oxacillin was removed with a protein desalting column (Pierce), and the acylated protein was used in subsequent experiments. Binding of TQ **4** to the allosteric site was determined by monitoring the intrinsic fluorescence of tryptophan and tyrosine residues of PBP2a. Using the fluorescence spectrofluorometer, PBP2a (500 nM) was incubated at room temperature in buffer containing HEPES (pH 7, 25 mM), NaCl (1 M) for 10 min. Upon excitation at 280 nm, the emission spectra of protein alone were taken. TQ **4** was titrated into the reaction, and the emission spectra were measured. The decrease in fluorescent emission at 330 nm for each concentration of TQ **4** was quantified. The normalized difference in fluorescence was plotted versus concentration. All the experiments were done at least in triplicate.

#### 3.3.15. Molecular Docking

Crystal structure of (PDB code: 4CJN) in PBD format was downloaded from Protein Data Bank. Sybyl-X 2.1.1 were used to perform docking with the ligand molecule.

## 4. Conclusions

In summary, a series of thiazolylketenyl quinazolinones and analogs was designed and synthesized as novel antibacterial agents. Bioactivity evaluation manifested that some target compounds possessed good to outstanding antibacterial properties. Especially TQ **4** showed 8 times more anti-MRSA activity than norfloxacin, with a MIC value of 0.5 μg/mL. Importantly, TQ **4** showed low hemolytic toxicity and drug resistance, not only inhibited biofilm formation but also reduced MRSA metabolic activity and displayed favorable drug-likeness. Further exploration implied that TQ **4** could not only inhibit the proliferation of MRSA through multiple actions but also could be used together with *β*-lactam antibiotics to treat MRSA infection. These results suggested that TQ **4** should have promise as a highly potent anti-MRSA agent and imply larger potential in combination use with *β*-lactams, which might be further developed as an allosteric agent for PBP2a.

## Figures and Tables

**Table 1:** Exploration of reaction conditions for the quinazolinone intermediates **12**.

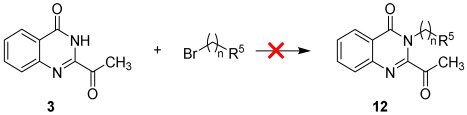					
Number	Solvent	Base	Temperature (°C)	Time (h)	Yields (%)
1	CH_3_CN	K_2_CO_3_	80	8	-
2	CH_3_CN	Cs_2_CO_3_	80	8	-
3	DMF	K_2_CO_3_	80	8	-
4	DMF	K_2_CO_3_	120	8	-
5	DMF	Cs_2_CO_3_	80	8	-
6	DMF	NaH	25	8	-
**7**	DMF	NaH	80	8	-

**Table 2:** Antibacterial data (MIC, μg/mL) for the target thiazolylketenyl quinazolinones and analogs **4**–**8** and **17a**–**e**.

Compds.	Gram-Positive Bacteria ^a^					Gram-Negative Bacteria ^b^					
	MRSA	*S. a.*	*S. a.* 25923	*S. a.* 29213	*E. f.*	*K. p.*	*E. c.*	*E. c.* 25922	*P. a.*	*P. a.* 27853	*A. b.*
**4**	0.5	4	8	1	2	2	2	0.5	4	2	64
**5a**	16	32	16	1	32	16	32	8	16	8	2
**5b**	64	64	128	32	128	256	128	32	128	128	256
**6a**	4	16	16	32	16	2	4	32	8	64	16
**6b**	8	8	8	16	16	16	8	64	16	128	16
**6c**	128	32	64	8	128	128	128	64	8	128	32
**7a**	8	32	64	128	256	64	4	32	16	16	2
**7b**	8	8	4	16	4	8	4	8	8	0.5	4
**8a**	16	32	8	8	32	2	32	16	4	64	4
**8b**	8	4	8	16	32	2	16	16	32	32	1
**8c**	64	8	4	8	16	128	32	32	64	16	32
**17a**	32	32	16	32	4	8	64	4	4	32	16
**17b**	128	8	16	8	8	4	32	4	4	2	8
**17c**	4	16	8	32	1	16	8	8	2	2	2
**17d**	8	16	32	64	2	4	16	64	2	4	4
**17e**	8	32	16	32	1	16	32	8	4	32	4
Norfloxacin	4	8	4	1	2	4	4	2	4	1	2

^a^ MRSA, Methicillin-Resistant *Staphylococcus aureus* (N315); *S. a.*, *Staphylococcus aureus*; *S. a.* 25923, *Staphylococcus aureus* ATCC 25923; *S. a.* 29213, *Staphylococcus aureus* ATCC 29213; *E. f.*, *Enterococcus faecalis*; ^b^ *K. p.*, *Klebsiella pneumonia*; *E. c.*, *Escherichia coli*; *E. c.* 25922, *Escherichia coli* ATCC 25922; *P. a.*, *Pseudomonas aeruginosa*; *P. a.* 27853, *Pseudomonas aeruginosa* ATCC 27853; *A. b.*, *Acinetobacter baumanii*.

**Table 3:** Physicochemical and bioavailability properties of compound **4** and norfloxacin.

Properties	4	Norfloxacin
Molecular weight (g/mol) (<500)	283.31	319.33
MLOGP (≤4.15)	0.84	1.04
H-bond acceptors (<10)	4	5
H-bond donors (<5)	1	2
rotatable bonds (<10)	3	3
Lipinski’s Rule	Yes	Yes
Bioavailability score	0.55	0.55
GI absorption	High	High
BBB permean	No	No

**Table 4:** Contents of PBP2a in MRSA and combination effect of compound **4** and cefdinir on MRSA (MIC, μg/mL).

Compounds	PBP2a Contents in Treated MRSA (pg/mL)	MIC (Alone or Combination)
Control	405.94 ± 21.19	–
Compound **4**	345.16 ± 2.80	0.5 (alone)
Cefdinir	189.00 ± 11.04	1 (alone)
Compound **4** + Cefdinir	127.47 ± 14.94	0.125/0.125 (combination)

FIC_a_ = MIC**_4_**_(combination)_/MIC**_4_**_(alone)_; FIC_b_ = MIC_cefdinir (combination)_/MIC_cefdinir (alone)_. FICI = FIC_a_ + FIC_b_. The FICI was interpreted as follows: a synergistic effect when FICI ≤ 0.5, an additive or indifferent effect when FICI > 0.5–2, and an antagonistic effect when FICI > 2–4.

## Data Availability

All data are available based on “MDPI Research Data Policies” at https://www.mdpi.com/ethics (accessed on 17 May 2023).

## Supplementary Materials

The following supporting information can be downloaded at: https://www.mdpi.com/article/10.3390/molecules28104240/s1, Table S1: Antibacterial data (MIC, μg/mL) for thiazolylketenyl quinazolinone 4 against MRSA and *S. aureus*; Table S2: Minimum bactericidal concentration (MBC, μg/mL) of active compound **4** against MRSA, Figure S1: Images of RBC processed with compound **4** at different times; Figure S2: Bioavailability radar charts from Swiss ADME web tool for compound **4** and norfloxacin; Figure S3: The plot of A0/(A−A0) versus 1/[compound **4**], yielding the binding constant, K = 1.01 × 105 L/mol, R = 0.996, SD = 0.039 (R is the correlation coefficient. SD is standard deviation); Spectra of compound.

## Sample Availability

Samples of the compounds are available from the authors.
