# Supplementary material for: Novel Thiazolylketenyl Quinazolinones as Potential Anti-MRSA Agents and Allosteric Modulator for PBP2a
Source: Molecules. 2023 May 22;28(10):4240. doi: 10.3390/molecules28104240 (PMC10221040; doi:10.3390/molecules28104240)
Supplement: Supplementary file 1 [file molecules-28-04240-s001.zip › molecules-2392517-supplementary.pdf]

Supplementary Information

Title:

Novel Thiazolylketenyl Quinazolinones and Analogues as Potential Anti-MRSA Agents and Allosteric Modulator for PBP2a

Author names and affiliations:

Jie Dai [1], Narsaiah Battini [1], Zhonglin Zang [1],*,Yan Luo [2],* and Chenghe Zhou [1],*

[1] *Institute of Bioorganic & Medicinal Chemistry, Key Laboratory of Applied Chemistry of Chongqing Municipality, School of Chemistry and Chemical Engineering, Southwest University, Chongqing 400715, China.*

[2] *College of Pharmacy, National & Local Joint Engineering Research Center of Targeted and Innovative Therapeutics, Chongqing Key Laboratory of Kinase Modulators as Innovative Medicine, Chongqing University of Arts and Sciences, Chongqing, 402160, China*

*Corresponding authors:

Tel.: +86-23-68254967; Fax: +86-23-68254967, E-mail: zhouch@swu.edu.cn (Cheng-He Zhou); zhonglinzang@swu.edu.cn (Zhong-Lin Zang); lygytha456@163.com (Yan Luo)

1. Antibacterial activity

Table S1 Antibacterial data (MIC, μg/mL) for thiazolylketenyl quinazolinone **4** against MRSA and *S. aureus*.

Compds	MRSA 43300	*S. a.* 26003	*S. a.* 26001	*S. a.* 12600	*S. a.* 6538
**4**	1	8	4	1	2
Norfloxacin	4	1	4	2	4

Table S2 Minimum bactericidal concentration (MBC, μg/mL) of active compound **4** against MRSA.

Bacteria	Compound	MIC	MBC	MBC/MIC	Effect
MRSA	**4**	0.5	8	16	Bacteriostatic

2. Hemolytic activity

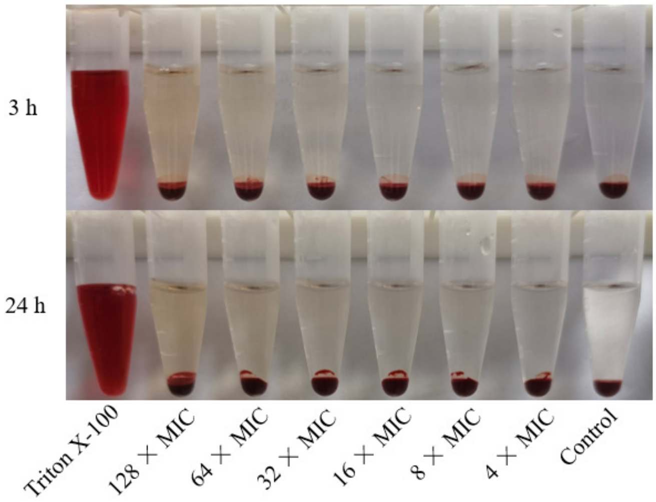

Figure S1 Images of RBC processed with compound **4** at different times.

3. ADMET study

The radar plot bears six axes for six important properties for oral bioavailability: saturation (INSATU), size (SIZE), polarity (POLAR), solubility (INSOLU), lipophilicity (LIPO) and flexibility (FLEX). **Figure S2** showed that compound **4** almost fall in the pink area and reference drug norfloxacin was completely in this area, which revealed that candidate compound **4** had good drug-likeness and bioavailability profile.

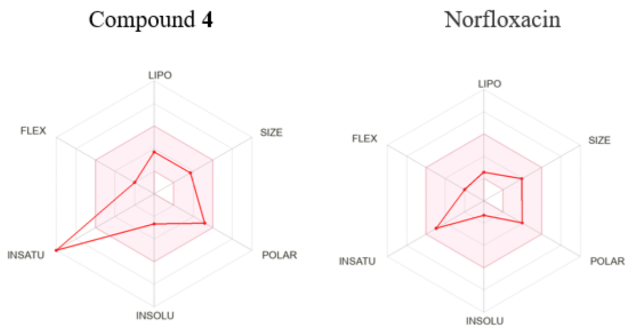

Figure S2 Bioavailability radar charts from swiss ADME web tool for compound **4** and norfloxacin.

4. Interactions of 7 with calf thymus DNA

Equation (1) is utilized to calculate the binding constant (K). Where A^0 and A are the absorbance of DNA in the absence and presence of compound **4** at 260 nm, ξ_C and ξ_{D-C} represent the absorption coefficients of compound **4** and compound **4**-DNA complex respectively (**Figure S3**).

$$\frac{A^0}{A-A^0}=\frac{\xi_C}{\xi_{D-C}-\xi_C}+\frac{\xi_C}{\xi_{D-C}-\xi_C}\times\frac{1}{K[\mathrm{Q}]} \quad (1)$$

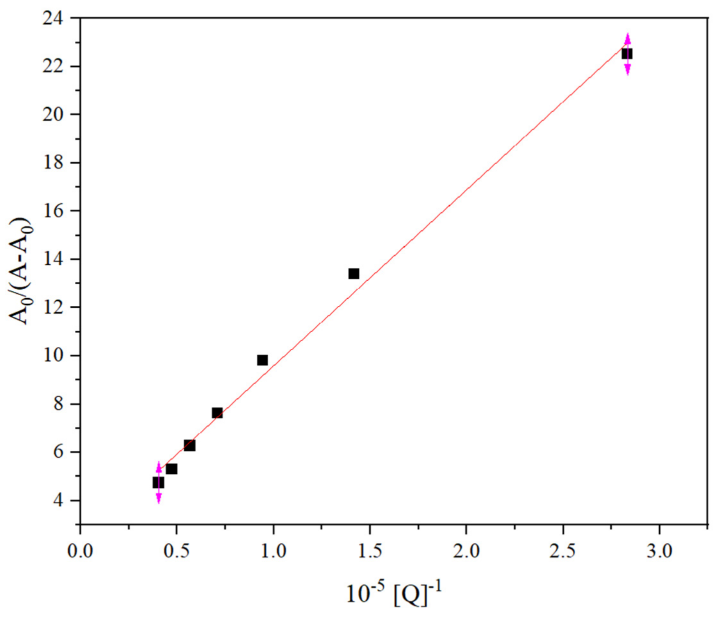

Figure S3 The plot of A^0 / $(A\text{-}A^0)$ versus 1/[compound **4**], yielding the binding constant, K = 1.01 × 10^5 L/mol, R = 0.996, SD = 0.039 (R is the correlation coefficient. SD is standard deviation).

5. Characterizations of some representative compounds

5.1 Spectra of compound **4**

^{1}H NMR spectrum

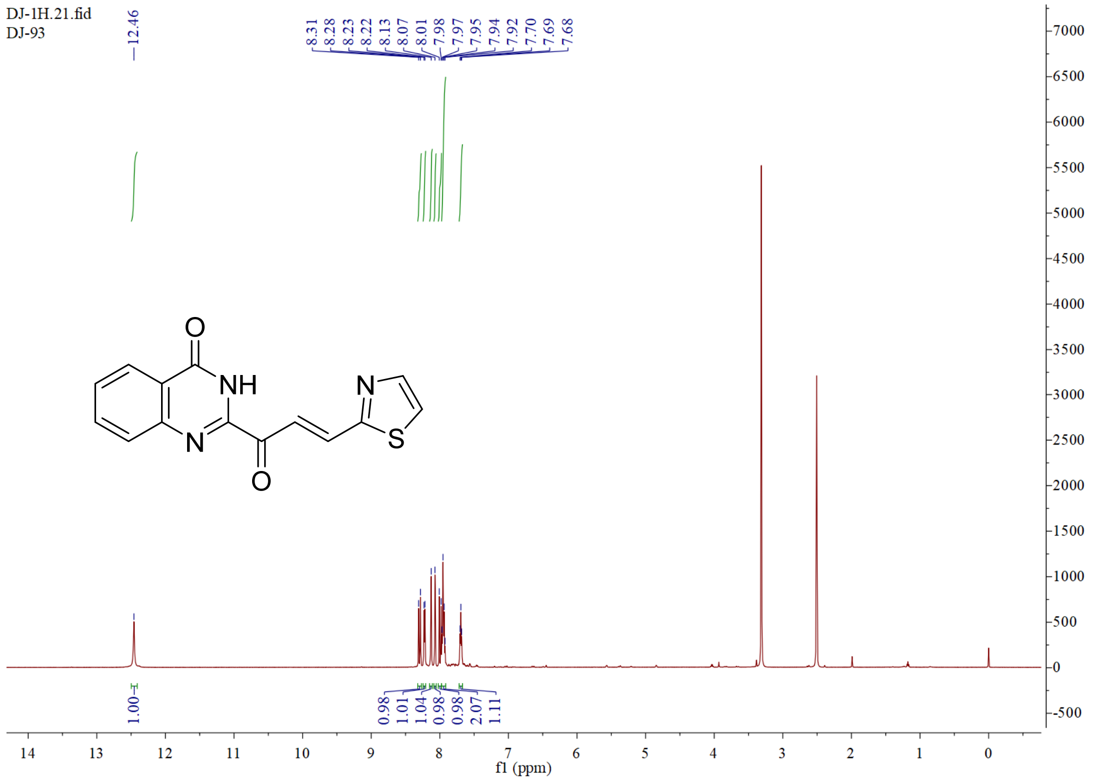

^{13}C NMR spectrum

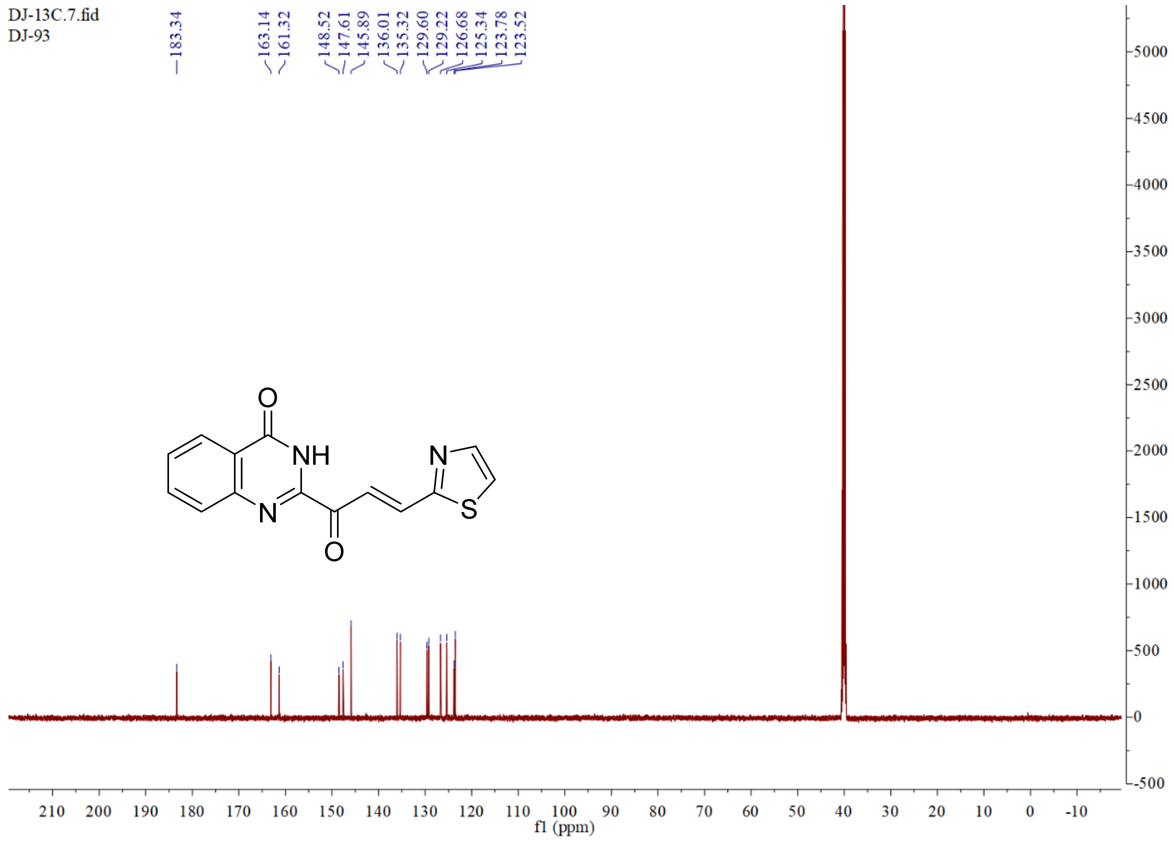

HRMS spectrum

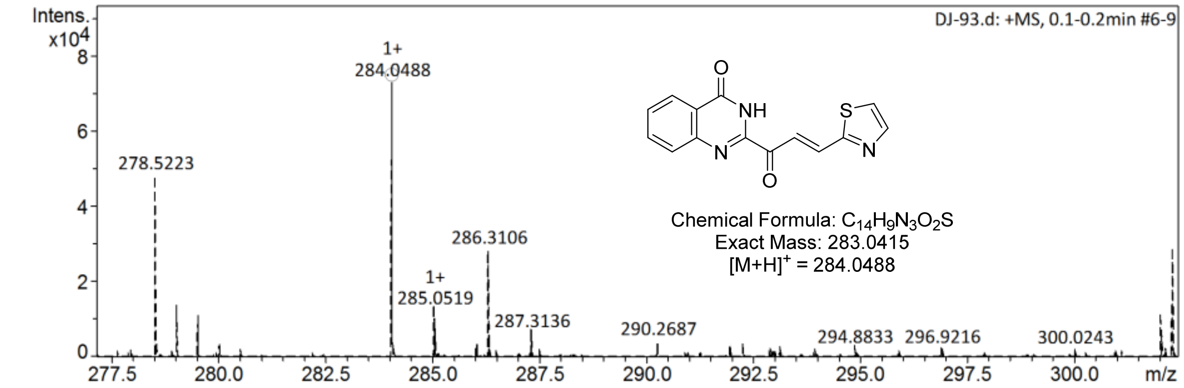

Meas. m/z	#	Ion Formula	Sum Formula	m/z	Adduct	err [ppm]	z	mSigma	Score	rdb	N-Rule	e^- Conf
284.0488	1	C14H10N3O2S	C14H9N3O2S	284.0488	M+H	0.1	1+	7.8	100.00	12.0	ok	even

HPLC spectrum

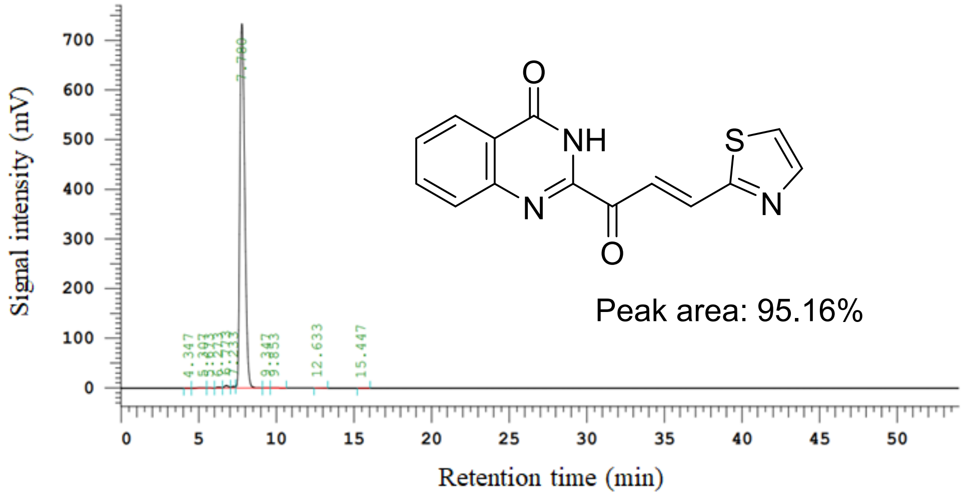

5.2 Spectra of compound **5a**

^{1}H NMR spectrum

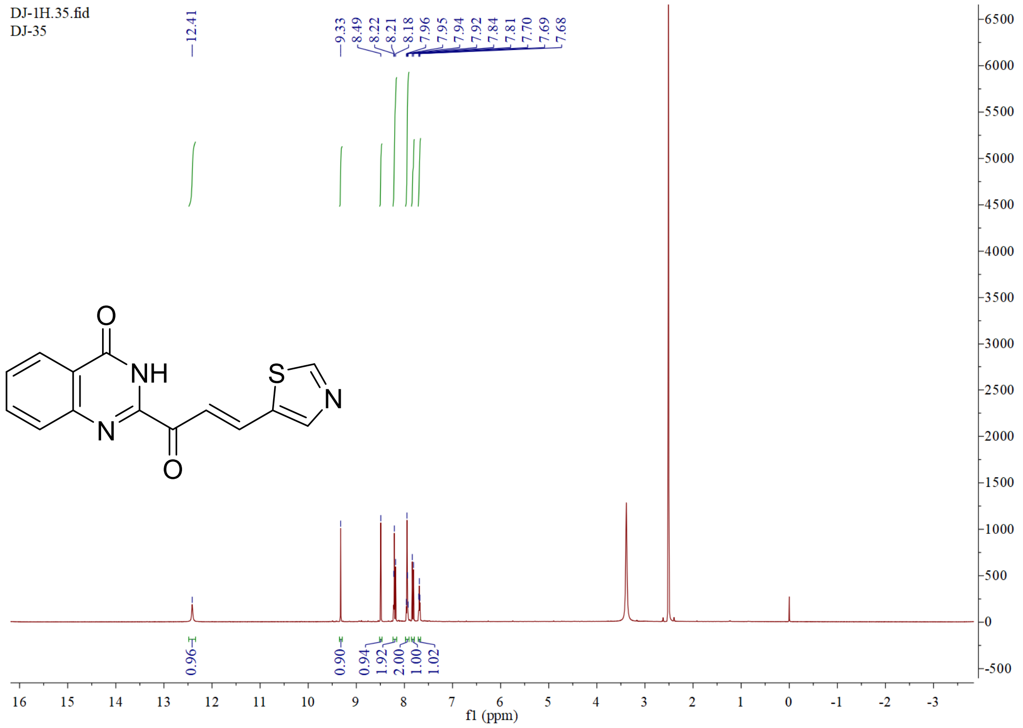

^{13}C NMR spectrum

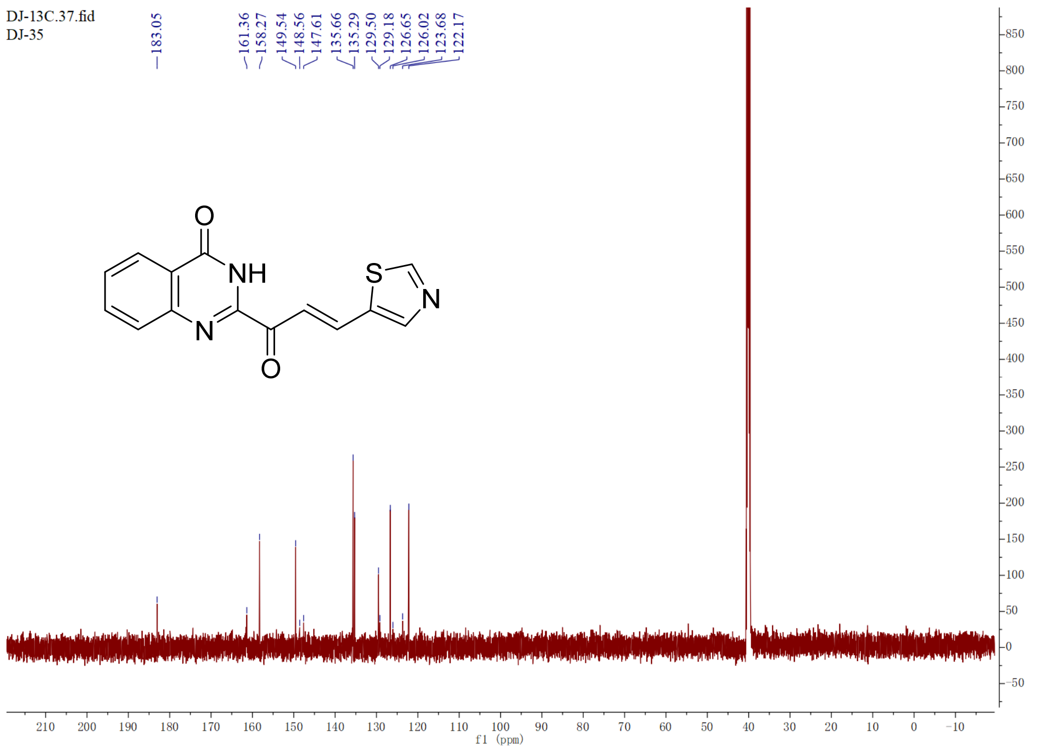

HRMS spectrum

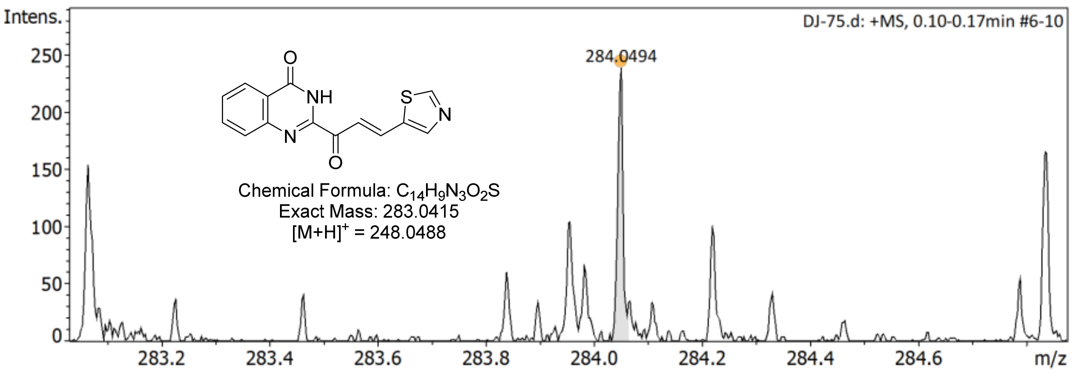

Meas. m/z	#	Ion Formula	m/z	err [ppm]	mSigma	# mSigma	Score	rdb	e¡¥ Conf	N-Rule
284.0494	1	C14H10N3O2S	284.0488	-1.9	n.a.	1	100.00	14.0	even	ok

HPLC spectrum

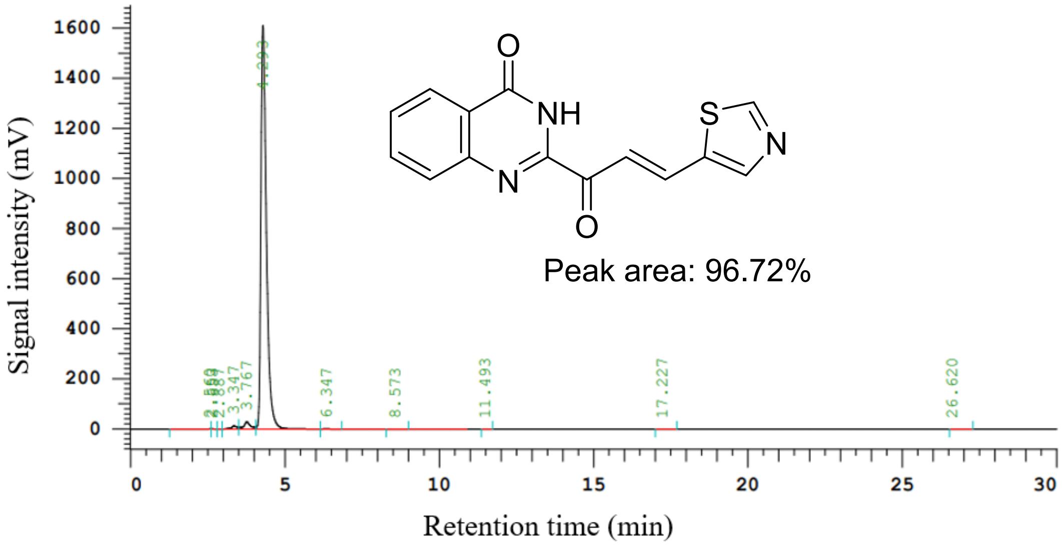

5.3 Spectra of compound **5b**

^{1}H NMR spectrum

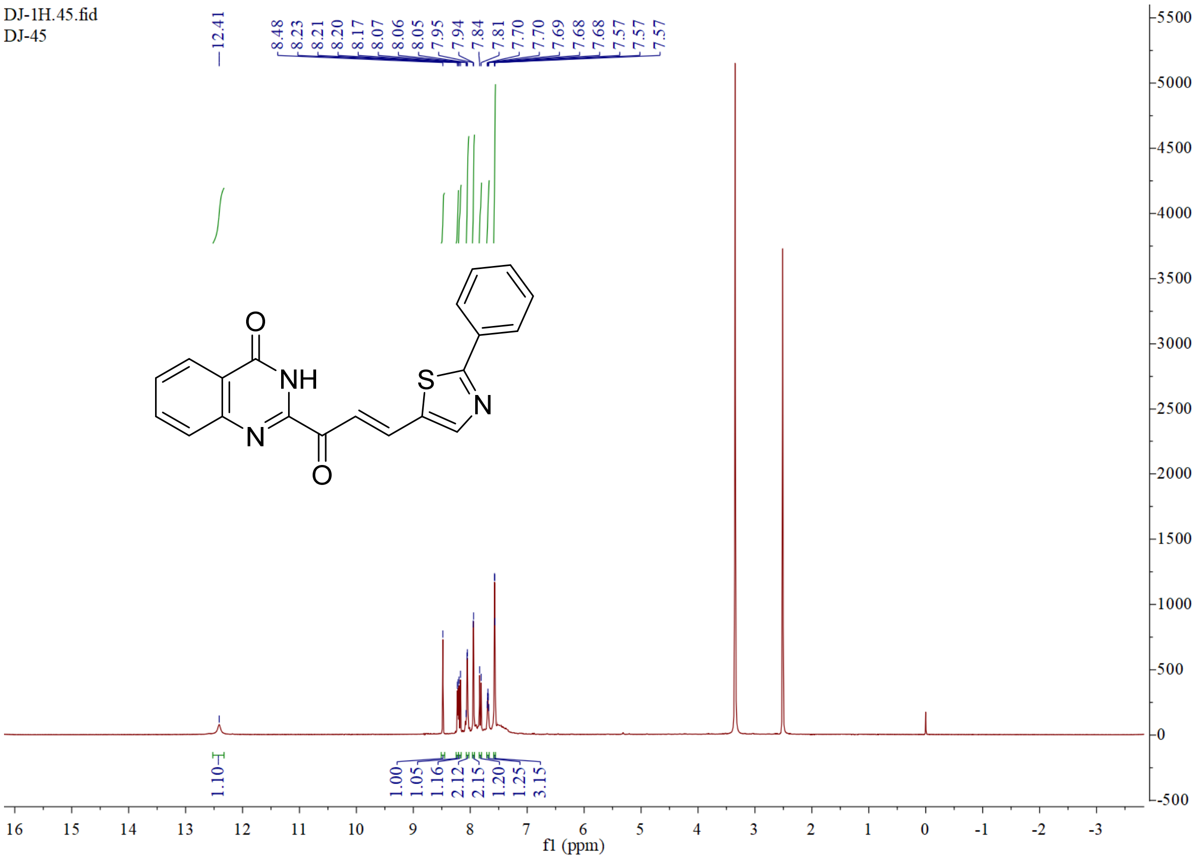

HRMS spectrum

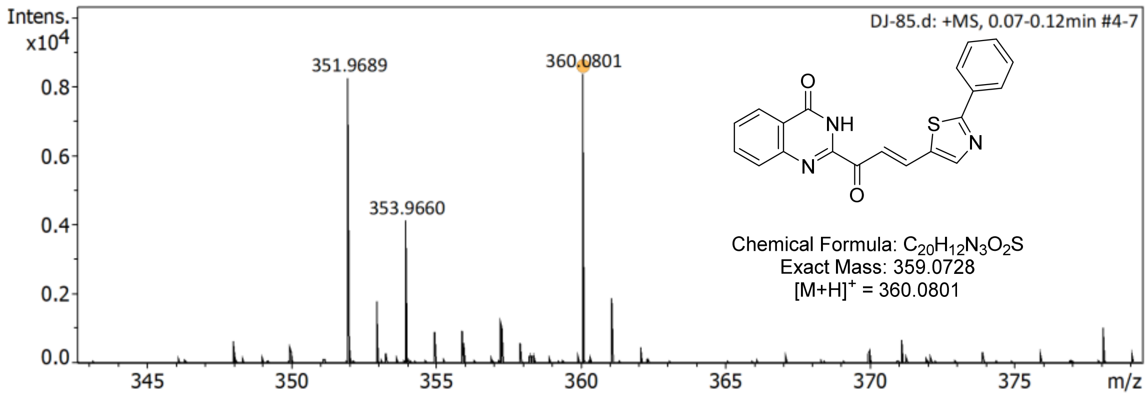

Meas. m/z	#	Ion Formula	m/z	err [ppm]	mSigma	# mSigma	Score	rdb	e¡¥ Conf	N-Rule
360.0801	1	C20H14N3O2S	360.0801	-0.1	39.2	1	100.00	18.0	even	ok

5.4 Spectra of compound **6a**

^{1}H NMR spectrum

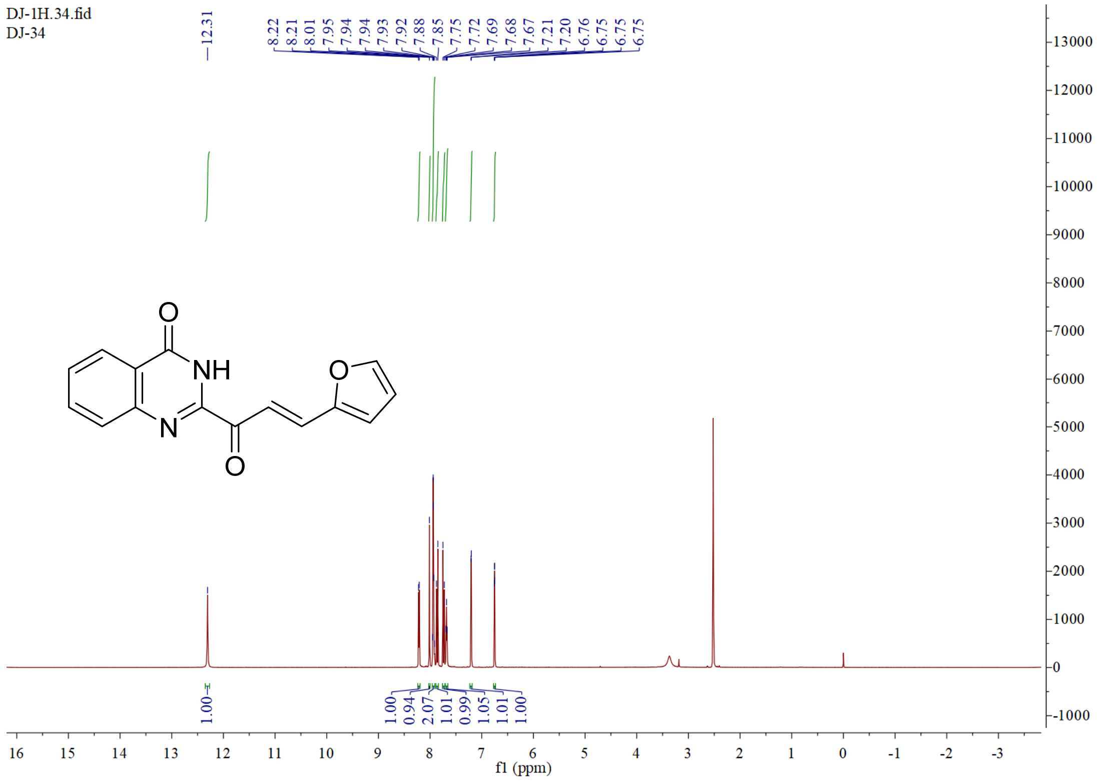

^{13}C NMR spectrum

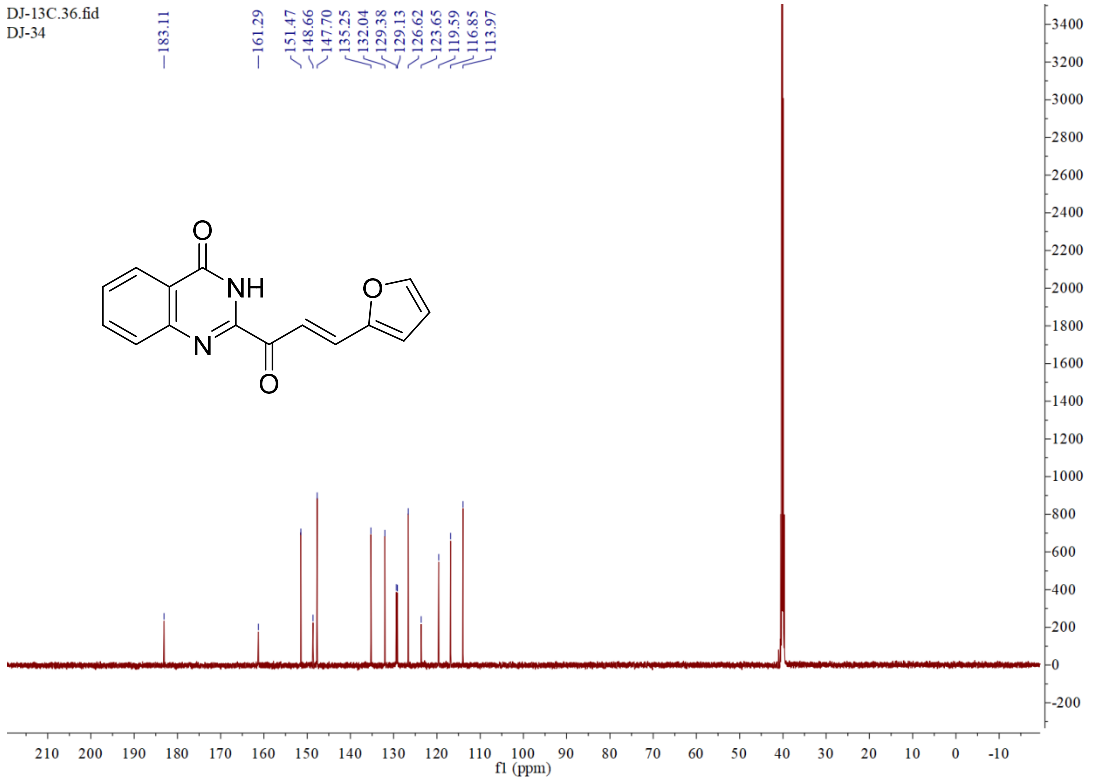

HRMS spectrum

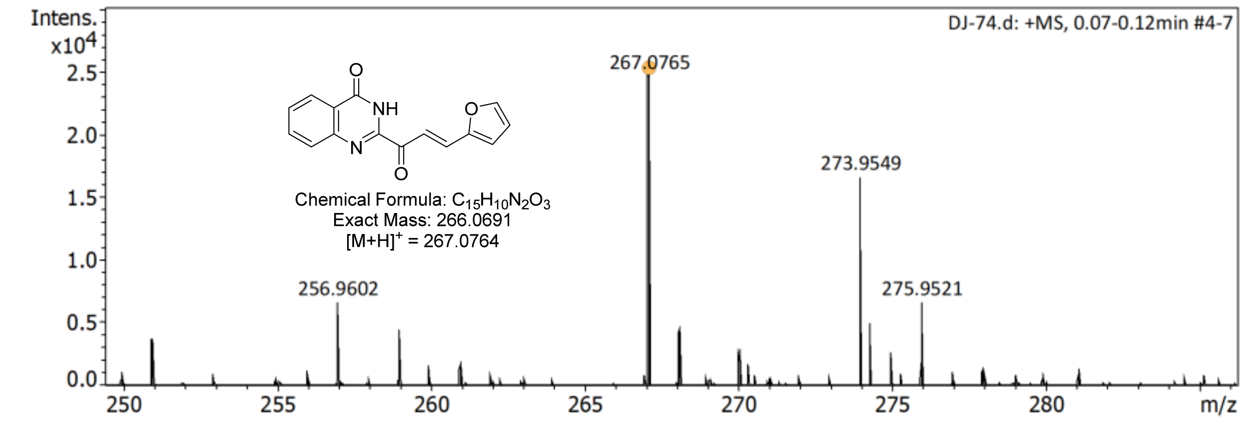

Meas. m/z	#	Ion Formula	m/z	err [ppm]	mSigma	# mSigma	Score	rdb	e¡¥ Conf	N-Rule
267.0765	1	C15H11N2O3	267.0764	-0.4	n.a.	1	100.00	12.0	even	ok

HPLC spectrum

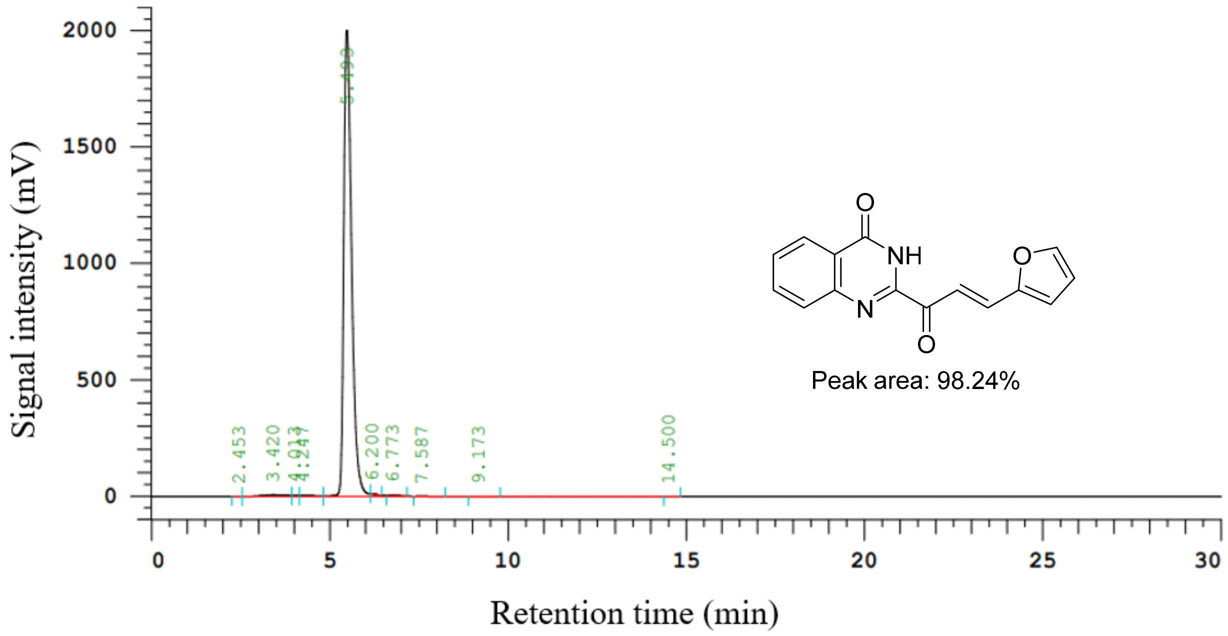

5.5 Spectra of compound **6b**

^{1}H NMR spectrum

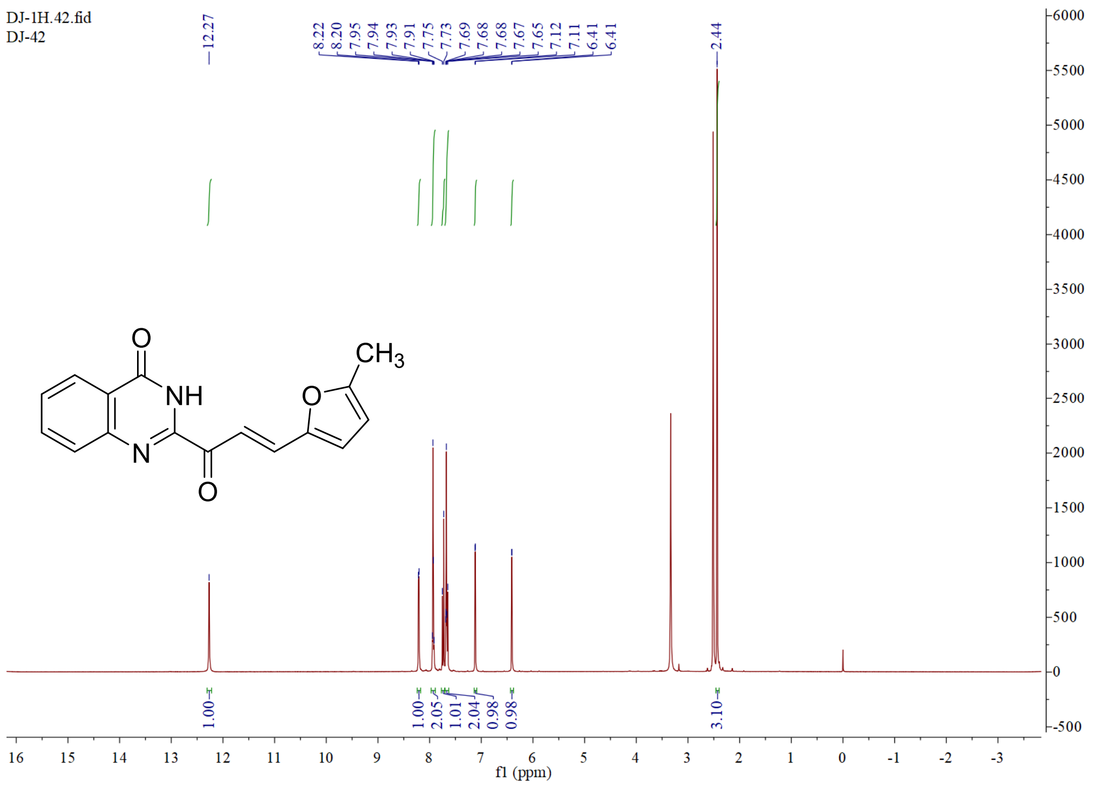

^{13}C NMR spectrum

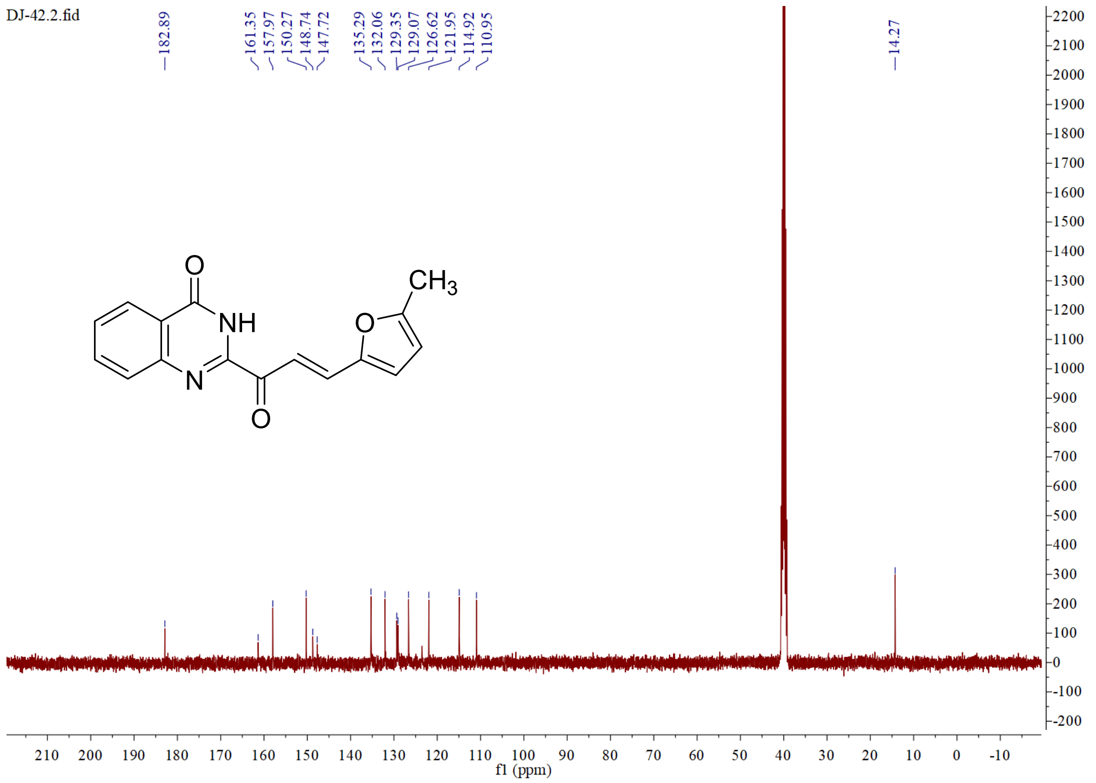

HRMS spectrum

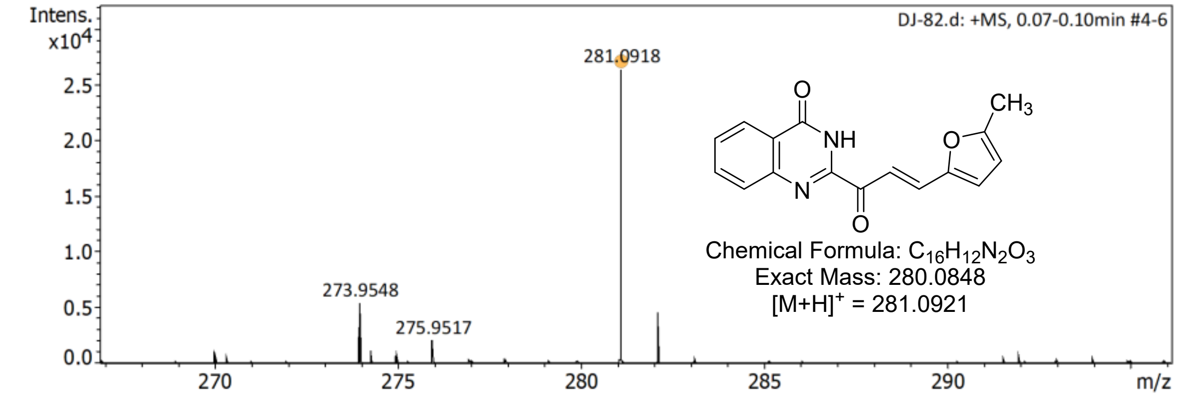

Meas. m/z	#	Ion Formula	m/z	err [ppm]	mSigma	# mSigma	Score	rdb	e¡¥ Conf	N-Rule
281.0918	1	C16H13N2O3	281.0921	1.1	13.5	1	100.00	12.0	even	ok

HPLC spectrum

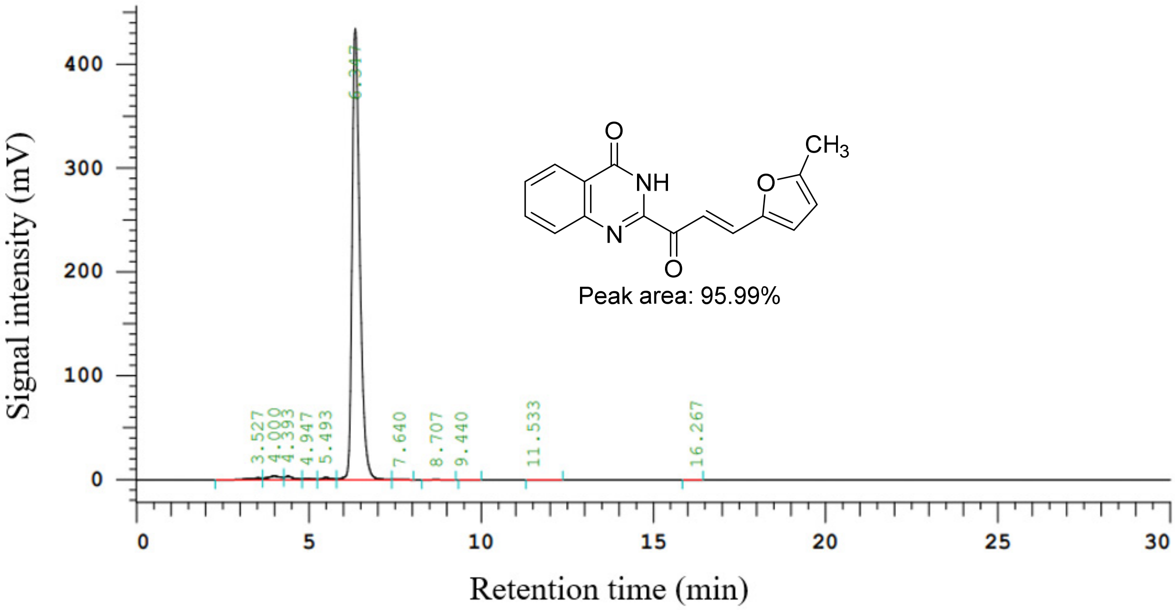

5.6 Spectra of compound **6c**

^{1}H NMR spectrum

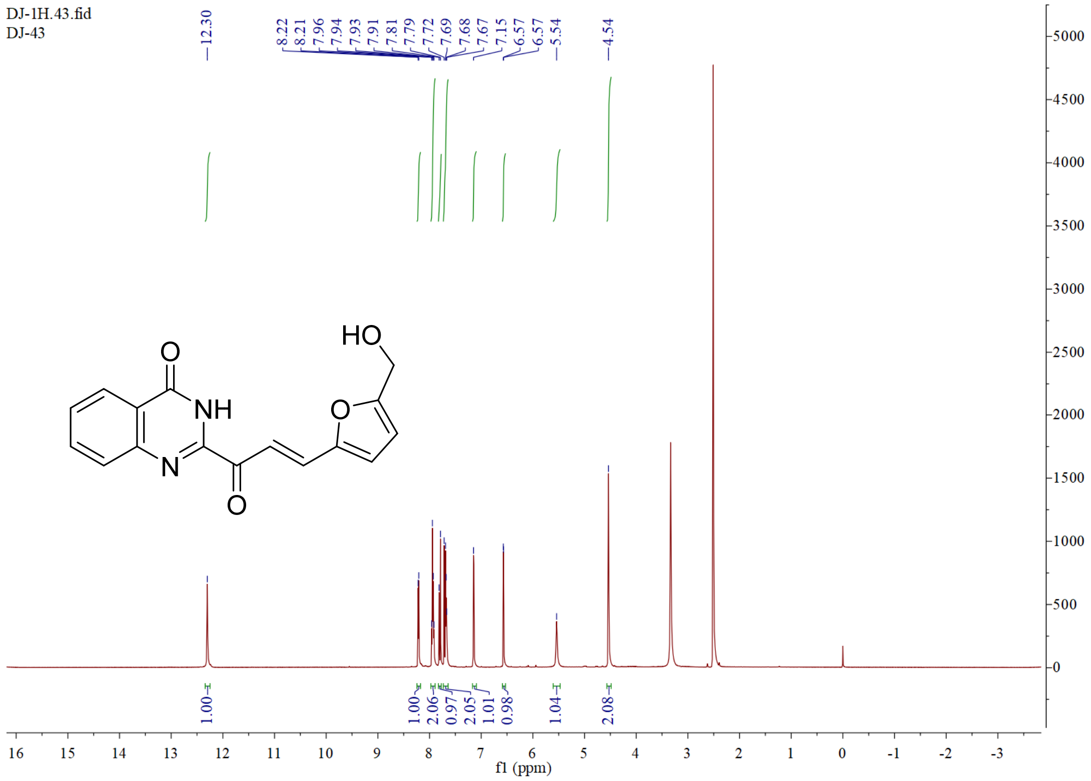

^{13}C NMR spectrum

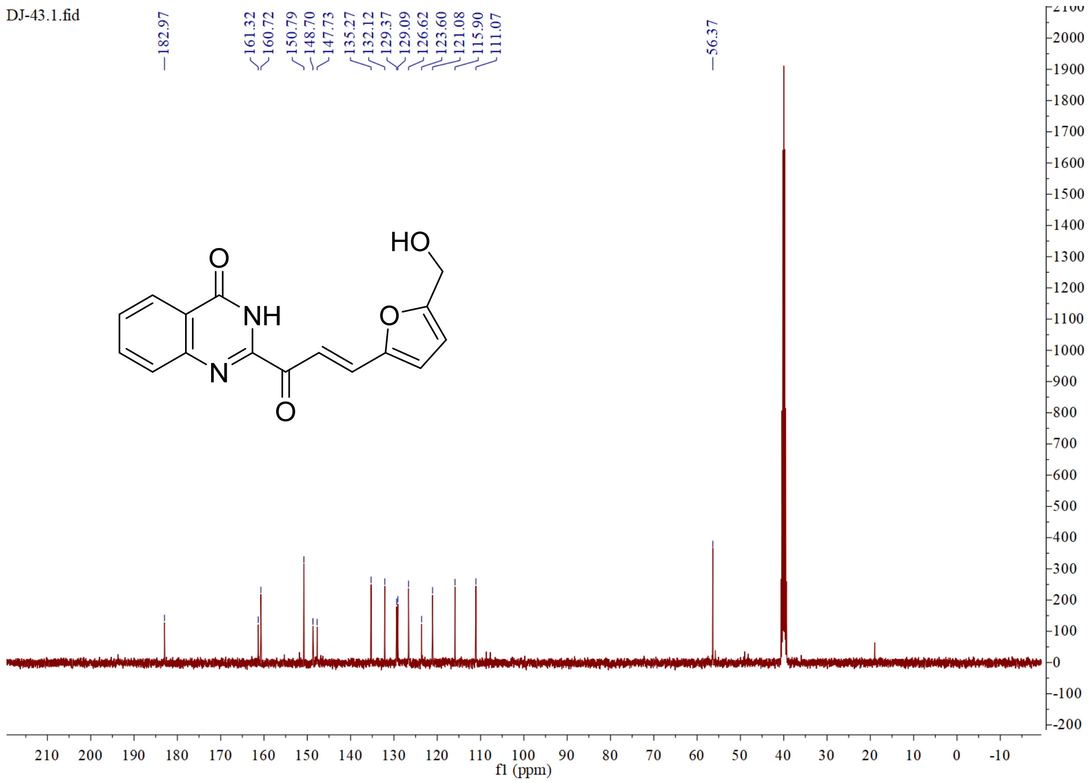

HRMS spectrum

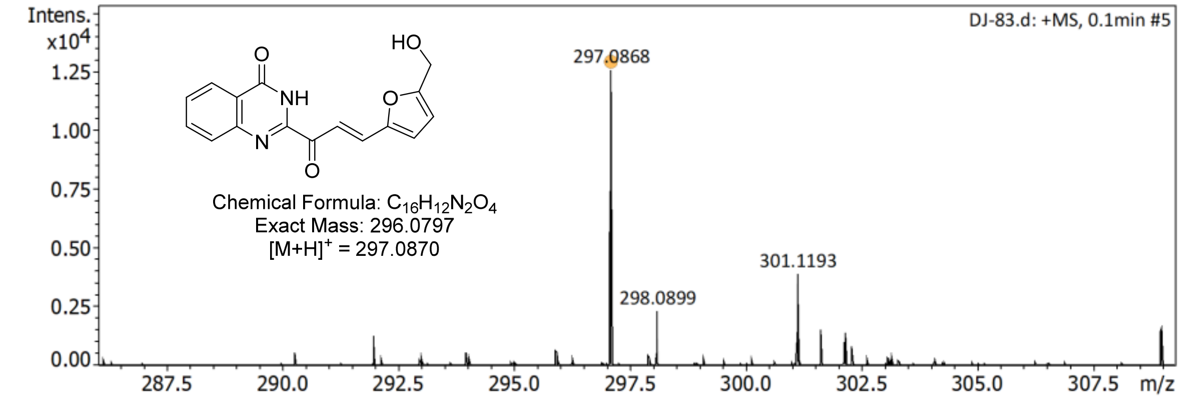

Meas. m/z	#	Ion Formula	m/z	err [ppm]	mSigma	# mSigma	Score	rdb	e¡¥ Conf	N-Rule
297.0868	1	C16H13N2O4	297.0870	0.5	13.9	1	100.00	12.0	even	ok

HPLC spectrum

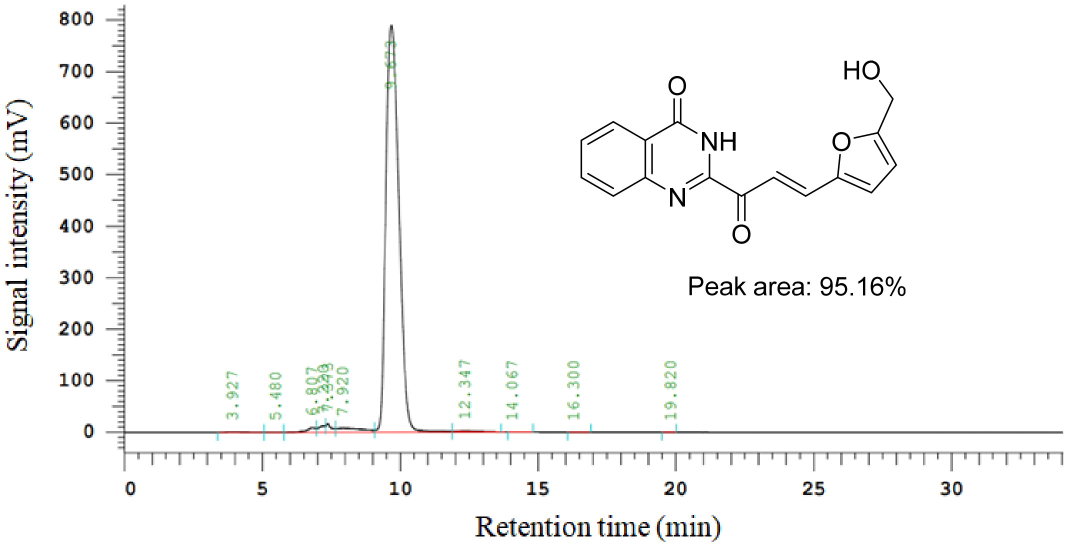

5.7 Spectra of compound **7a**

^{1}H NMR spectrum

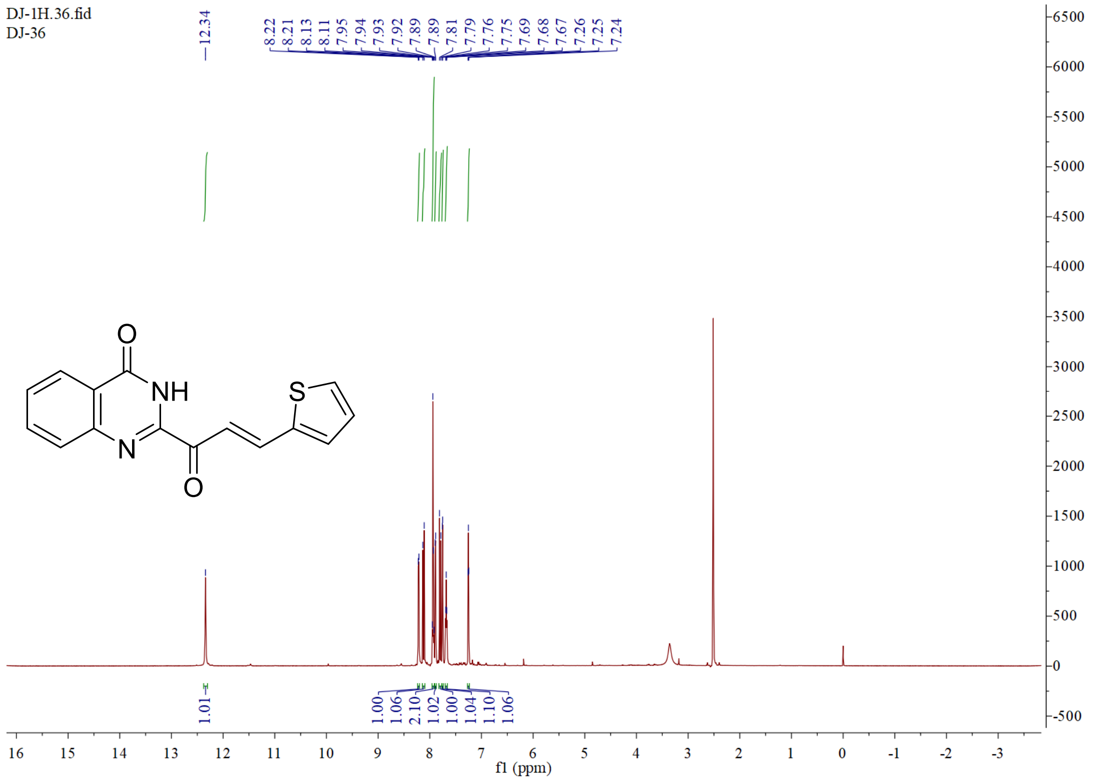

^{13}C NMR spectrum

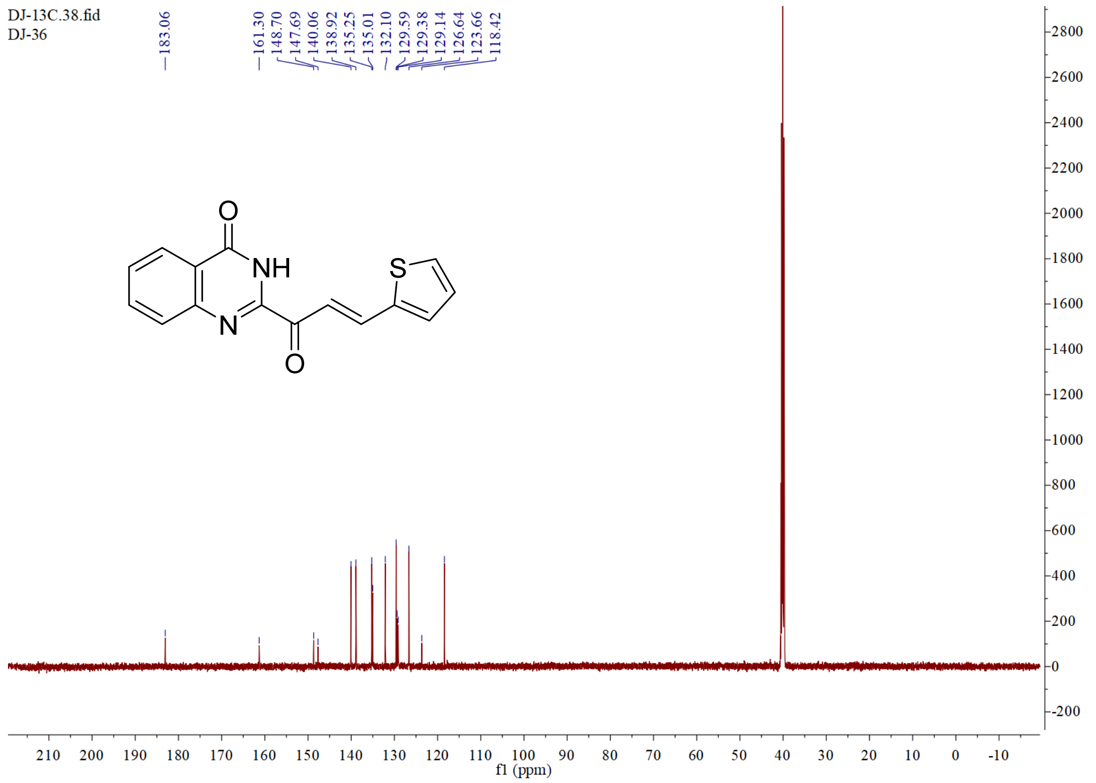

HRMS spectrum

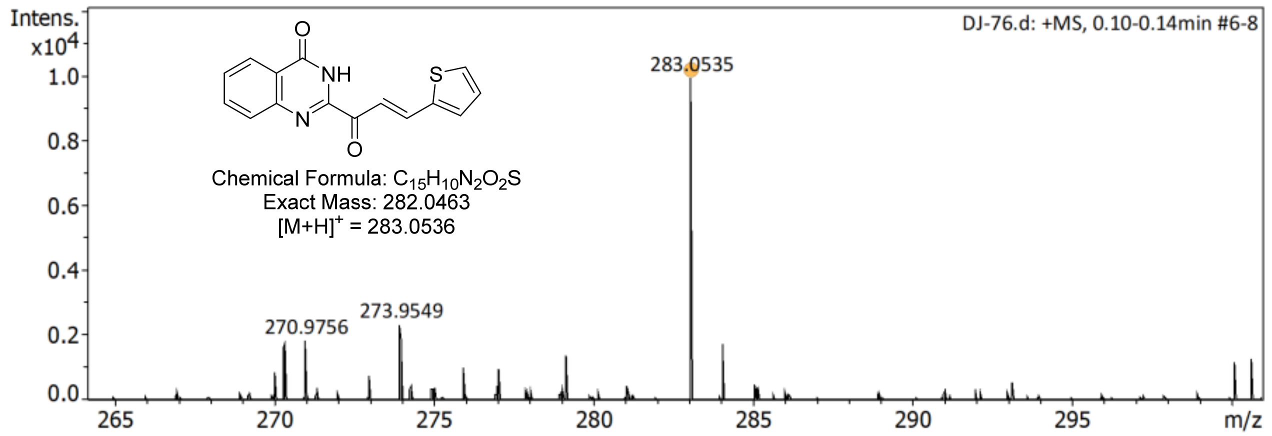

Meas. m/z	#	Ion Formula	m/z	err [ppm]	mSigma	# mSigma	Score	rdb	e¡¥ Conf	N-Rule
283.0535	1	C15H11N2O2S	283.0536	0.3	n.a.	1	100.00	14.0	even	ok

HPLC spectrum

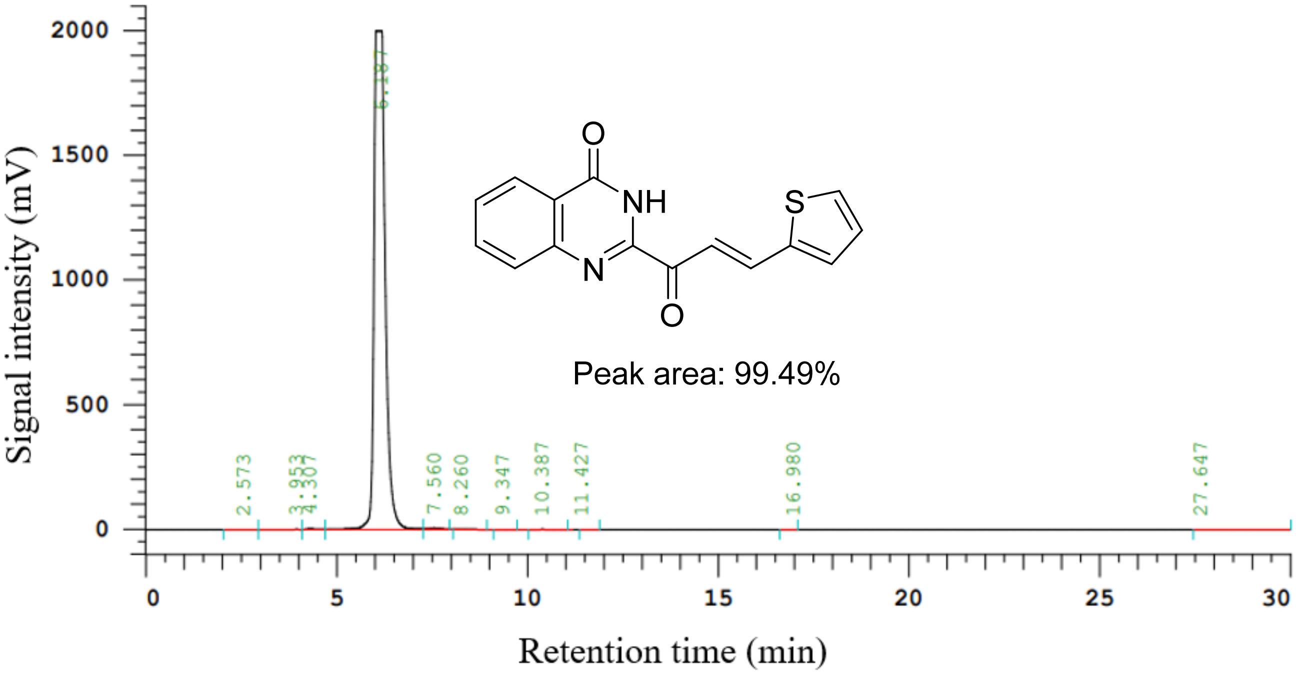

5.8 Spectra of compound **7b**

^{1}H NMR spectrum

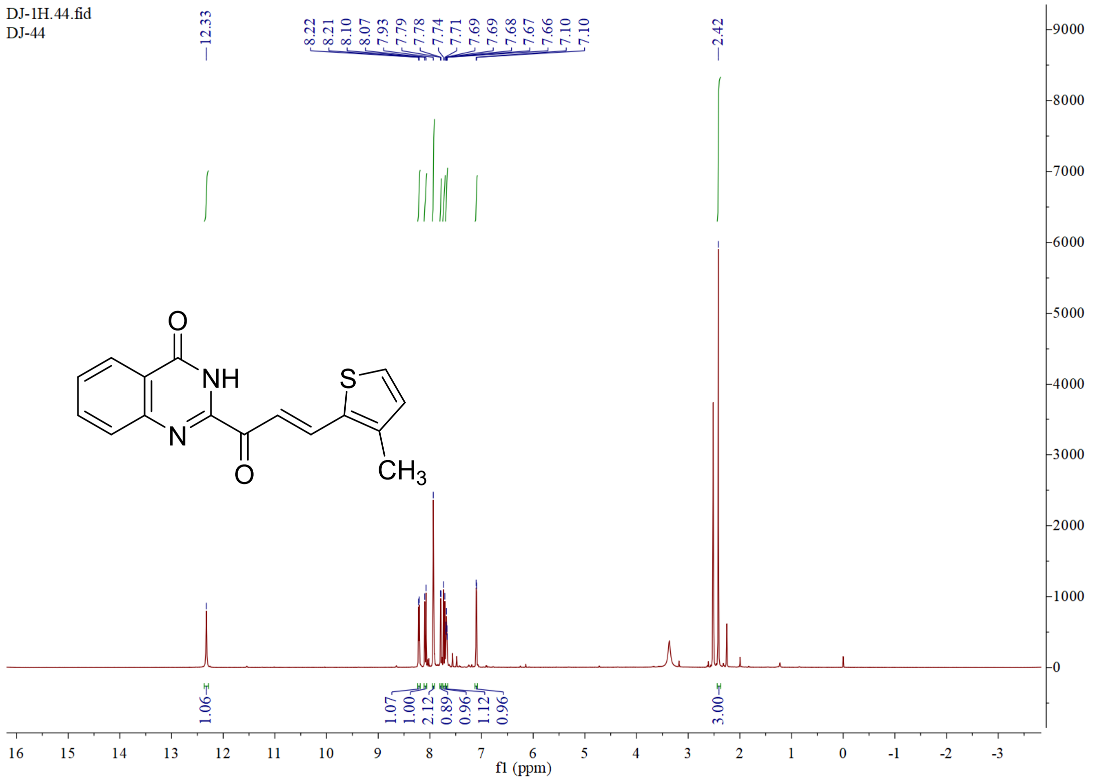

^{13}C NMR spectrum

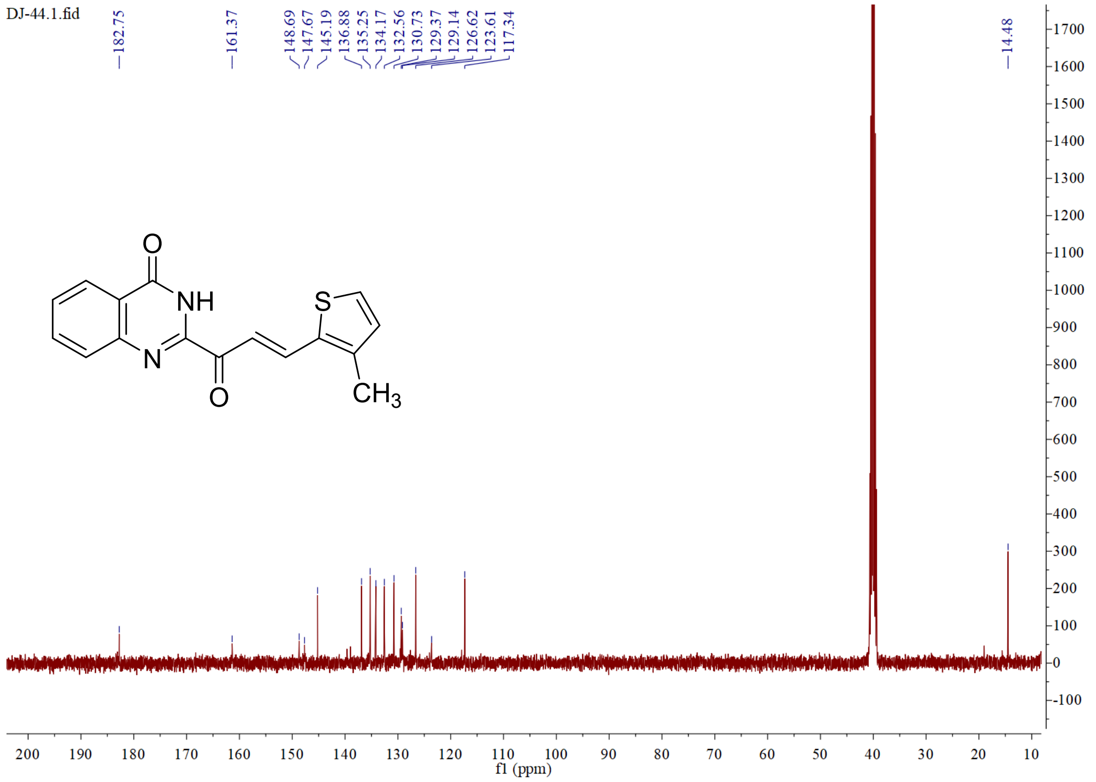

HRMS spectrum

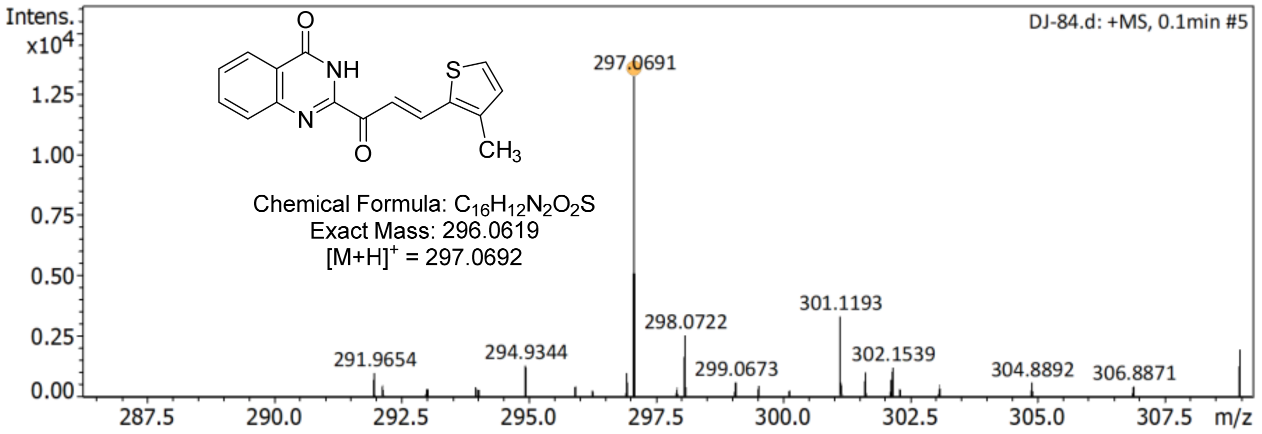

Meas. m/z	#	Ion Formula	m/z	err [ppm]	mSigma	# mSigma	Score	rdb	e¡¥ Conf	N-Rule
297.0691	1	C16H13N2O2S	297.0692	0.5	10.7	1	100.00	14.0	even	ok

HPLC spectrum

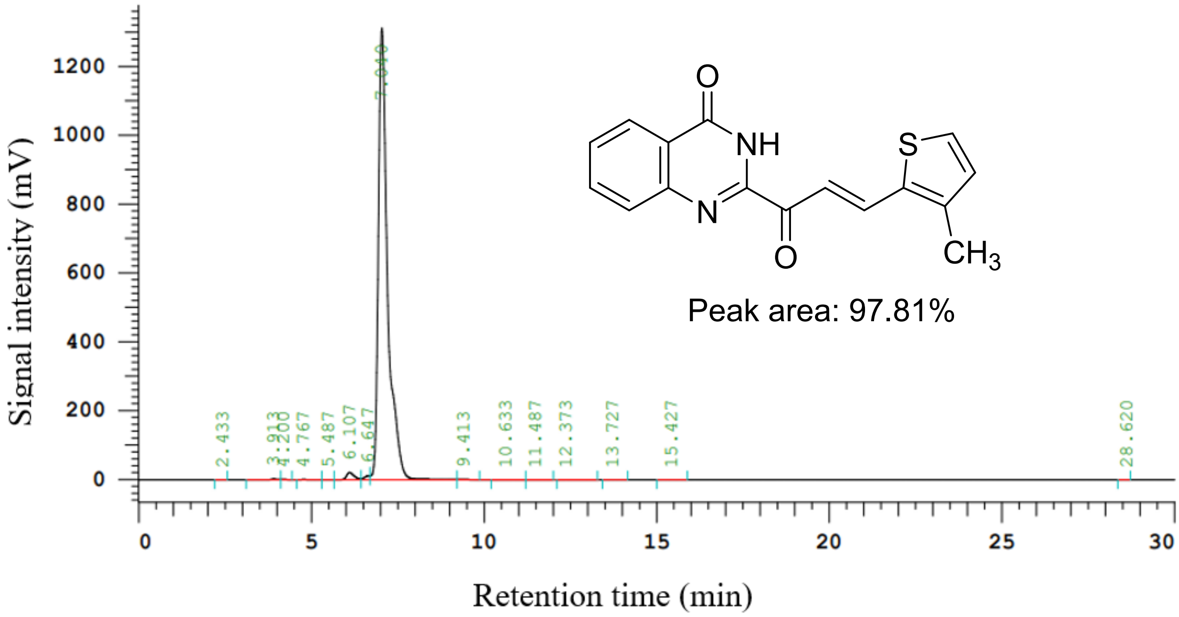

5.9 Spectra of compound **8a**

^{1}H NMR spectrum

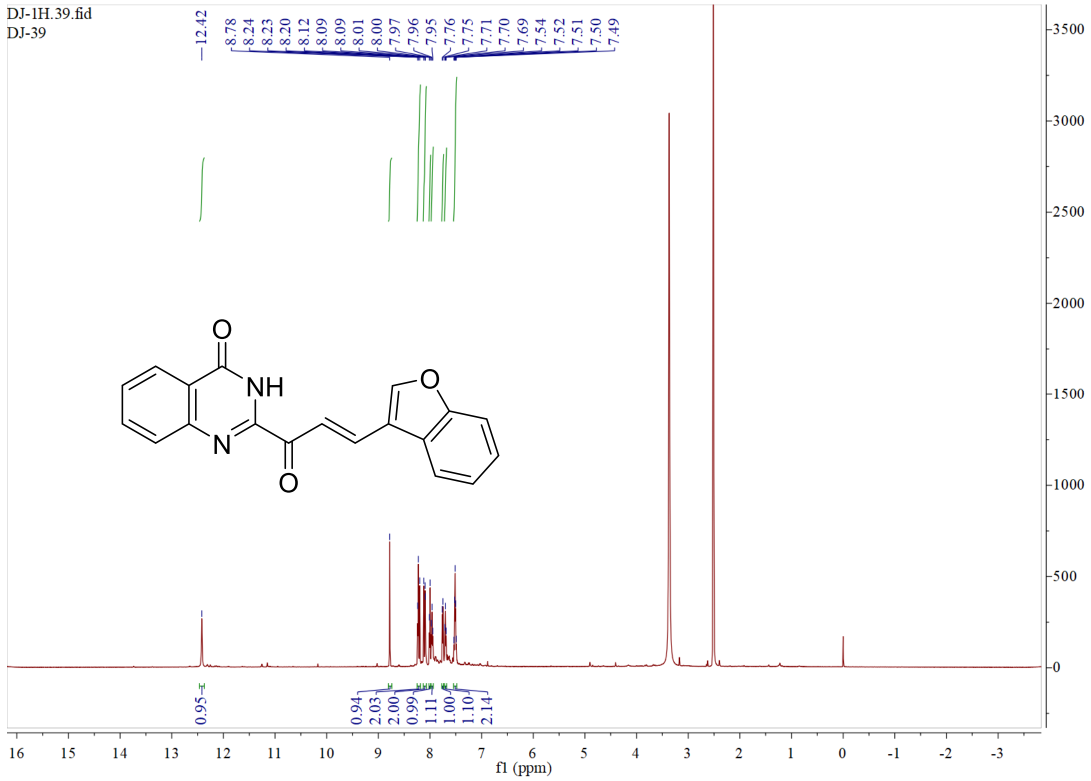

^{13}C NMR spectrum

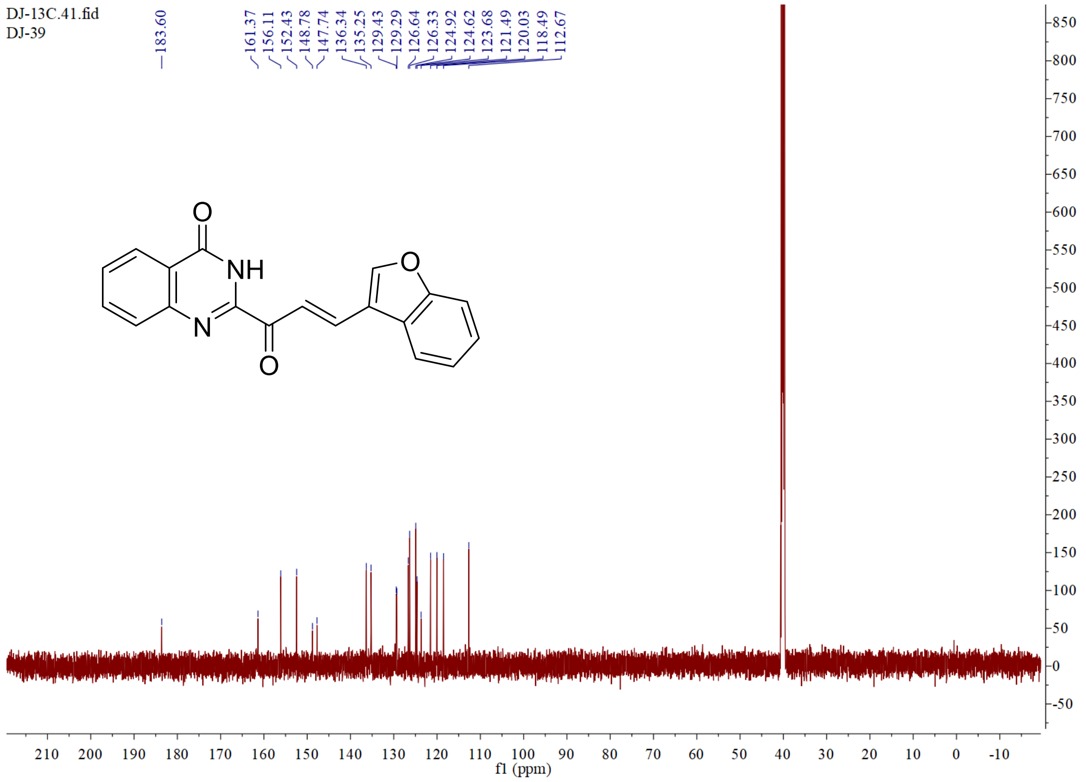

HRMS spectrum

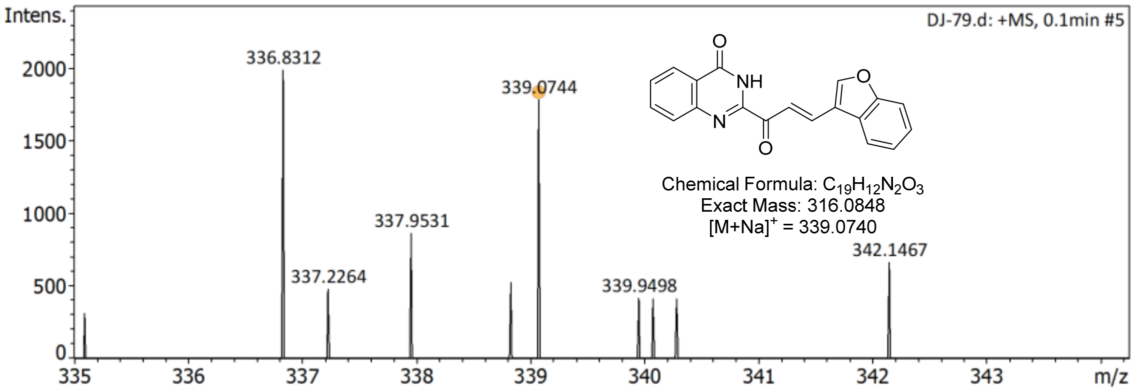

Meas. m/z	#	Ion Formula	m/z	err [ppm]	mSigma	# mSigma	Score	rdb	e¡¥ Conf	N-Rule
339.0744	1	C19H12N2NaO3	339.0740	-1.2	18.6	1	100.00	15.0	even	ok

HPLC spectrum

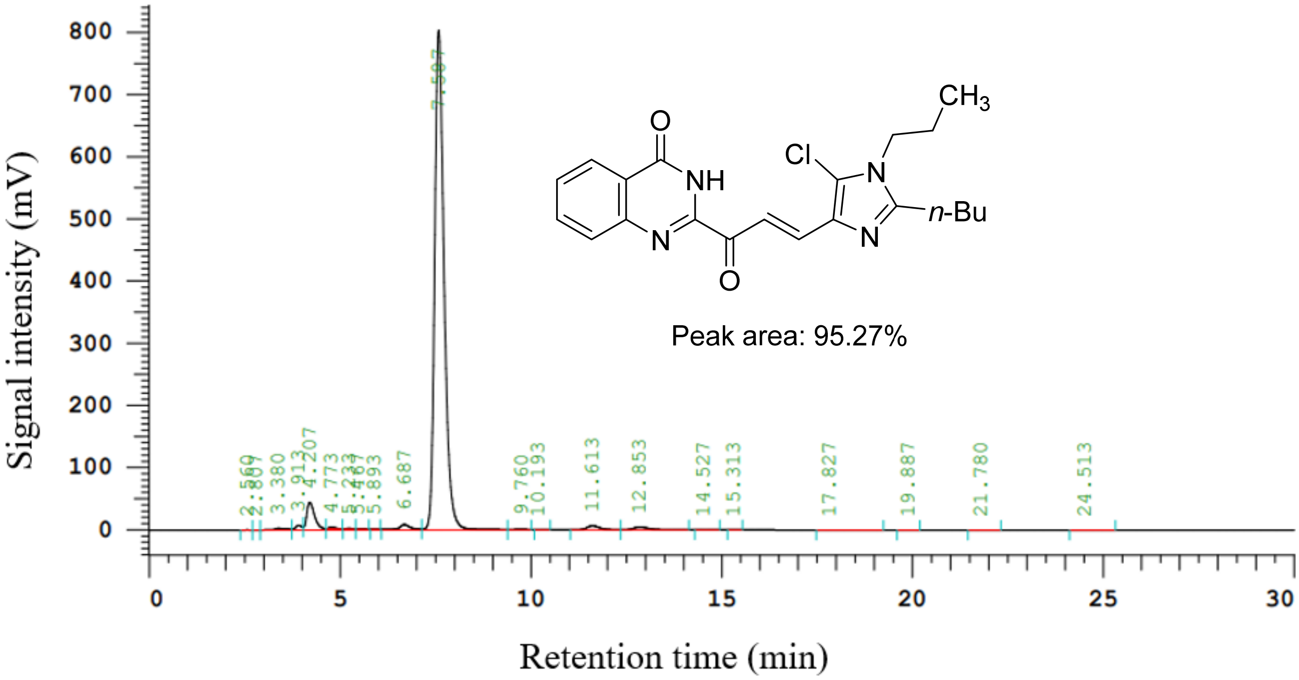

5.10 Spectra of compound **8b**

^{1}H NMR spectrum

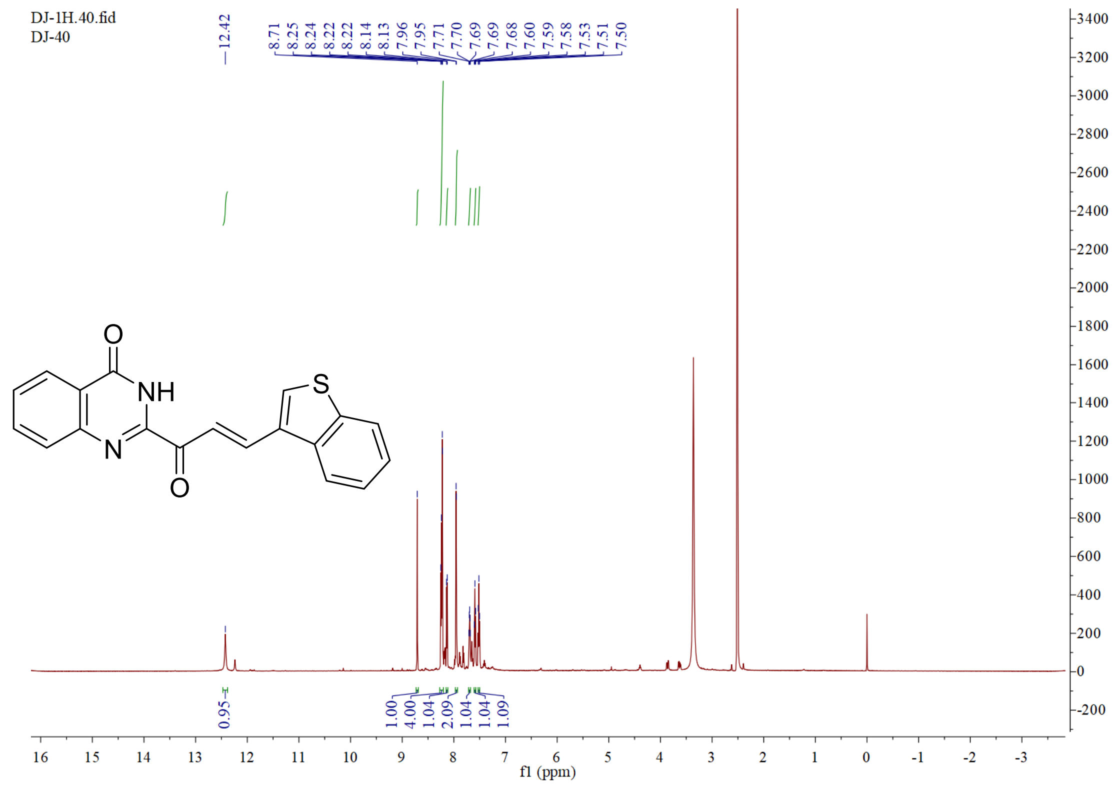

^{13}C NMR spectrum

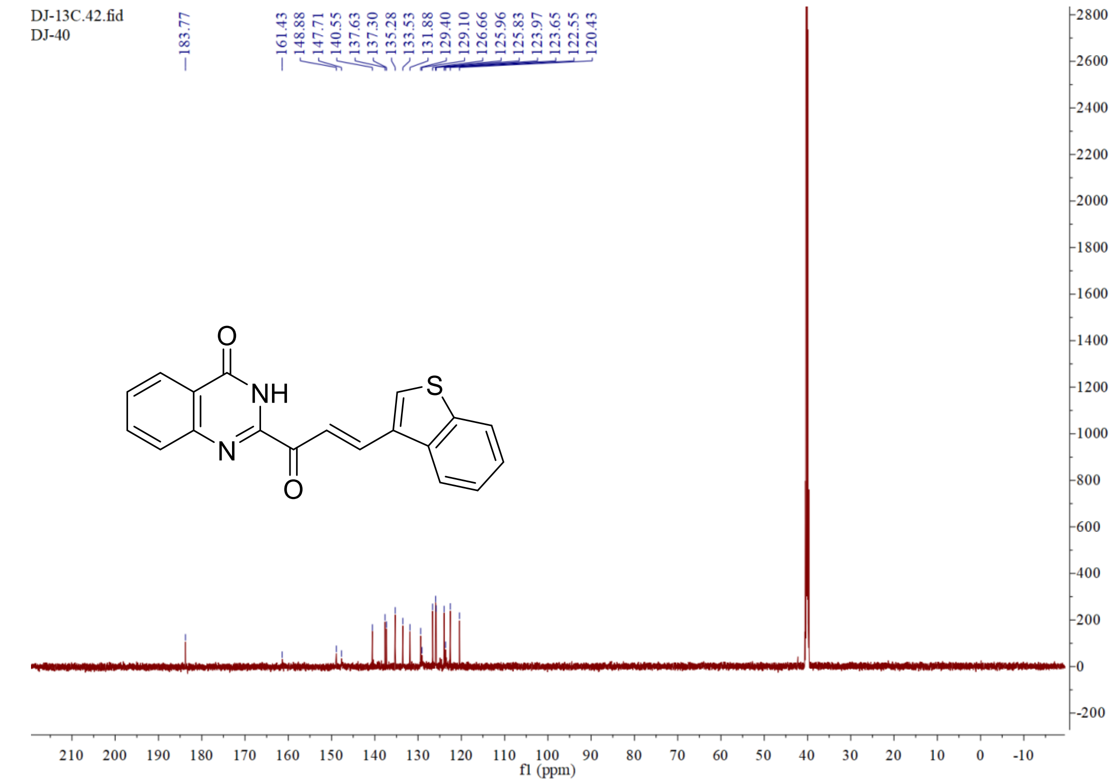

HRMS spectrum

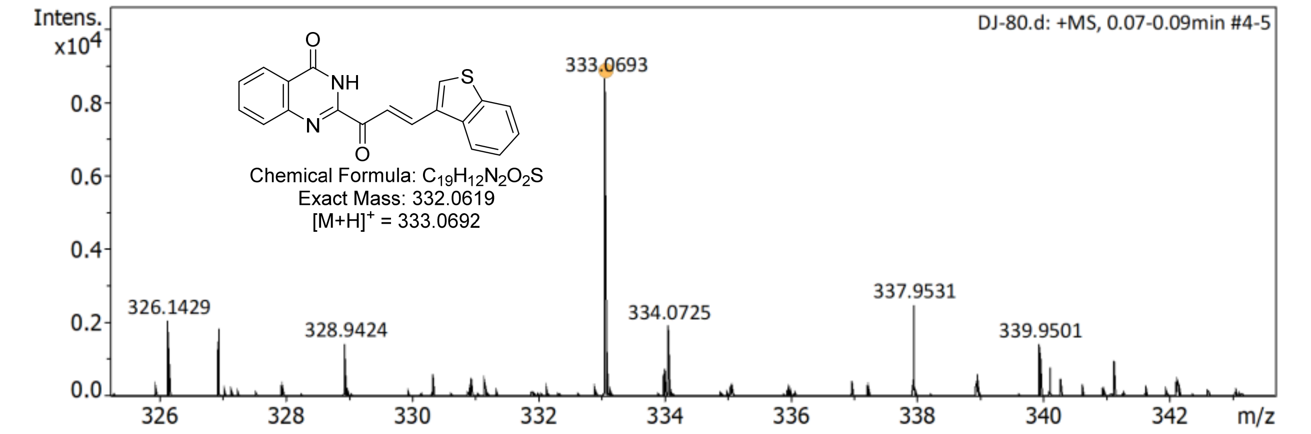

Meas. m/z	#	Ion Formula	m/z	err [ppm]	mSigma	# mSigma	Score	rdb	e¡¥ Conf	N-Rule
333.0693	1	C19H13N2O2S	333.0692	-0.1	n.a.	1	100.00	17.0	even	ok

HPLC spectrum

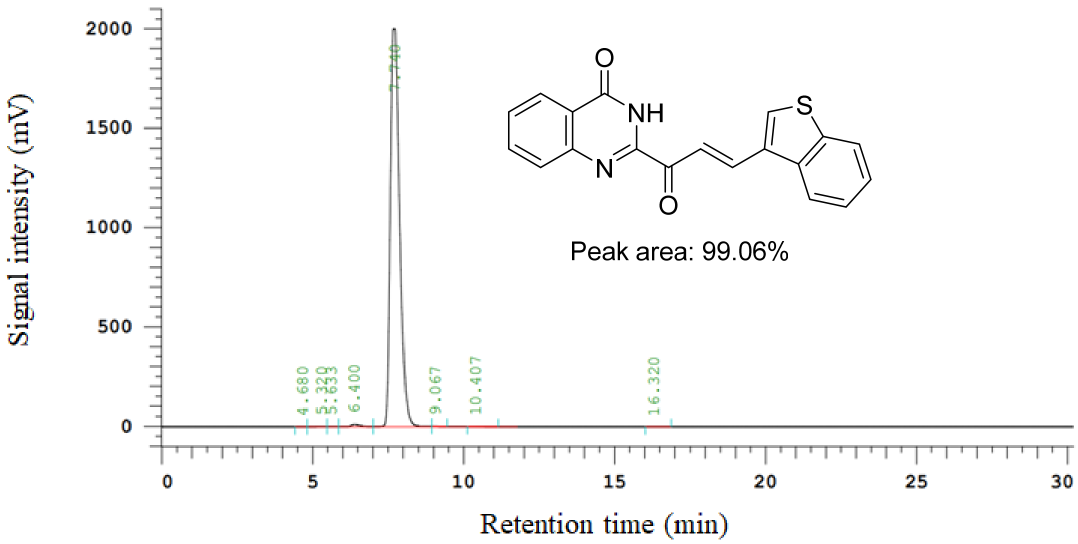

5.11 Spectra of compound **8c**

^{1}H NMR spectrum

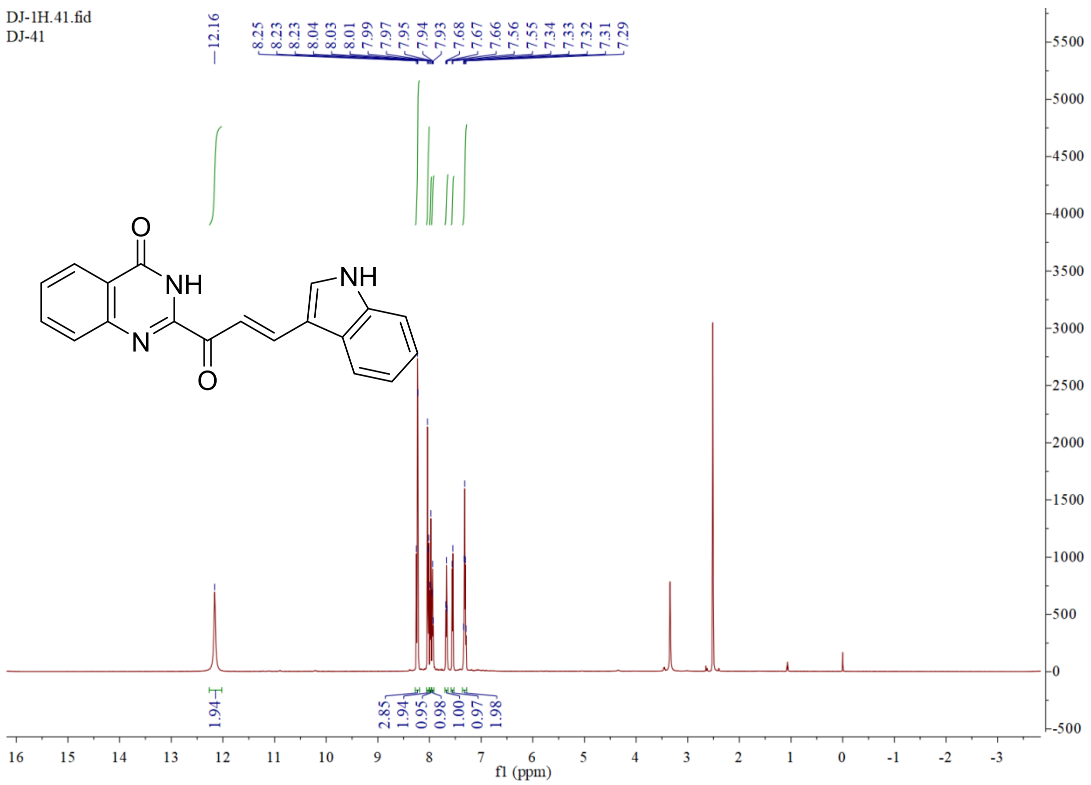

^{13}C NMR spectrum

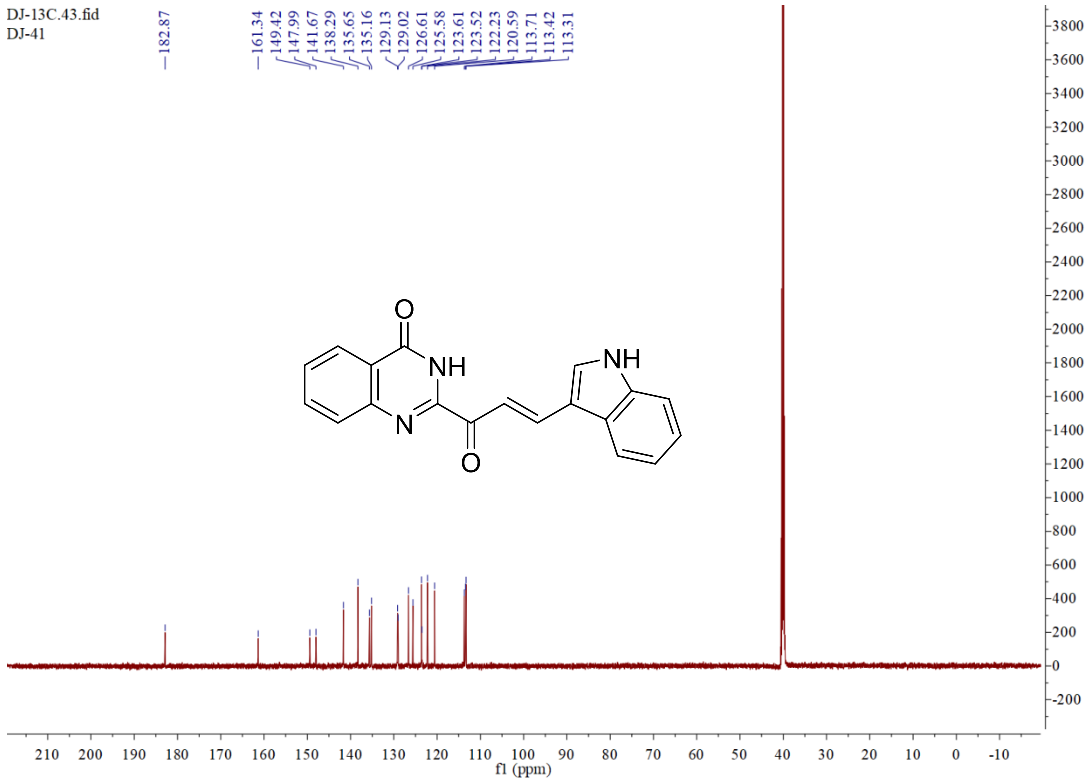

HRMS spectrum

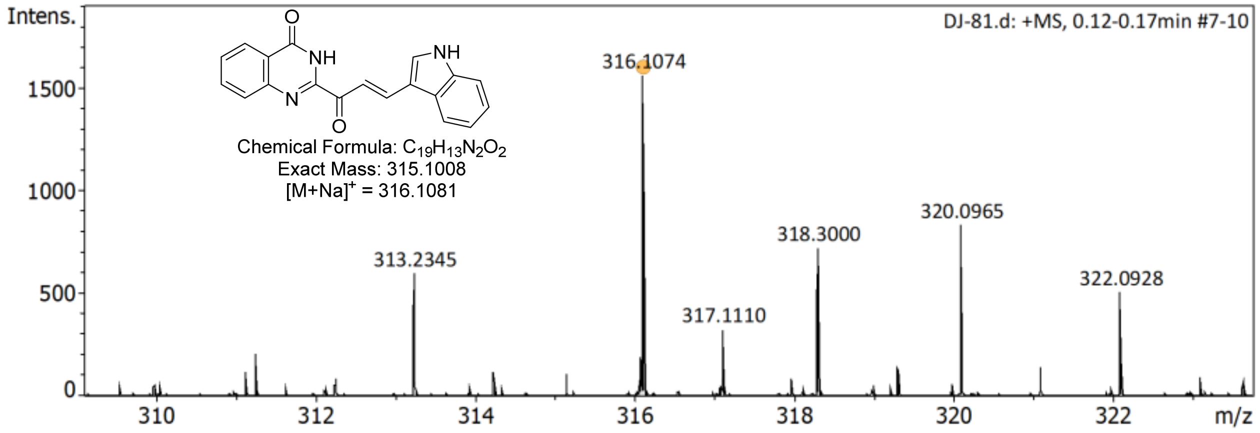

Meas. m/z	#	Ion Formula	m/z	err [ppm]	mSigma	# mSigma	Score	rdb	e¡¥	Conf	N-Rule
316.1074	1	C19H14N3O2	316.1081	2.0	16.6	1	100.00	15.0	even		ok

HPLC spectrum

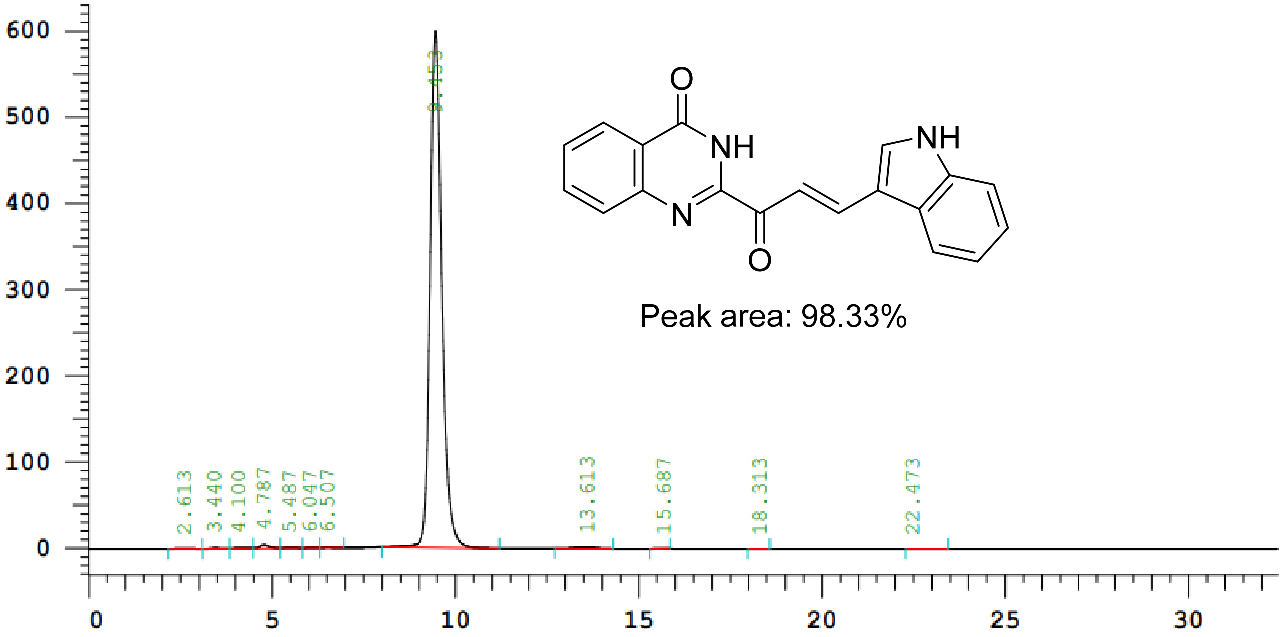

5.12 Spectra of compound **17a**

^{1}H NMR spectrum

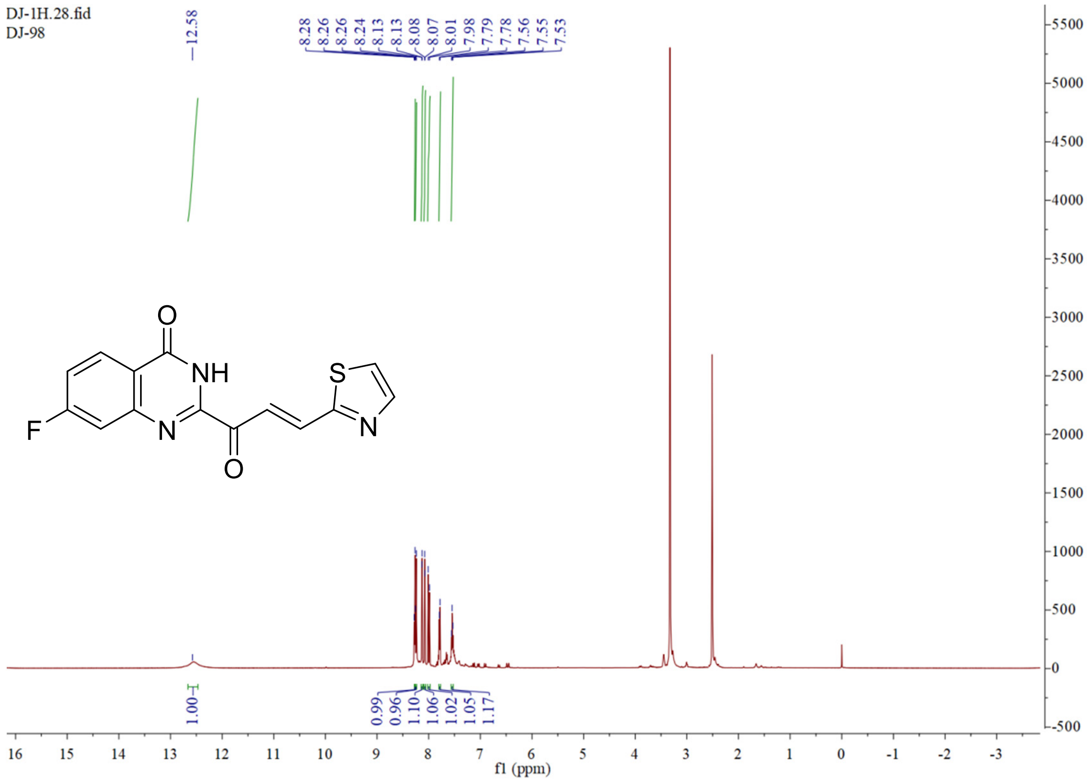

^{13}C NMR spectrum

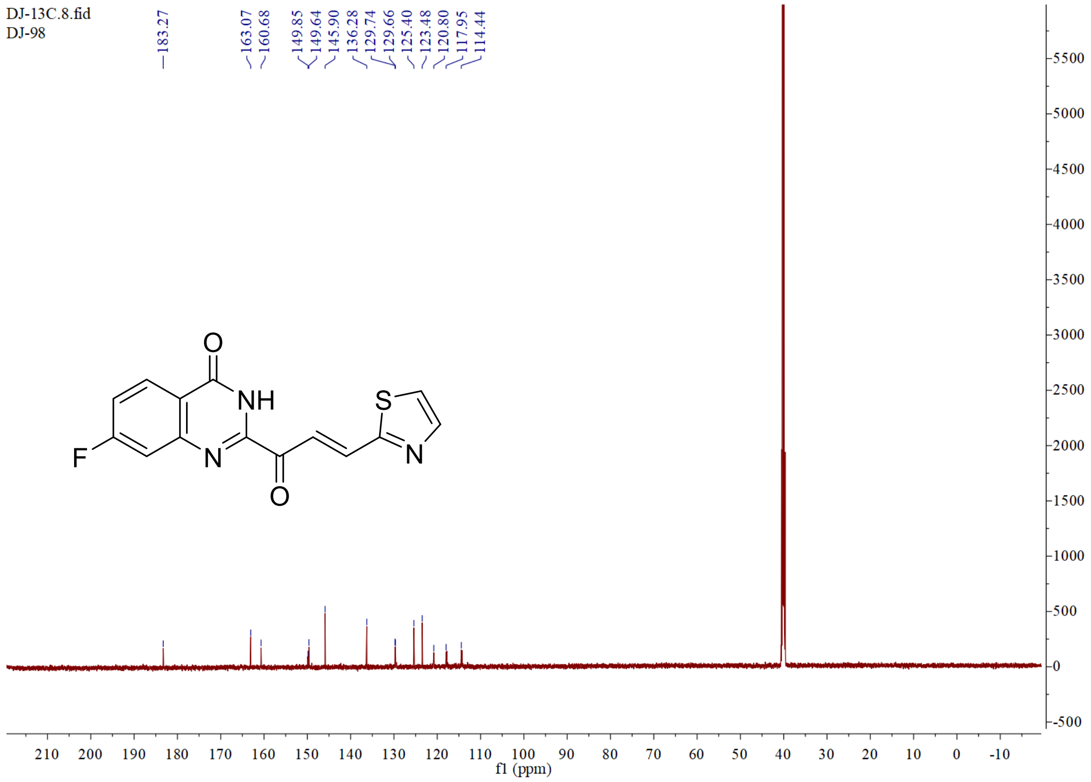

HRMS spectrum

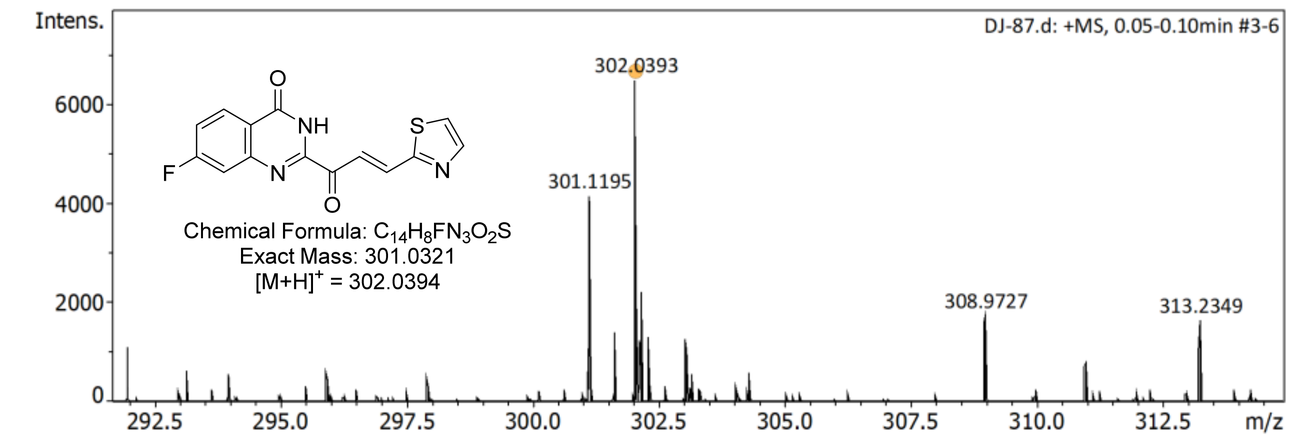

Meas. m/z	#	Ion Formula	m/z	err [ppm]	mSigma	# mSigma	Score	rdb	e¡¥ Conf	N-Rule
302.0393	1	C14H9FN3O2S	302.0394	0.3	34.1	1	100.00	14.0	even	ok

HPLC spectrum

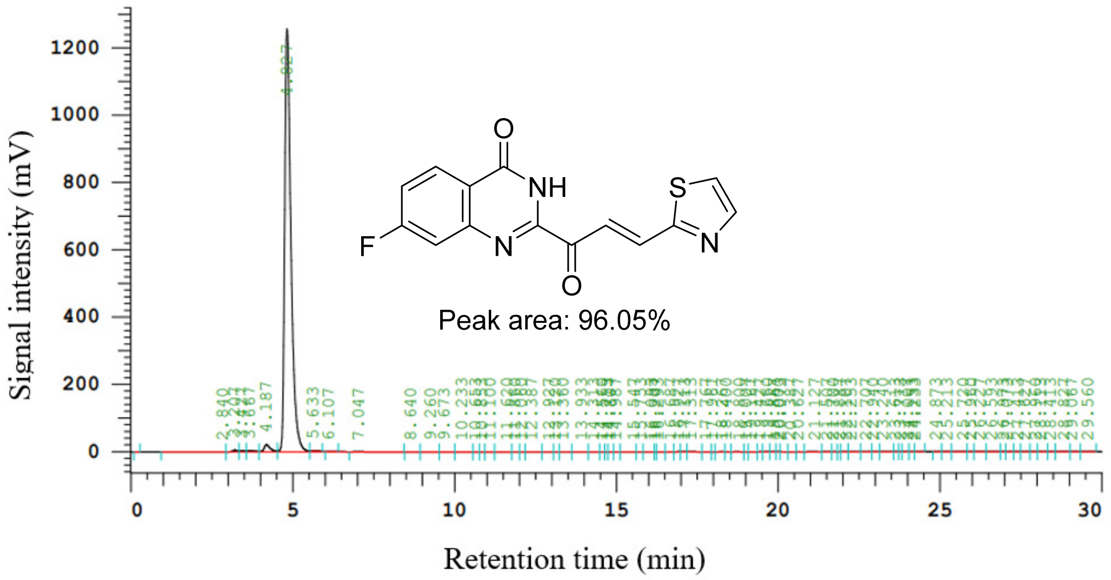

5.13 Spectra of compound **17b**

^{1}H NMR spectrum

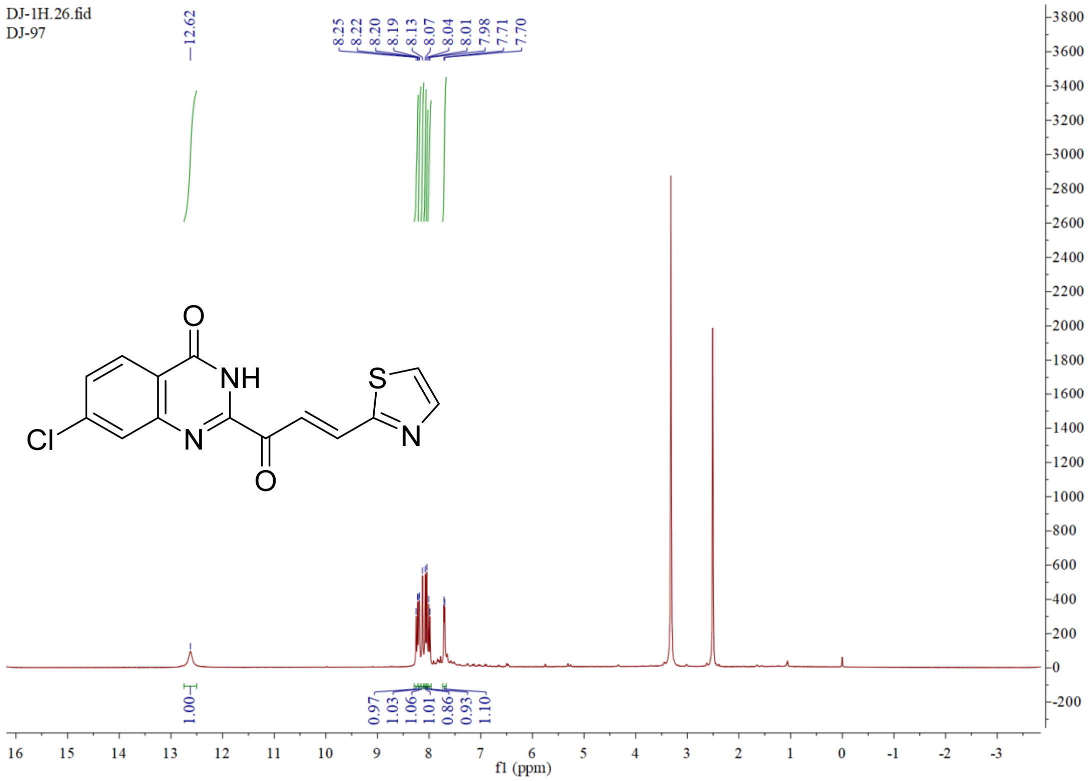

HRMS spectrum

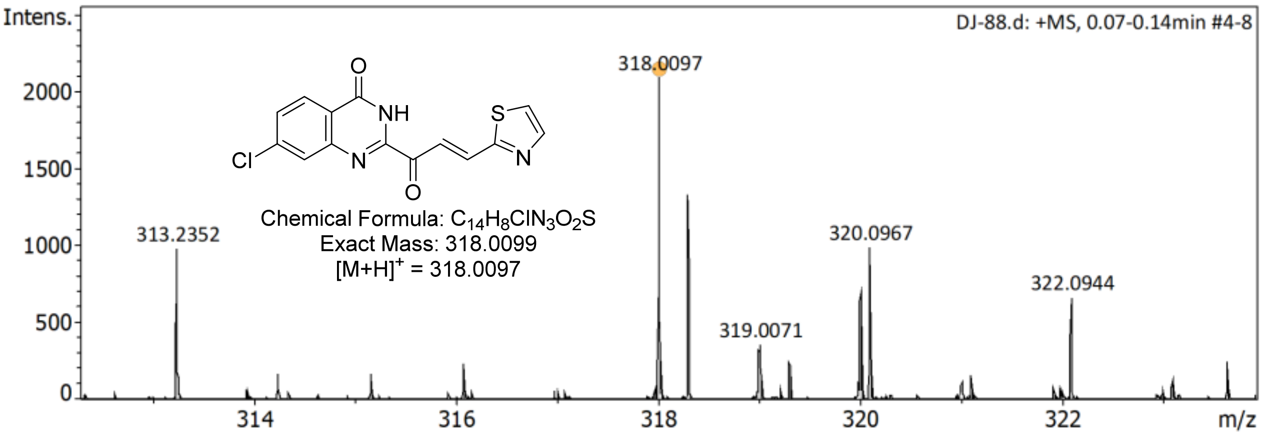

Meas. m/z	#	Ion Formula	m/z	err [ppm]	mSigma	# mSigma	Score	rdb	e¡¥ Conf	N-Rule
318.0097	1	C14H9ClN3O2S	318.0099	0.5	38.1	1	100.00	17.0	even	ok

HPLC spectrum

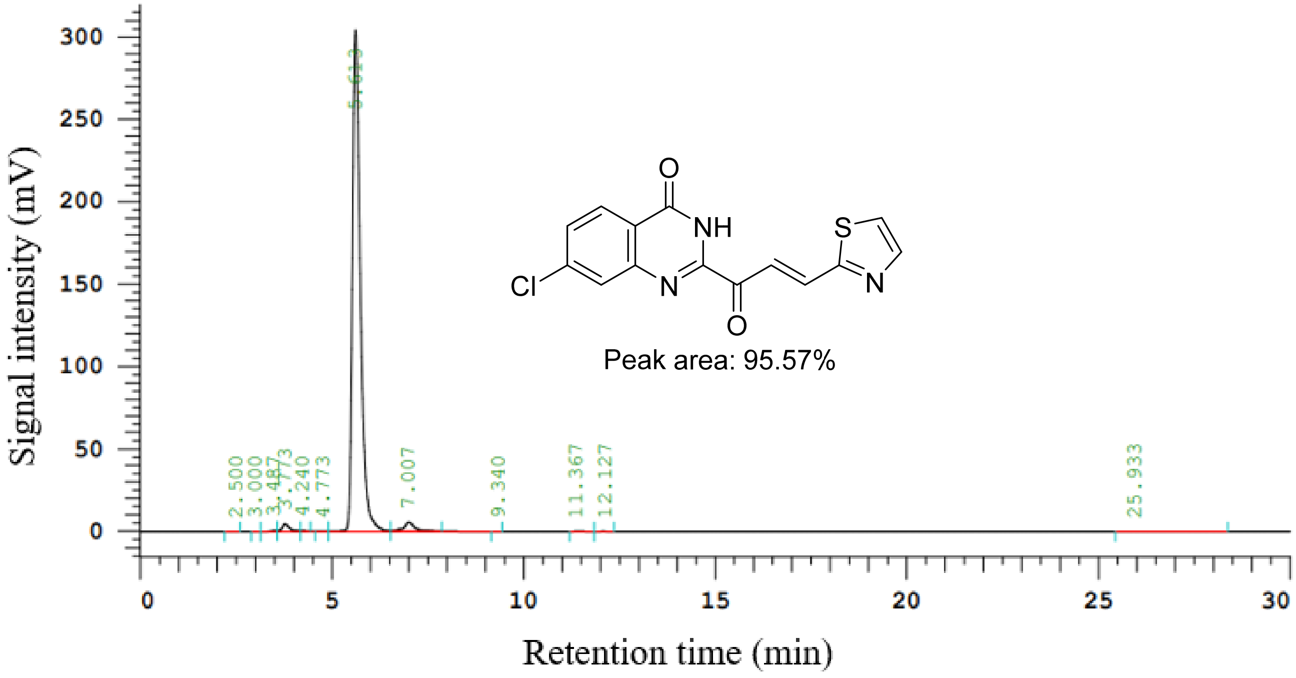

Signal intensity (mV)
300
250
200
150
100
50
0
0
5
10
15
20
25
30
Retention time (min)
2.500
3.000
3.487
3.773
4.240
4.773
7.007
9.340
11.367
12.127
25.933
O
NH
S
Cl
N
O
N
Peak area: 95.57%
